# Random coverage from within with variable radii, and Johnson-Mehl cover times

**DOI:** 10.1007/s10687-026-00529-8

**Published:** 2026-05-25

**Authors:** Mathew D. Penrose, Frankie Higgs

**Affiliations:** Department of Mathematical Sciences, University of Bath, Bath, BA2 7AY UK

**Keywords:** Coverage, Weak limit, Boolean model, Poisson point process, Johnson-Mehl model, Stochastic growth model, 60D05, 60F05, 60G70, 60G55

## Abstract

Given a compact planar region ***A***, let $\boldsymbol{\tau _{A}}$ be the (random) time it takes for the Johnson-Mehl tessellation of ***A*** to be complete, i.e. the time for ***A*** to be fully covered by a spatial birth-growth process in ***A*** with seeds arriving as a unit-intensity Poisson point process in $\boldsymbol{A \times [0,\infty )}$, where upon arrival each seed grows at unit rate in all directions. We show that if $\boldsymbol{\partial A}$ is smooth or polygonal then $\mathbb {P}[\boldsymbol{\pi \tau _{sA}^3 - 6 \log s - 4 \log \log s \le x}]$ tends to **exp**$\boldsymbol{(-(\frac{81}{4\pi })^{1/3} |A|e^{-x/3} - (\frac{9}{2\pi ^2})^{1/3} |\partial {\textbf {A}}| e^{-x/6})}$ in the large-***s*** limit; the second term in the exponent is due to boundary effects, the importance of which was not recognized in earlier work on this model. We present similar results in higher dimensions (where boundary effects dominate). These results are derived using new results on the asymptotic probability of covering ***A*** with a high-intensity spherical Poisson Boolean model *restricted to* ***A*** with grains having iid small random radii, which generalize recent work of the first author that dealt only with grains of deterministic radius.

## Introduction

The *Johnson-Mehl (J-M) tessellation* is a classic model of a random tessellation in $\mathbb {R}^d$, where $d \in \mathbb {N}$. Seeds are generated as a homogeneous Poisson point process $\mathcal{H}_\rho = \{(x_i,t_i)\}_{i \ge 1}$ of intensity $\rho$ in space-time $\mathbb {R}^d \times [0,\infty )$. If location $x_i$ is not already claimed by time $t_i$, the seed *i* becomes a cell at that instant, which immediately starts to grow from $x_i$ at unit rate in all directions, claiming previously unclaimed territory as part of that cell. Whenever the growing cell hits another cell, it stops growing in that direction. Ultimately the whole of $\mathbb {R}^d$ is tessellated by cells.

This model dates back at least to work of Kolmogorov and of Johnson and Mehl in the 1930s on modelling crystallization processes; other applications include growth of surface film on metals, and more recent applications in neurobiology are described in Chiu et al. (2013). For further discussion and references, see also Møller (1992); Okabe et al. (2000).

**Fig. 1:**
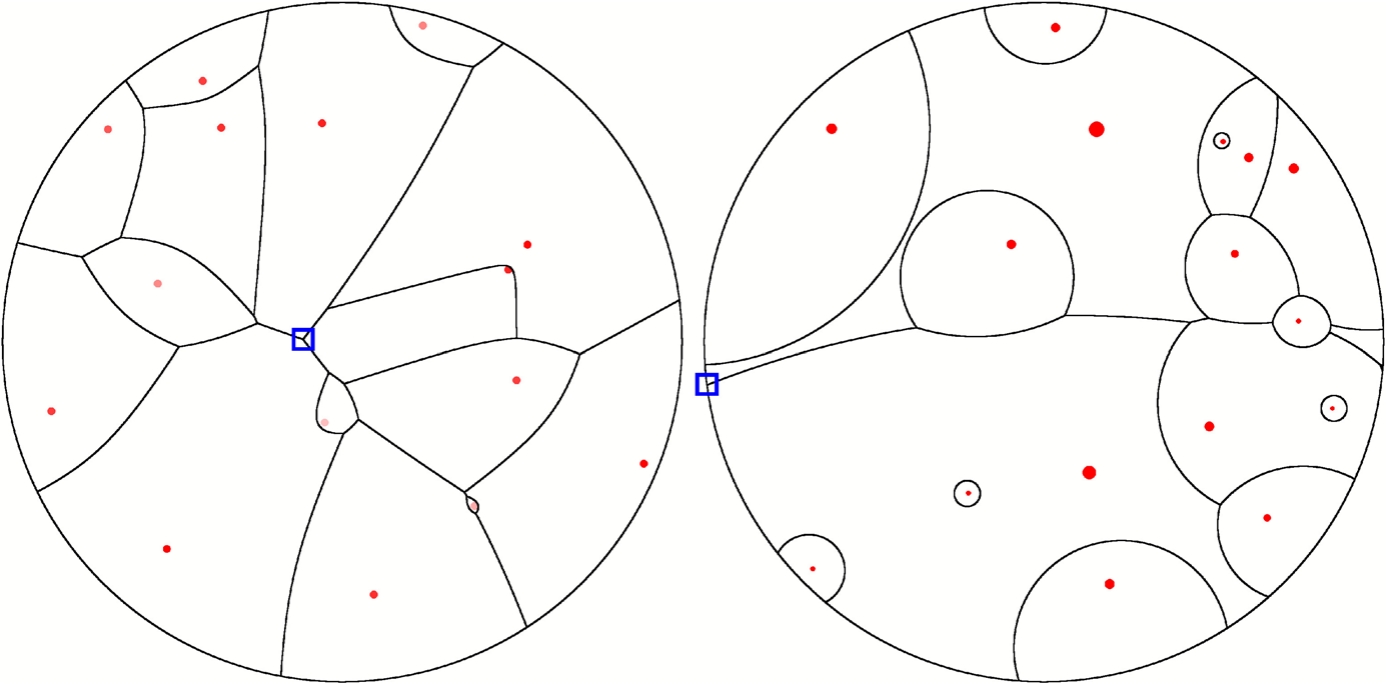
Tessellations of a disc *A* (diameter 0.9) by the restricted Johnson–Mehl process with $\rho \approx 125$ (left) and restricted spherical Poisson Boolean model with $Y_i$ exponentially distributed (right). In both cases the seeds are marked inside each cell with a red dot, and $\textrm{argmax}_{x \in A} \Xi _x$ (the “last location covered”) is marked with a blue square. Later-arriving seeds are marked with paler dots (left diagram); larger dots indicate larger $Y_i$ (right diagram). All vertices of both tessellations are of degree 3, including those on the boundary

A natural variant is the *restricted* Johnson-Mehl tessellation, which we define as follows. Given a specified compact region $A \subset \mathbb {R}^d$, we allow only for seeds that arrive inside the region *A*, that is, the seeds are generated by a Poisson process in $A \times [0,\infty )$. Cells grow by the same rules as described before, and the restrictions of the cells to *A* ultimately tessellate *A*, as shown in Fig. 1.

We are interested here in the time at which *A* becomes completely covered, i.e. the first time at which every point of *A* has been claimed by a cell, either for the J-M model or for the restricted J-M model. In particular we are concerned with the limiting distribution of the cover time, denoted $$\tilde{T}_\rho $$ for the J-M model and $$T_\rho $$ for the restricted J-M model, as $$\rho \rightarrow \infty $$. Equivalently, one can keep $$\rho $$ fixed (say, $$\rho =1$$), and consider the distribution of the cover times for an expanding sequence of windows $$(A_L)_{L>0}$$ given by dilations of a fixed set *A* by a factor of *L*, in the large-*L* limit. In the later case, the restricted Johnson-Mehl tessellation uses only seeds that arrive inside $$A_L $$, and we refer to the cover times in this limiting regime as $$\tau _L$$ (for the restricted J-M model) and $$\tilde{\tau }_L$$ (for the J-M model).

These cover times have previously been considered in Chiu (1995), for $A= [0,1]^d$. In fact, Chiu considers a more general class of J-M models, where the rate at which seeds arrive is allowed to be non-homogeneous in time, and where moreover the seeds do not necessarily all grow at the same rate. We do not consider such generalizations here.

In Chiu (1995), page 893 Chiu rightly distinguishes between the J-M and restricted J-M models. However, he goes on to assert that ‘all theorems in this paper are valid for both models’ because ‘almost surely edge effects do not play a role in the limiting behaviour of $T_L$’ (Chiu’s $T_L$ is equivalent to our $\tau _L$). In other words, Chiu seems to be asserting that $\tau _L$ and $\tilde{\tau }_L$ have the same limiting distribution.

It is our contention that this assertion is incorrect. In dimensions $d \ge 2$, the limiting behaviour of $T_\rho$ is different from that of $\tilde{T}_\rho$, and the limiting behaviour of $\tau _L$ is different from that of $\tilde{\tau }_L$; in other words, edge effects *do* play a role. We justify this assertion with results identifying the limiting distribution of $T_\rho$ (suitably scaled and centred) and showing it is different from that of $\tilde{T}_\rho$. Likewise we show that the limiting distribution of $\tau _L$ (suitably scaled and centred) is different from that of $\tilde{\tau }_L$; our limiting result for $\tilde{\tau }_L$ is consistent with the result in Chiu (1995). Our results for $T_\rho$ and $\tau _L$ apply when *A* is polygonal in $d=2$, or when it has a smooth (more precisely, $C^2$) boundary for general $d \ge 2$.

When $A = [0,1]^d$, we do not provide detailed limiting distributions for $T_\rho$ and $\tau _L$ except in the case $d =2$. To give a detailed limit distribution when $d \ge 3$ would require careful consideration of all faces of all dimensions and is beyond the scope of this paper. However, the time to cover all the $(d-1)$-dimensional faces will be a lower bound for the actual cover time so boundary effects can certainly not be neglected in this case either.

We shall derive our results on the J-M model from new results, of independent interest, on the *spherical Poisson Boolean model (SPBM)*, which is defined to be a collection of Euclidean balls (referred to as *grains*) of i.i.d. random radii, centred on the points of a homogeneous Poisson process in the whole of $\mathbb {R}^d$. For the the *restricted* SPBM, we take a Poisson process on *A* rather than on all of $\mathbb {R}^d$.

We shall determine the probability that the restricted SPBM covers the whole of *A*, in the limit when the Poisson intensity becomes large and the radii of balls become small in a linked manner. In Penrose (2023) we derived results of this nature for balls with deterministic radius; here we generalize them to allow for balls of random radius, which is needed to derive our results on the J-M cover time. Our results on coverage of *A* by the restricted SPBM complement the classic results of Hall (1985); Janson (1986) on the limiting probability of covering *A* with an *unrestricted* SPBM.

The Johnson-Mehl cover time is the maximum of the random field $$(\Xi _x, x \in A)$$, where $$\Xi _x$$ denotes the the time at which at which *x* is covered. For the restricted J-M model, this maximum will be achieved at a vertex *x* of the restricted Johnson-Mehl tessellation of *A*, as illustrated in Fig. 1. (In fact there are other possibilities; e.g. if *A* is polygonal with a sharp corner then $$\sup _{x \in A} \Xi _x$$ could be achieved at a corner of *A* but these other possibilities have vanishing probability in our limit regimes, at least when $$d=2 $$ or $$d=3$$.) Thus it is the maximum of a large finite random number of (perhaps weakly) dependent variables, and one might perhaps expect one of the classical extreme value distributions such as the Gumbel to arise in the limiting regimes that we consider.

It turns out that the limit distribution for the restricted model is indeed Gumbel in 3 or more dimensions, but in two dimensions it is a *two-component extreme value distribution* (see Remark 2.8 below). In short, this is because for $d=2$, the maximum could be achieved either at a vertex in the interior of *A*, or at a vertex on the boundary of *A*, and these different possible contributions scale differently as $\rho \rightarrow \infty$; in higher dimensions, the maximum is very likely to be at the boundary.

For the restricted SPBM, again the probability of coverage can be framed as the extreme value of a random field. Label the Poisson points in *A* as $\{p_1,\ldots ,p_N\}$ and let $Y_i$ be the random radius associated with Poisson point $p_i$. Define the *coverage threshold* *R* to be the smallest *r* such that the union of balls of radius $rY_i$ centred on $p_i$ covers *A*. Then for any scaling factor $r >0$, the probability that that *A* is fully covered by balls of radius $rY_i$ equals the cdf $F_R(r):= \mathbb {P}[R \le r]$. The threshold is the maximum of a random field, again denoted $(\Xi _x, x \in A)$, where now $\Xi _x$ is the smallest *r* such that $x \in \cup _i B(p_i,rY_i)$. Moreover, once again for high intensities the maximum will typically (at least in low dimensions) be achieved at a vertex of a certain tessellation of *A*, namely the division of *A* into cells $C_i, 1 \le i \le N$ with

$$ C_i := \big \{y \in A: \frac{\Vert y-p_i\Vert }{Y_i} \le \frac{\Vert y- p_j \Vert }{Y_j} ~ \forall ~ j \in \{1,\ldots ,N\} \big \}, $$

where $\Vert \cdot \Vert$ denotes the Euclidean norm on $\mathbb {R}^d$; here the cells might not be connected.

See Fig. 1 for an illustration (which does indeed include a disconnected cell) and Remark 2.15 for further discussion (again the cells have a dynamical interpretation). In the special case where the $Y_i$ are a deterministic constant this is simply the *Voronoi* tessellation of *A* induced by the Poisson process in *A*. In fact, in this case the coverage threshold is the largest circumscribed radius of the Poisson-Voronoi tessellation of *A*, as discussed in Penrose (2023) for the restricted SPBM and in Calka and Chenavier (2014) for the unrestricted SPBM.

Thus in all cases we are interested in the distribution of a random variable (either the cover time or the coverage threshold) given by the maximum of a certain geometrically-defined random field on *A*. Other somewhat related topics within this genre of *geometric extreme value theory* (as it might reasonably be termed) include multivariate scan statistics and clique number of random geometric graphs (see e.g. Alm (1998); Penrose (2002)), and largest nearest *k*-neighbour link (*k*-NNL) of a random sample of points (see Penrose and Yang (2025) and references therein in a Euclidean space, Otto and Thäle (2023) in hyperbolic space).

## Statement of results

Throughout this paper, we assume that we are given $d \in \mathbb {N}$, and a compact, Riemann measurable set $A \subset \mathbb {R}^d$ (Riemann measurability of a bounded set in $\mathbb {R}^d$ amounts to its boundary having zero Lebesgue measure).

For $x \in \mathbb {R}^d$ and $r>0$ set $B(x,r):= \{y \in \mathbb {R}^d:\Vert y-x\Vert \le r\}$. (We write $B_{(d)} (x,r)$ for this if we wish to emphasise the dimension.) For $D \subset \mathbb {R}^d$, let $\overline{D}$ and $D^o$ denote the closure of *D* and interior of *D*, respectively, and set $\partial D:= \overline{D} \setminus D^o$, the topological boundary of *D*. For $r>0$, let $D^{(r)}:=\{ x \in D: B(x,r) \subset D^o\}$, the ‘*r*-interior’ of *D*. Let |*D*| denote the Lebesgue measure (volume) of *D*, and $|\partial D|$ the perimeter of *D*, i.e. the $(d-1)$-dimensional Hausdorff measure of $\partial D$, when these are defined. Write $\log \log t$ for $\log (\log t)$, $t >1$.

Define the set $D^{[r]}$ to be the interior of the union of all hypercubes of the form $\prod _{i=1}^d[n_ir,(n_i+1)r]$, with $n_1,\ldots ,n_d \in \mathbb {Z}$, that are contained in $D^o$ (the set $D^{[r]}$ resembles $D^{(r)}$ but is guaranteed to be Riemann measurable).

We say that *D has* $C^2$ *boundary* (for short: $\partial D \in C^2$) if for each $x \in \partial D$ there exists a neighbourhood *U* of *x* and a real-valued function *f* that is defined on an open set in $\mathbb {R}^{d-1}$ and twice continuously differentiable, such that $\partial D \cap U$, after a rotation, is the graph of the function *f*. We say that $\partial D \in C^{1,1}$ (a weaker condition) if for each *x* the function *f* satisfies only that *f* is continuously differentiable with Lipschitz first order partial derivatives.

Given $d \in \mathbb {N}$, let $\omega _d:= \pi ^{d/2}/\Gamma (1 + d/2)$, the volume of the unit ball in $\mathbb {R}^d$, and set

1 $$\begin{aligned} c_d := \frac{1}{d!} \left( \frac{\sqrt{\pi } \; \Gamma (1+ d/2) }{ \Gamma ((d+1)/2) } \right) ^{d-1}. \end{aligned}$$

Note that $$c_1=c_2=1$$, and $$c_3 =3 \pi ^2/32$$. Moreover, using Stirling’s formula one can show that $$c_d^{1/d} \sim e (\pi /(2d))^{1/2}$$ as $$d \rightarrow \infty $$. The constant $$ c_d$$ is denoted $$ \psi _d$$ in Chiu (1995), page 894 while $$\omega _d$$ is denoted $$\theta _d$$ in Penrose (2023). Later it will be useful to have $$\omega _0$$ defined: we set $$\omega _0:=1$$.

### Results for the Johnson-Mehl model

Assume that on a common probability space $(\mathbb {S},\mathcal{F},\mathbb {P})$, we have a family of Poisson point processes $\mathcal{H}_\rho , \rho >0$. Here $\mathcal{H}_\rho$ is homogeneous of intensity $\rho$ on $\mathbb {R}^d \times \mathbb {R}_+$, where $\mathbb {R}_+:= [0,\infty )$. We write $\mathcal{H}_{\rho ,A}$ for $\mathcal{H}_\rho \cap (A \times \mathbb {R}_+)$.

Given $\rho \in (0,\infty )$, define the coverage times $T_{\rho }$ and $\tilde{T}_{\rho }$ by

2 $$\begin{aligned} T_{\rho } := \inf \left\{ t >0: A \subset \cup _{(x,s) \in \mathcal{H}_\rho \cap (A \times [0,t])} B(x,t-s) \right\} ; \end{aligned}$$

3 $$\begin{aligned} \tilde{T}_{\rho } : = \inf \left\{ t >0: A \subset \cup _{(x,s) \in \mathcal{H}_\rho \cap (\mathbb {R}^d \times [0,t])} B(x,t-s) \right\} . \end{aligned}$$

Also, for $L >0$ set $A_L:= \{Lx:x \in A\}$ and let

4 $$\begin{aligned} \tau _{L} := \inf \left\{ t >0: A_L \subset \cup _{(x,s) \in \mathcal{H}_1 \cap (A_L \times [0,t])} B(x,t-s) \right\} ; \end{aligned}$$

5 $$\begin{aligned} \tilde{\tau }_{L} : = \inf \left\{ t >0: A_L \subset \cup _{(x,s) \in \mathcal{H}_1 \cap (\mathbb {R}^d \times [0,t])} B(x,t-s) \right\} . \end{aligned}$$

Then $T_\rho$, $\tilde{T}_\rho$ are the cover times of *A* for the restricted J-M model and for the J-M model, respectively, with intensity $\rho$. Likewise $\tau _L$, $\tilde{\tau }_L$, are the cover time of $A_L$ for the restricted J-M model and for the J-M model, respectively, with intensity 1.

We are concerned with the asymptotic distribution of the cover times $T_{\rho }$ and $\tilde{T}_\rho$ as $\rho \rightarrow \infty$, and the asymptotic distribution of $\tau _{L}$ and $\tilde{\tau }_L$ as $L \rightarrow \infty$. We start with a result in general *d* for the unrestricted cover time $\tilde{T}_{\rho }$ (and for $\tilde{\tau }_L)$. This is simpler to deal with than $T_\rho$ because boundary effects are avoided.

#### Proposition 2.1

Suppose $$A \subset \mathbb {R}^d $$ is compact and Riemann measurable with $$ |A| > 0$$. Let $$\beta \in \mathbb {R}$$. Then

6 $$\begin{aligned}&\lim _{\rho \rightarrow \infty } \mathbb {P}\left[ \omega _d \rho \tilde{T}_{\rho }^{d+1} - d \log \rho - d^2 \log \log \rho \le \beta \right] \nonumber \\&= \exp (-c_d (d^d \omega _d)^{-1/(d+1)} |A|e^{-\beta /(d+1)} ) \end{aligned}$$

7 $$\begin{aligned}&= \lim _{L \rightarrow \infty } \mathbb {P}\left[ \omega _d \tilde{\tau }_L^{d+1}- d (d+1) \log L - d^2 \log \log L - d^2\log (d+1) \le \beta \right] . \end{aligned}$$

In Appendix A, we shall verify that this result is consistent with the case $\gamma = v =1$ of Chiu (1995), Theorem 4. The proof in Chiu (1995) refers to an unpublished preprint; for completeness we shall present the (fairly short) proof of Proposition 2.1 in Section 3.

Our main new results in this subsection are concerned with $T_\rho$ and $\tau _L$, the cover times for the restricted J-M model. We first give a result for the case where *A* is a polygon in $d=2$.

#### Theorem 2.2

Suppose that $d=2$ and *A* is polygonal.

Let $$\beta \in \mathbb {R}$$. Then

8 $$\begin{aligned}&\lim _{\rho \rightarrow \infty } \mathbb {P}[ \pi \rho T_{\rho }^3 - 2 \log \rho - 4 \log \log \rho \le \beta ]\nonumber \\&= \exp ( - (4 \pi )^{-1/3} |A| e^{- \beta /3} - (2 \pi ^2)^{-1/3} |\partial A| e^{-\beta /6} ) \end{aligned}$$

9 $$\begin{aligned}&= \lim _{L \rightarrow \infty } \mathbb {P}[ \pi \tau _L^3 - 6 \log L -4 \log \log L- \log 81 \le \beta ]. \end{aligned}$$

Our result for general *d* and *A* with $C^2$ boundary involves a constant $c'_{d}$ defined by

10 $$\begin{aligned} c'_d := \frac{c_{d-1}\omega _{d-1}^{2d-3}}{\omega _{d-2}^{d-1} d^{d-1}} \Big ( \frac{(d-1)^d}{2 \omega _d^d} \Big )^{(d-1)/(d+1)}. \end{aligned}$$

#### Theorem 2.3

Suppose that $d \ge 2$ and $\partial A \in C^2$ with $\overline{A^o} =A$. Let $\beta \in \mathbb {R}$. If $d=2$ then Eq. 8 and Eq. 9 hold while if $d \ge 3$ then

11 $$\begin{aligned}&\lim _{\rho \rightarrow \infty } \mathbb {P}[ \omega _d \rho T_{\rho }^{d+1} - 2(d-1) \log \rho - 2d(d-1) \log \log \rho \le \beta ] \nonumber \\&= \exp ( - c'_d |\partial A| e^{-\beta /(2d+2)} ) \end{aligned}$$

12 $$\begin{aligned}&= \lim _{L \rightarrow \infty } \mathbb {P}[ \omega _d \tau _L^{d+1} - 2(d^2-1) \log L - 2d(d-1) \log \log L \nonumber \\&~~~~~~~~~~~~~~~~~~~~~~~~~~~~~~~~~~~~~ ~~~~~ - 2 d(d-1) \log (d+1) \le \beta ]. \end{aligned}$$

Let $\textsf{Gu}$ denote a standard Gumbel random variable, i.e. one with cdf $\mathbb {P}[{\textsf{Gu}}\le \beta ] = \exp (- e^{-\beta }), \beta \in \mathbb {R}$. The limit in Eq. 6 is the cdf of the random variable $(d+1) {\textsf{Gu}}+\log (c_d^{d+1}d^{-d}/ \omega _d) + (d+1) \log |A|$, so Proposition 2.1 says that $\omega _d \rho \tilde{T}_\rho ^{d+1}$ suitably centred by a constant depending on $\rho$ and *d* but not *A*, and $\omega _d \tilde{\tau }_L^{d+1}$ suitably centred likewise, both converge in distribution to the random variable $(d+1) ({\textsf{Gu}}+ \log |A|)$.

Similarly, Theorem 2.3 says for $d \ge 3$ that $\omega _d \rho T_\rho ^{d+1}$ suitably centred by a constant depending on $\rho$ and *d* but not *A*, converges in distribution to $(2d+2)( {\textsf{Gu}}+ \log |\partial A| )$, as does $\omega _d \tau _L^{d+1}$.

Theorem 2.2 says for $d=2$ that $\pi \rho T_\rho ^3 - 2 \log \rho - 4 \log \log \rho$, and $\pi \tau _L^3 - 6 \log L - 4 \log \log L - \log 81$, both converge in distribution to the random variable

13 $$\begin{aligned} \max ( 3 ( {\textsf{Gu}}+ \log |A|) - \log (4\pi ) , 6 ({\textsf{Gu}}' + \log |\partial A|) - \log 4 \pi ^4), \end{aligned}$$

where ${\textsf{Gu}}'$ is another standard Gumbel variable, independent of ${\textsf{Gu}}$.

#### Remark 2.4

A series of videos of very high intensity restricted Johnson-Mehl processes inside $A = [0,1]^2$ can be viewed here^1^. The cells are coloured (with five different colours) so that no two adjacent cells in the final “tessellation” have the same colour. To illustrate the above remark about which point is covered last when $d=2$, we have included one video in the playlist where the last point to be covered is at the boundary, and one where it is in the interior.

An interactive tool for generating Johnson-Mehl tessellations is available at https://frankiehiggs.pyscriptapps.com/johnson-mehl-plot/latest.

**Fig. 2:**
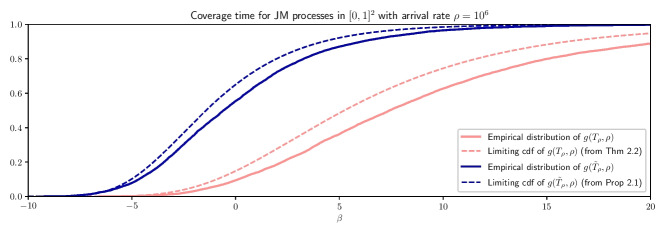
Empirical distributions of the standardised coverage times $T_\rho$ and $\tilde{T}_{\rho }$ as described in Proposition 2.1 and Theorem 2.2, obtained by sampling many independent realisations of the Johnson-Mehl process in $[0,1]^2$. The standardisation function $g(T,\rho ) = \pi \rho T^3 - 2 \log \rho - 4 \log \log \rho$ is the same for both $T_\rho$ and $\tilde{T}_\rho$

The results of more simulations, comparing the distribution of cover times for the restricted and unrestricted J-M models with finite $\rho$ and their limiting distributions, are presented in Fig. 2.

In Chiu (1995), it is suggested that the same limit theorem should apply to $\tau _L$ as to $\tilde{\tau }_L$, as the boundary effects would not affect the limit. This would imply that the same limit should apply to $T_\rho$ as to $\tilde{T}_\rho$. Our Theorem 2.2 shows that in fact the boundary effects do affect the limiting distribution of $g(T_\rho ,\rho )$, and the simulations shown in Fig. 2 back this up.

#### Remark 2.5

One could consider a slight variant of the restricted J-M model, in which a growing cell, whenever it hits the boundary of *A*, stops growing in that direction. Let $T^*_\rho$ denotes the time at which the union of cells first covers *A* for this variant of the model.

When *A* is convex, the value of $T^*_\rho$ is the same as that of $T_\rho$. If *A* is not convex, we would expect our results to still be true for $T^*_\rho$ as well as for $T_\rho$, but we do not prove this.

#### Remark 2.6

Suppose we take $d=2$ and $A= [0,1]^2$. Comparing Eq. 6 with Eq. 8 we see that the limiting distribution of $T_\rho ^3$ (appropriately scaled and centred) is different from that of $\tilde{T}_\rho ^3$ (scaled and centred the same way). Similarly, comparing Eq. 7 with Eq. 9 we see that the limiting distribution of $\tau _L^3$ (appropriately scaled and centred) is different from that of $\tilde{\tau }_L^3$ (scaled and centred the same way).

When $d \ge 3$ and $\partial A \in C^2$, we see from Eqs. 6 and 11 that even the centring constants required to get a nondegenerate limit distribution for $\omega _d \rho T_\rho ^{d+1}$ are different (larger) compared to the corresponding constants for $\omega _d \rho \tilde{T}_\rho ^{d+1}$. Again similar remarks apply for $\tau _L$ and $\tilde{\tau }_L$.

#### Remark 2.7

As mentioned earlier, Chiu (1995) considers cover times for more general J-M models where the growth rate of cells, or the arrival rates of seeds, are not necessarily constants. If growth rates are non-constant, then it is rather problematic to model the covered region at a given time as simply a SPBM; one has to modify our initial description of the cell growth model to allow a faster-growing cell to pass through a slower-growing cell and come out the other side. If one is willing to do this (which seems to be the approach in Chiu (1995)), the methods of this paper should be applicable for dealing with boundary effects for these more general J-M models too.

These more general J-M type models with non-constant growth rates (in particular parabolic growth rates) and non-constant arrival rates, and with the balls allowed to interpenetrate, have been studied in the literature in relation to the understanding of asymptotics of convex hulls of random points (Schreiber and Yukich 2008), maximal points of a multidimensional sample (Schreiber and Yukich 2008) and constructing generalized Delaunay tessellations (Gusakova et al. 2022). It could be interesting to try to map our methods here onto questions of interest in these related models; this is a possible future direction of research.

#### Remark 2.8

The distribution of the maximum of two independent Gumbel variables with different scale parameters (e.g. the variable shown at Eq. 13) is known as a *two-component extreme value* (TCEV) distribution in the hydrology literature (Rossi et al. 1984).

Theorems 2.2 and 2.3 tell us that when $d=2$ and *A* is polygonal or $\partial A \in C^2$, the random variables $\omega _d \rho T_\rho ^{d+1}$ and of $\omega _d \tau _L^{d+1}$, suitably centred, are asymptotically TCEV distributed, while if $d \ge 3$ and $\partial A \in C^2$, they are asymptotically Gumbel distributed.

The TCEV arises elsewhere in geometric extreme value theory. For example, suppose $L_{n,k,d}$ denotes the largest *k*-nearest neighbour link in a sample of *n* uniform random points in $[0,1]^d$, $n > k$. That is, denoting these points $p_1,\ldots ,p_n$, we have $L_{n,k,d} = \max _{1 \le i \le n}( k$-$\min _{j \le d, j \ne n} \Vert p_i-p_j\Vert )$, where *k*-$\min (\cdot )$ means the *k*th smallest of a set of at least *k* numbers. It is shown in Penrose and Yang (2025) that $n \pi L_{n,2,2}^2$, suitably centred, is asymptotically TCEV distributed. Again this is due to boundary effects, and again, these have sometimes been missed in the literature; for example Otto and Thäle (2023), p214 claim, incorrectly, that $n \pi L_{n,2,2}^2$ suitably centred is asymptotically Gumbel. The limiting distribution of $L_{n,k,d}$ (suitably transformed) for general fixed (*k*, *d*) is given in Penrose (2003), Theorem 8.4, which Otto and Thäle (2023) were apparently unaware of.

### Results for the spherical Poisson Boolean model

As discussed in the introduction, we shall derive our results for the J-M model from results on coverage by the restricted SPBM, which are of independent interest and which we now state. Given a nonnegative random variable *Y* and given $n > 0$, suppose we have a collection of balls of independent random radii with the distribution of *Y*, centred on the points of a homogeneous Poisson point process in *A* of intensity *n*.

Given also $k \in \mathbb {N}$, let $Z_{A,k}(n,Y)$ be the set of points $x \in \mathbb {R}^d$ such that *x* is covered by at least *k* of the balls, so in particular $Z_{A,1}(n,Y)$ is the union of the balls (we shall provide a more detailed description of $Z_{A,k}(n,Y)$ in Section 4.2.) Throughout this paper, *n* is not necessarily an integer.

#### Theorem 2.9

(Limiting probability of *k*-coverage of a polygonal domain by a restricted SPBM) Suppose *Y* is a nonnegative random variable with $0< \mathbb {E}[Y^{2+\varepsilon }] < \infty$ for some $\varepsilon >0$. Suppose that $d=2$ and *A* is polygonal. Let $k \in \mathbb {N}, \beta \in \mathbb {R}$, and suppose $(r_n)_{n >0}$ are nonnegative numbers satisfying $n \pi r_n^2 \mathbb {E}[Y^2] - \log n - (2k-1) \log \log n \rightarrow \beta$ as $n \rightarrow \infty$. Then as $n \rightarrow \infty$,

14 $$\begin{aligned} \mathbb {P}[ A \subset Z_{A,k}(n,r_nY)] \rightarrow \exp \Big ( - \Big ( \frac{(\mathbb {E}[Y])^2}{\mathbb {E}[Y^2]} \Big ) \textbf{1}_{\{k=1\}} |A| e^{- \beta } - \Big ( \frac{c_{2,k} \mathbb {E}[Y] |\partial A| }{(\mathbb {E}[Y^2])^{1/2}} \Big ) e^{-\beta /2} \Big ), \end{aligned}$$

where we set $c_{2,k}:= \pi ^{-1/2}(1/2)^{k-1}/(k-1)!$.

For general $d, k \in \mathbb {N}$ with $d \ge 2$ we define $c_{d,k}$ (as in Penrose (2023)) by

15 $$\begin{aligned} c_{d,k}&:= c_{d-1} \omega _d^{2-d-1/d} \omega _{d-1}^{2d-3} \omega _{d-2}^{1-d} (1- 1/d)^{d+k -3 + 1/d} 2^{-1+1/d} /(k-1)!. \end{aligned}$$

It can be checked that

16 $$\begin{aligned} c_{d,1} = ((d-1)!)^{-1}2^{1-d} (d-1)^{d-2+1/d} \pi ^{(d/2)-1} \Gamma \left( \frac{d+1}{2} \right) ^{1-d} \Gamma \left( \frac{d}{2} \right) ^{d - 1 + 1/d }, \end{aligned}$$

and $c_{d,k} = c_{d,1} (1-1/d)^{k-1}/(k-1)!$. Also $c_{2,1} = \pi ^{-1/2}$ and $c_{3,1}= 2^{-4} \pi ^{5/3}$. In the right hand side of Eq. 16 the first factor of $((d-1)!)^{-1}$ was given, incorrectly, as $(d!)^{-1}$ in Penrose (2023).

#### Theorem 2.10

(Limiting probability of *k*-coverage of a smoothly bounded region in $d\ge 2$ by a restricted SPBM) Suppose that $d \ge 2$ and $\partial A \in C^2$ with $\overline{A^o} =A$. Suppose *Y* is a nonnegative random variable with $0<\mathbb {E}[ Y^{d+ \varepsilon } ] < \infty$ for some $\varepsilon >0$. Let $k \in \mathbb {N}, \beta \in \mathbb {R}$, and suppose $(r_n)_{n >0}$ are nonnegative numbers satisfying

17 $$\begin{aligned} n \omega _d r_n^d \mathbb {E}[Y^d] - (2-2/d) \log n - 2(d+k-3+1/d) \log \log n \rightarrow \beta \end{aligned}$$

as $n \rightarrow \infty$. If $d=2$ then Eq. 14 holds, while if $d \ge 3$ then

18 $$\begin{aligned} \lim _{n \rightarrow \infty } \mathbb {P}[ A \subset Z_{A,k}(n,r_nY)] = \exp \Big ( - c_{d,k} \Big (\frac{(\mathbb {E}[Y^{d-1}])^{d-1}}{(\mathbb {E}[Y^{d}])^{d-2+1/d}} \Big ) |\partial A| e^{-\beta /2} \Big ). \end{aligned}$$

#### Remark 2.11

Taking $Y \equiv 1$ in these results gives Theorem 3.2 and Theorem 3.1 of Penrose (2023).

That paper also includes parallel results (in the case $Y \equiv 1$) for a situation where the number of grains in the spherical Boolean model is a large deterministic constant rather than a Poisson variable. We expect that a result along these lines

could be given for general *Y* using the methods of this paper.

#### Remark 2.12

The condition $\overline{A^o}=A$ should have been included in the statement of Penrose (2023), Theorem 3.1. For example, if $d=2$ and *A* is the union of a disk (filled in) and a circle (not filled in, and disjoint from the disk) then that theorem does not apply.

#### Remark 2.13

In Theorem 2.10, we conjecture that the condition $\partial A \in C^2$ can be relaxed to $\partial A \in C^{1,1}$.

#### Remark 2.14

If $\mathbb {E}[Y^d]= \infty$ then $\mathbb {P}[A \subset Z_{\mathbb {R}^d,k}(n,rY)] =1$ for any $k \in \mathbb {N}$ and any $r>0$; see e.g. Last and Penrose (2017), Theorem 16.4 for the case $k=1$. However, one could still look for a sequence $(r_n)$ such that $\mathbb {P}[A \subset Z_{A,1}(n,r_nY)]$ converges to a nontrivial limit. It seems plausible in this case that *A* is most likely to be covered (if at all) by a small number of balls. This looks like an interesting problem that lies beyond the scope of this paper.

#### Remark 2.15

A series of videos illustrating the SPBM and Remark 2.14 can be viewed at this link^2^. There are three videos, with $n = 1000$, $n=10000$ and $n=20000$ respectively. Each video has a Poisson point process of intensity *n* inside $[0,1]^2$ with points $\{ p_1, \dots , p_{N_n} \}$. The frame at time *t* is an image of a tessellation corresponding to a restricted SPBM, $Z_{A,1}(n,r_t Y_t)$, where $A = [0,1]^2$, $Y_t$ is Pareto distributed with shape parameter $\alpha (t)$ and $r_t$ is chosen so that $A \subset Z_{A,1}(n,r_t Y_t)$. There are $N_n$ cells in the frame: the pixel with centre $x \in [0,1]^2$ is assigned to a cell based on $\textrm{argmin}_{i}\inf \{r \ge 0: x \in B(p_i,r Y_{t,i})\}$, where $(Y_{t,i})_{1 \le i \le N_n}$ are independent copies of $Y_t$. In other words, for *t* fixed the tessellation is generated by a growth process

for which all seeds are born at time 0, growth rates are random and distributed like $Y_t$, and the growing cells are interpenetrable.

Note that cells are not necessarily connected. When displaying the video we colour the cells so that no two adjacent cells have the same colour, although two distinct cells which never touch are allowed to have the same colour.

Over the video, $\alpha (t)$ changes from 3.00 to 0.25, i.e. $Y_t$ becomes more heavy-tailed, with the $Y_t$s coupled using the definition $Y_t:= U^{-1/\alpha (t)}$ where *U* is uniformly distributed on [0, 1].

At the beginning of each video, $\mathbb {E}[Y_t^{3-\varepsilon }] < \infty$ for all $\varepsilon > 0$ so Theorem 2.9 applies. As the video progresses, we gradually reduces the number of finite moments, finishing when $\mathbb {E}[Y_t^{0.25}] = \infty$.

The videos after the point where $\mathbb {E}[Y_t^2] = \infty$ illustrate Remark 2.14. In the period when $\mathbb {E}[Y_t^2] = \infty$ but $\mathbb {E}[Y_t^{7/4}] < \infty$, there is not yet a single cell containing more than half the area of $[0,1]^2$, indicating that there may be a non-trivial period where several balls are required to cover $[0,1]^2$.

### Strategy of proof

Next, we briefly describe the strategy for the proof, in Sections 3 and 4, of the weak convergence results that were stated above. In both sections, we shall use a known result (Lemma 3.1) giving the limiting probability of covering a bounded region of $\mathbb {R}^d$ by an unrestricted SPBM, in the limit of high intensity *n* and small balls of random radius, i.e. distributed as $r_nY$ for a specified nonnegative random variable *Y* and for constants $r_n$ that become small as *n* becomes large.

We claim that the question of coverage for the (restricted) SPBM with uniformly distributed radii maps onto the same question for the restricted Johnson-Mehl model. Indeed, given $t > 0$, consider a SPBM in *A* with Poisson intensity *n* and radii uniformly distributed over [0, *t*]. Label the centres $x_1,\ldots ,x_N$ with associated radii $t_1,\ldots ,t_N$. Then for each *i*, $t_i$ is uniformly distributed over [0, *t*] and therefore so is $t-t_i$. Hence by the Marking theorem (see e.g. Last and Penrose (2017)), the point process $\eta := \{(x_i,t - t_i), 1 \le i \le N\}$ is a Poisson process in $A \times [0,t]$ with intensity given by the product of Lebesgue measure on *A* and the uniform probability distribution on [0, *t*], i.e. *n*/*t* times Lebesgue measure on $A \times [0,t]$. Hence the covered region for the original SPBM in *A* is the same as the covered region for the restricted J-M model (run up to time *t*) obtained by using the Poisson process $\eta$. We can argue similarly in the unrestricted case too.

Using this claim and applying Lemma 3.1 for the SPBM with *Y* uniformly distributed on [0, 1] will yield a proof of Proposition 2.1. Similarly, applying Theorems 2.9 and 2.10 with uniform *Y* will yield proofs of Theorems 2.2 and 2.3 respectively.

Our strategy for proving Theorems 2.9 and 2.10 goes as follows. We shall consider, for large *n* and suitable choice of $r_n$, the SPBM with radii distributed as $r_nY$, restricted to a *d*-dimensional half-space $\mathbb {H}$. In Lemma 4.4 we determine the limiting probability that a given bounded set within the hyperplane $\partial \mathbb {H}$ is covered, by applying Lemma 3.1 in $d-1$ dimensions. Moreover we will show that the probability that a region in the half-space within distance $n^{\zeta }r_n$ of that set is covered with the same limiting probability, where ${\zeta }$ is a small positive constant.

We shall then prove Theorem 2.9 by applying Lemma 4.4 to determine the limiting probability that the region near the edges of a polygonal set *A* is covered, and Lemma 3.1 directly to determine the limiting probability that the interior region is covered, along with a separate argument to show the regions near the corners of *A* are covered with high probability.

To prove Theorem 2.10 we approximate to *A* by a polytopal set $A_n$ with faces of width $O(n^{9 {\zeta }} r_n)$ and follow similar steps to those just mentioned for polygonal *A*.

In the case where *Y* is unbounded, to obtain the required independence, for example between coverage events for different faces of our polyhedron or polygon, we consider only those balls of radius at most $n^\zeta r_n$, with a separate argument (Lemma 4.2) to show this does not affect the limiting coverage probabilities.

## Proof of results for the J-M model

We now use Theorems 2.9 and 2.10 to prove Theorems 2.2 and 2.3. The proof of Theorems 2.9 and 2.10 is much longer and we defer this to the next section.

We shall repeatedly use the following result from Penrose (2023), which is based on results in Janson (1986) or Hall (1985). Recall that $c_d$ and $Z_A(n,Y)$ were defined at Eq. 1 and just before Theorem 2.9 respectively.

### Lemma 3.1

(Limiting probability of coverage by an unrestricted SPBM) Let $d \in \mathbb {N}$. Let $\alpha := \omega _d \mathbb {E}[Y^d]$. Let $\beta \in \mathbb {R}$. Suppose $\delta (\lambda ) \in (0,\infty )$ is defined for all $\lambda >0$, and satisfies

19 $$\begin{aligned} \lim _{\lambda \rightarrow \infty } \left( \alpha \delta (\lambda )^d \lambda - \log \lambda - (d+k-2) \log \log \lambda \right) = \beta . \end{aligned}$$

Let $B \subset \mathbb {R}^d$ be compact and Riemann measurable, and for each $\lambda >0$ let $B_\lambda \subset B$ be Riemann measurable with the properties that $B_\lambda \subset B_{\lambda '}$ whenever $\lambda \le \lambda '$, and that $\cup _{\lambda >0} B_\lambda \supset B^o$. Then

20 $$\begin{aligned} \lim _{\lambda \rightarrow \infty } \mathbb {P}[B_\lambda \subset Z_{\mathbb {R}^d,k}(\lambda ,\delta (\lambda )Y) = \exp \left( - \left( \frac{c_d (\mathbb {E}[Y^{d-1}] )^d}{ (k-1)!(\mathbb {E}[Y^d ])^{d-1} } \right) |B| e^{-\beta } \right) . \end{aligned}$$

### Proof

See Penrose (2023), Lemma 7.2. There, it was assumed that *Y* is bounded, but the proof carries over if this condition is relaxed to the $(d+\varepsilon )$-th moment condition used here. $\square$

### Lemma 3.2

(Scaling lemma) Suppose $L >0$ and $\rho = L^{d+1}$. Then $L \tilde{T}_\rho$ has the same distribution as $\tilde{\tau }_L$. Moreover $L T_\rho$ has the same distribution as $\tau _L$.

### Proof

For any $$ t > 0$$, setting $$t' = Lt$$, we have

$$\begin{aligned} A \subset \cup _{(x,s) \in \mathcal{H}_\rho \cap (\mathbb {R}^d \times [0,t])} B(x,t-s) \Leftrightarrow A_L \subset \cup _{(y,u) \in L \mathcal{H}_\rho \cap (\mathbb {R}^d \times [0,t'])} B(y,t'-u) \end{aligned}$$

so that by Eq. 3,

$$\begin{aligned} L \tilde{T}_\rho = \inf \{ t': A_L \subset \cup _{(y,u) \in L \mathcal{H}_\rho \cap (\mathbb {R}^d \times [0,t'])} B(y,t'-u) \}. \end{aligned}$$

By our choice of $\rho$ and the Mapping theorem (see e.g. Last and Penrose (2017)), $L \mathcal{H}_\rho$ is a homogeneous Poisson process of intensity 1 on $\mathbb {R}^d \times \mathbb {R}_+$, and therefore by the definition Eq. 5, $L \tilde{T}_\rho$ has the same distribution as $\tilde{\tau }_L$.

The proof that $L T_\rho$ has the same distribution as $\tau _L$ is similar. $\square$

### Proof of Proposition 2.1

Suppose for some $\beta \in \mathbb {R}$ that $(t_\rho )_{\rho >0}$ satisfies

21 $$\begin{aligned} \lim _{\rho \rightarrow \infty } \left( \omega _d \rho t_\rho ^{d+1} - d \log \rho - d^2 \log \log \rho \right) = \beta , \end{aligned}$$

i.e. $$t_\rho = \left( \left( d\log \rho + d^2 \log \log \rho + \beta + o(1)\right) /( \omega _d \rho )\right) ^{1/(d+1)}$$.

Write $\mathcal{H}_{\rho } \cap (\mathbb {R}^d \times [0,t_\rho ]) = \{(X_i,S_i)\}_{i \in \mathbb {N}}$. Then the point process $\mathcal{P}_{\rho t_\rho } := \{X_i\}_{i \in \mathbb {N}}$ is a homogeneous Poisson process of intensity $\rho t_\rho$ in $\mathbb {R}^d$.

The balls $B(X_i,t_\rho -S_i)$, $i \in \mathbb {N},$ form a SPBM in $\mathbb {R}^d$, and in the notation of Lemma 3.1, here we have $\delta (\lambda ) = t_\rho$, and *Y* uniformly distributed over [0, 1], so that $\alpha =\omega _d/(d+1)$, and $\lambda = \rho t_\rho$, so that

$$ \log \lambda = \left( \frac{d}{d+1} \right) \log \rho + \frac{\log \log \rho + \log (d/\omega _d)}{d+1} + o(1), $$

and $$\log \log \lambda = \log \log \rho + \log (d/(d+1)) + o(1)$$. Hence,

$$\begin{aligned} \alpha \delta (\lambda )^d \lambda - \log \lambda - (d-1) \log \log \lambda&= \frac{\omega _d \rho t_\rho ^{d+1} }{d+1} - \frac{d \log \rho }{d+1} - \frac{ d^2 \log \log \rho }{d+1} - \frac{\log (d/\omega _d)}{d+1} \nonumber \\&~~~~~~~ - (d-1) \log \left( \frac{d}{d+1} \right) + o(1), \end{aligned}$$

and by Eq. 21, as $\rho \rightarrow \infty$ this tends to

$$ \frac{\beta }{d+1} - \log \left( \left( \frac{d^{d^2}}{\omega _d (d+1)^{d^2 -1}} \right) ^{1/(d+1)} \right) . $$

Hence we have the condition (19) from Lemma 3.1 (with $\beta$ on the right hand side replaced by the last displayed expression). Also $\mathbb {E}[Y^{d-1}] = 1/d$ and $\mathbb {E}[Y^d] = 1/(d+1)$.

We have $\tilde{T}_\rho \le t_\rho$ if and only if $A \subset \cup _{i\in \mathbb {N}}B(X_i,t_\rho -S_i)$. Therefore by taking $k=1$ and $B_\lambda = A$ for all $\lambda$ in Lemma 3.1, we obtain that

$$ \mathbb {P}[\tilde{T}_{\rho } \le t_\rho ] \rightarrow \exp \left( - c_d \left( \frac{(d+1)^{d-1}}{d^d} \right) \left( \frac{d^{d^2}}{\omega _d (d+1)^{d^2-1} } \right) ^{1/(d+1)} |A|e^{-\beta /(d+1)}\right) . $$

This yields (6).

For Eq. 7, take $\rho = L^{d+1}$, so that $\log \log L = \log \log \rho - \log (d+1)$. By Lemma 3.2, $L \tilde{T}_\rho$ has the same distribution as $\tilde{\tau }_L$. Therefore

$$\begin{aligned}&\mathbb {P}\left[ \omega _d \tilde{\tau }_L^{d+1} - d (d+1) \log L - d^2 \log \log L - d^2 \log (d+1) \le \beta \right] \\&= \mathbb {P}\left[ \omega _d \rho \tilde{T}_\rho ^{d+1} - d \log \rho - d^2 \log \log \rho \le \beta \right] , \end{aligned}$$

and by Eq. 6, this gives us Eq. 7. $\square$

We now derive Theorems 2.2 and 2.3 from Theorems 2.9 and 2.10, respectively.

### Proof of Theorem 2.2

We take *Y* to be uniformly distributed on [0, 1]. If the restricted J-M model with intensity $\rho$ runs for time *t*, the intensity of the resulting restricted SPBM is $n=t\rho$, and the distribution of radii is that of *tY*. Let $\beta \in \mathbb {R}$, and define

$$\begin{aligned} t_\rho : = \Big ( \frac{(2 \log \rho + 4 \log \log \rho + \beta ) \vee 0 }{\pi \rho } \Big )^{1/3}, ~~~~~~~~~~~\rho > 1, \end{aligned}$$

so that $\pi \rho t_\rho ^3 - 2 \log \rho - 4 \log \log \rho = \beta ,$ for all large enough $\rho$. Then as $\rho \rightarrow \infty$,

$$\begin{aligned} \log (\rho t_\rho )&= (2/3) \log \rho + (1/3) \log \log \rho + \log ( (2/\pi )^{1/3}) + o(1), \end{aligned}$$

and $\log \log (\rho t_\rho ) = \log \log \rho + \log (2/3) + o(1).$ Therefore since $\mathbb {E}[Y^2]=1/3$, we have

$$\begin{aligned} \pi \rho t_\rho ^3 \mathbb {E}[Y^2]- \log ( \rho t_\rho ) - \log \log (\rho t_\rho ) =&\beta /3 + (2/3) \log \rho + (4/3) \log \log \rho \\&- (2/3) \log \rho - (1/3) \log \log \rho - \log ((2/\pi )^{1/3}) \\&- \log \log \rho - \log (2/3) + o(1) \\ =&(\beta /3) - \log ((16/(27 \pi ))^{1/3}) + o(1), \end{aligned}$$

so applying Theorem 2.9 with $n=\rho t_\rho$, $r_n = t_\rho$, $k=1$ and *Y* uniform on [0, 1] yields

$$\begin{aligned} \mathbb {P}[ \pi \rho T_{\rho }^3 - 2 \log \rho - 4 \log \log \rho \le \beta ] = \mathbb {P}[T_\rho \le t_\rho ] = \mathbb {P}[A \subset Z_{A,1}(n,r_nY)] \\ \rightarrow \exp \Big ( - \Big (\frac{3}{4}\Big ) |A|\Big (\frac{16}{27 \pi }\Big )^{1/3} e^{- \beta /3} - \Big (\frac{3}{4\pi }\Big )^{1/2} |\partial A| \Big (\frac{16}{27 \pi } \Big )^{1/6} e^{-\beta /6} \Big ), \end{aligned}$$

and hence Eq. 8.

For Eq. 9, let $\rho = L^{3}$, so that $\log \log \rho = \log \log L + \log (3)$. Then by Lemma 3.2,

$$\begin{aligned} \mathbb {P}[ \pi \tau _L^3 - 6 \log L -4 \log \log L - \log 81 \le \beta ] = \mathbb {P}[ \pi \rho T_\rho ^3 - 2 \log \rho - 4 \log \log \rho \le \beta ], \end{aligned}$$

and by Eq. 8, this yields Eq. 9.

$$\square $$

### Proof of Theorem 2.3

Let $\beta \in \mathbb {R}$ and for $\rho > 1$ define

22 $$\begin{aligned} t_{\rho } := \Big ( \frac{(2(d-1) \log \rho + 2d(d-1) \log \log \rho + \beta ) \vee 0 }{\omega _d \rho } \Big )^{1/(d+1)}, \end{aligned}$$

so that $\omega _d \rho t_{\rho }^{d+1} - 2(d-1) \log \rho - 2d(d-1) \log \log \rho = \beta ,$ for all large $\rho$. Then as $\rho \rightarrow \infty$,

$$\begin{aligned} \log (\rho t_\rho ) =&\frac{d\log \rho + \log \log \rho }{d+1} + \log \Big ( \Big (\frac{2 (d-1)}{\omega _d} \Big )^{1/(d+1)}\Big ) + o(1), \end{aligned}$$

and $\log \log (\rho t_\rho ) = \log \log \rho + \log (d/(d+1)) + o(1).$ Therefore taking *Y* to be uniformly distributed on [0, 1], so that $\mathbb {E}[Y^d] = 1/(d+1)$, using Eq. 22 we obtain that

$$\begin{aligned} \rho t_\rho \omega _d t_\rho ^d \mathbb {E}[Y^d] - (2-2/d) \log (\rho t_\rho ) - 2(d-2+1/d) \log \log (\rho t_\rho ) \\ = \frac{2(d-1) \log \rho + 2d(d-1) \log \log \rho }{d+1} + \frac{\beta }{d+1} \\ - \frac{(2-2/d)(d\log \rho + \log \log \rho )}{d+1} - (2-2/d)\log \Big (\Big (\frac{2(d-1)}{\omega _d} \Big ) ^{1/(d+1)} \Big ) \\ - 2(d-2+1/d) \log \log \rho - 2(d-2+1/d) \log (d/(d+1)) +o(1) \\ = \frac{2d(d-1) - (2-2/d) -2 (d-2 +1/d) (d+1) }{d+1} \log \log \rho +\frac{\beta }{d+1} - c_d''' +o(1)\\ = \frac{\beta }{d+1} - c_d''' +o(1), \end{aligned}$$

where we set

$$\begin{aligned} c'''_d := \log \Big ( \Big ( \frac{2(d-1)}{\omega _d} \Big )^{(2-2/d)/(d+1)} \Big ( \frac{d}{d+1} \Big )^{2(d-2+1/d)} \Big ). \end{aligned}$$

Therefore applying Theorem 2.10 with $k=1$, $n=\rho t_\rho$ and $r_n = t_\rho$, we obtain that if $d \ge 3$ then

$$\begin{aligned} \mathbb {P}[ \omega _d \rho T_{\rho }^{d+1} - 2 (d-1) \log \rho - 2d (d-1) \log \log \rho \le \beta ] = \mathbb {P}[T_\rho \le t_\rho ] = \mathbb {P}[A \subset Z_{A,1}(n,r_nY)] \\ \rightarrow \exp \Big ( -\Big ( \frac{ c_{d,1} (d+1)^{d-2+1/d} |\partial A| }{d^{d-1}} \Big ) \Big ( \frac{2(d-1)}{\omega _d} \Big )^{(1-1/d)/(d+1)} \\ \times \Big ( \frac{d}{d+1} \Big )^{d-2+1/d} e^{-\beta /(2d+2)} \Big )\\ = \exp \Big ( - \frac{ c_{d-1} \omega _{d-1}^{2d-3} (d-1)^{d-2+(1/d) + ((1-1/d)/(d+1))} |\partial A| e^{-\beta /(2d+2)}}{ \omega _{d-2}^{d-1}\omega _d^{d-2+(1/d) + ((d-1)/(d(d+1)))} 2^{1-(1/d) - (1-1/d)/(d+1)} d^{d-1}} \Big )\\ = \exp \Big ( - \frac{ c_{d-1} \omega _{d-1}^{2d-3} (d-1)^{d(d-1)/(d+1)} |\partial A| e^{-\beta /(2d+2)}}{ \omega _{d-2}^{d-1} \omega _d^{d(d-1)/(d+1)} 2^{(d-1)/(d+1)} d^{d-1} } \Big ), \end{aligned}$$

and hence Eq. 11.

If $d=2$ then our $t_\rho$ defined at Eq. 22 is the same as in the proof of Theorem 2.2. Applying the case $d=2$ of Theorem 2.10, in the same way as we applied Theorem 2.9 in the proof of Theorem 2.2, gives us the same outcome as in Theorem 2.2, namely Eq. 8.

When $d=2$ we obtain Eq. 9 from Eq. 8 as in the proof of Theorem 2.2. When $d \ge 3$, to get Eq. 12 we set $\rho = L^{d+1}$. By Lemma 3.2,

$$\begin{aligned}&\mathbb {P}[\omega _d \tau _L^{d+1} -2 (d^2-1) \log L -2 d (d-1) [\log \log L + \log (d+1)] \le \beta ]&\\&= \mathbb {P}[ \omega _d \rho T_\rho ^{d+1} -2 (d-1) \log \rho -2 d(d-1) \log \log \rho \le \beta ], \end{aligned}$$

and then by Eq. 11, we have Eq. 12.

## Proof of Theorems 2.9 and 2.10

Throughout this section, we assume $d, k \in \mathbb {N}$ are fixed with $d \ge 2$, and $A \subset \mathbb {R}^d$ is compact and nonempty with $A = \overline{A^o}$, and that we are given a nonnegative random variable *Y* satisfying $0< \mathbb {E}[Y^\gamma ] < \infty$ for some $\gamma >d$.

### Preliminaries

We now give some further notation used throughout. Let *o* denote the origin in $\mathbb {R}^d$. Set $\mathbb {H}:= \mathbb {R}^{d-1}\times [0,\infty )$ and $\partial \mathbb {H}:= \mathbb {R}^{d-1}\times \{0\}$.

Given two sets $\mathcal {X},\mathcal{Y}\subset \mathbb {R}^d$, we set $\mathcal {X}\triangle \mathcal{Y}:= (\mathcal {X}\setminus \mathcal{Y}) \cup (\mathcal{Y}\setminus \mathcal {X})$, the symmetric difference between $\mathcal {X}$ and $\mathcal{Y}$. Also, we write $\mathcal {X}\oplus \mathcal{Y}$ for the set $\{x+y: x \in \mathcal {X}, y \in \mathcal{Y}\}$, and $\#(\mathcal {X})$ for the number of elements of $\mathcal {X}$ (possibly $+\infty$). Given also $x \in \mathbb {R}^d$ we write $x+\mathcal{Y}$ for $\{x\} \oplus \mathcal{Y}$.

Given a Borel measure $\mu$ on $\mathbb {R}^d$, and Borel $D \subset \mathbb {R}^d$, let $\mu |_D$ denote the restriction of the measure $\mu$ to *D* (a Borel measure on *D*).

Given $x,y \in \mathbb {R}^d$, we denote by [*x*, *y*] the line segment from *x* to *y*, that is, the convex hull of the set $\{x,y\}$. We write $a \wedge b$ (respectively $a \vee b$) for the minimum (resp. maximum) of any two numbers $a,b \in \mathbb {R}$.

Given $m \in \mathbb {N}$ and functions $f: \mathbb {N}\cap [m,\infty ) \rightarrow \mathbb {R}$ and $g: \mathbb {N}\cap [m,\infty ) \rightarrow (0,\infty )$, we write $f(n) = O(g(n))$ as $n \rightarrow \infty$ if $\limsup _{n \rightarrow \infty }|f(n)|/g(n) < \infty$. We write $f(n)= o(g(n))$ as $n \rightarrow \infty$ if $\lim _{n \rightarrow \infty } f(n)/g(n) =0$, and $f(n) \sim g(n)$ as $n \rightarrow \infty$ if $\lim _{n \rightarrow \infty } f(n)/g(n) =1$. We write $f(n)= \Theta (g(n))$ as $n \rightarrow \infty$ if *f* takes only positive values, and both $f(n)= O(g(n))$ and $g(n)= O(f(n))$. Given $s >0$ and functions $f: (0,s) \rightarrow \mathbb {R}$ and $g:(0,s) \rightarrow (0,\infty )$, we write $f(r) = O(g(r))$ as $r \downarrow 0$ $\limsup _{r \downarrow 0} |f(r) |/g(r) < \infty$, and write $g(r) = \Omega (g(r))$ as $r \downarrow 0$, if $\liminf _{r \downarrow 0} f(r) /g(r) > 0$. We write $f(r)= o(g(r))$ as $r \downarrow 0$ if $\lim _{r \downarrow 0 } f(r)/g(r) =0$, and $f(r) \sim g(r)$ as $r \downarrow 0$ if this limit is 1.

From time to time we shall use the following geometrical lemma.

#### Lemma 4.1

(Geometrical lemma) Suppose $\partial A \in C^2$, and $A = \overline{A^o}$. Given $\varepsilon > 0$, there exists $r_0 = r_0(d,A, \varepsilon )>0$ such that

23 $$\begin{aligned} | B(x,r) \cap A| \ge ((\omega _d/2)-\varepsilon ) r^d, ~~~~ \forall x \in A, r \in (0,r_0). \end{aligned}$$

#### Proof

See Higgs et al. (2025), Lemma 3.4 (or for a proof from first principles, Lemma 3.2(i) of v1 of that paper on Arxiv). $\square$

### Coverage in the boundary by a SPBM in a half-space

In this subsection, we assume $(r_n)_{n >0}$ satisfies Eq. 17 for some $\beta \in \mathbb {R}$. Then as $n \rightarrow \infty$,

24 $$\begin{aligned} r_n = \left( \frac{(2 - 2/d) \log n + 2(d +k-3 + 1/d) \log \log n + \beta +o(1)}{n \omega _d \mathbb {E}[Y^d]} \right) ^{1/d}, \end{aligned}$$

and

25 $$\begin{aligned} \exp (-\omega _d n r_n^d \mathbb {E}[Y^d]) \sim n^{-(2 - 2/d)} (\log n)^{-2(d+k-3+1/d)} e^{-\beta }. \end{aligned}$$

We shall use the following notation throughout the sequel. Given $n >0$ let $\mathcal{U}_n$ be a Poisson point process in $\mathbb {R}^d \times \mathbb {R}_+$ (viewed as a random set of points in $\mathbb {R}^{d+1}$) with intensity measure $n \textrm{Leb}_d \otimes \mu _Y$, where $\textrm{Leb}_d$ denotes *d*-dimensional Lebesgue measure and $\mu _Y$ denotes the distribution of *Y*. Given Borel $D \subset \mathbb {R}^d$, and given any point process $\mathcal {X}$ in $\mathbb {R}^d \times \mathbb {R}_+$, we define

26 $$\begin{aligned} \mathcal{U}_{n,D}&:= \mathcal{U}_{n} \cap (D \times \mathbb {R}_+); \end{aligned}$$

27 $$\begin{aligned} Z_n(\mathcal {X})&: = \{y \in \mathbb {R}^d: \# \{(x,s) \in \mathcal {X}: y \in B(x,r_ns)\} \ge k\}; \end{aligned}$$

28 $$\begin{aligned} Z_n^o(\mathcal {X})&:= (Z_n(\mathcal {X}))^o. \end{aligned}$$

Thus, $\mathcal{U}_{n,D}$ is a Poisson process in $D \times \mathbb {R}_+$ with intensity measure $n \textrm{Leb}_d \otimes \mu _Y$ (strictly speaking, with intensity measure $n \textrm{Leb}_d|_D \otimes \mu _Y$). Equivalently, it can be viewed as a homogeneous, independently *marked* Poisson process in *D* of intensity *n* with marks having the distribution of *Y* (see e.g. Last and Penrose (2017), Theorem 5.6.)

The set $Z_n(\mathcal{U}_{n,D})$ is the region that is covered at least *k* times (for short: the *k*-covered region) for the restricted SPBM in *D* with intensity *n* and with radii having the distribution of $r_n Y$. It is the same as $Z_{D,k}(n,r_nY)$ in earlier notation; we now consider *k* to be fixed and suppress it from the notation. The set $Z_n^o(\mathcal{U}_{n,D})$ is the region covered at least *k* times by a union of *open* balls.

Recall that $\mathbb {H}:= \mathbb {R}^{d-1} \times [0,\infty )$. The main results of this subsection are Lemmas 4.4 and 4.7 below, concerning the limiting probability of covering a $(d-1)$-dimensional region of the form $\Omega \times \{0\}$ in the hyperplane $\partial \mathbb {H}:= \mathbb {R}^{d-1} \times \{0\}$ by $Z_n(\mathcal{U}_{n,\mathbb {H}})$, or of covering the $a_nr_n$-neighbourhood of $\Omega \times \{0\}$ in $\mathbb {H}$, for $a_n$ not growing too fast with *n*. It is crucial for dealing with boundary regions in the proof of Theorem 2.3.

Before getting to that, we present another idea which will be important later. Let ${\zeta }>0$ to be chosen later (think of ${\zeta }$ as a small constant). Given Borel $D \subset \mathbb {R}^d$, we consider two separate, independent Poisson processes $\mathcal{U}'_{n,D}$ and $\mathcal{U}''_{n,D}$, defined by

29 $$\begin{aligned} \mathcal{U}'_{n,D} = \mathcal{U}_n \cap (D \times [0,n^{\zeta }]); ~~~~~~~~~ \mathcal{U}''_{n,D} = \mathcal{U}_n \cap (D \times (n^{\zeta },\infty )). \end{aligned}$$

For various sets *D* and ultimately for $D=A$, we shall be interested in coverage of certain regions by the set $Z_n(\mathcal{U}_{n,D})$; the next lemma says that the limiting probability of coverage is unaffected if we take $Z_{n}(\mathcal{U}'_{n,D})$ instead of $Z_{n}(\mathcal{U}_{n,D})$; this is useful because the random sets $Z_{n}(\mathcal{U}'_{n,D})$ have better spatial independence properties, considered as a function of *D*.

#### Lemma 4.2

(Asymptotic equivalence of coverage events using $\mathcal{U}_{n,D}$ or $\mathcal{U}'_{n,D}$) Let $n_0 \in (0,\infty )$. Suppose that Borel $D_n \subset \mathbb {R}^d$ and $E_n \subset \mathbb {R}^d$ are defined for each $n \ge n_0$. Then as $n \rightarrow \infty$,

30 $$\begin{aligned} \mathbb {P}[\{E_n \subset Z_n(\mathcal{U}_{n,D_n} )\} \setminus \{E_n \subset Z_n(\mathcal{U}'_{n,D_n})\}] \rightarrow 0. \end{aligned}$$

#### Proof

Recall that we are assuming $\mathbb {E}[Y^\gamma ] < \infty$ for some $\gamma >d$. Given $x \in \mathbb {R}^d$, let $N_n(x)$ denote the number of points $(y,t) \in \mathcal{U}''_{n,D_n}$ such that $\Vert y-x\Vert \le r_n t$. Then by the Hölder and Markov inequalities,

$$\begin{aligned} \mathbb {P}[N_n(x) \ge 1 ] \le \mathbb {E}[N_n(x)]&\le n \int _{(n^{\zeta },\infty )} |B(x,r_nt)| \mu _Y(dt) \\&= n \omega _d r_n^d \mathbb {E}[Y^d \textbf{1}_{\{Y> n^{\zeta }\}} ] \\&\le n \omega _d r_n^d (\mathbb {E}[Y^{\gamma }])^{d/\gamma } (\mathbb {P}[Y > n^{\zeta }])^{(\gamma -d)/\gamma } \\&\le \omega _d (\mathbb {E}[Y^{\gamma }])^{d/\gamma } nr_n^d (\mathbb {E}[Y^{\gamma }]/n^{{\zeta }\gamma } )^{(\gamma - d)/\gamma }, \end{aligned}$$

which tends to zero since $nr_n^d = O(\log n)$ by Eq. 24. Therefore

31 $$\begin{aligned} \sup _{x \in \mathbb {R}^d} \mathbb {P}[x \in Z_n(\mathcal{U}''_{n,D_n} ) ] \rightarrow 0 ~~ \textrm{as} ~ n \rightarrow \infty . \end{aligned}$$

Let $n \ge n_0$. Since $E_n \subset \mathbb {R}^d$, $E_n$ is separable; see e.g. Pryce (1973), page 20. Let $\{x_{n,i}\}_{i \in \mathbb {N}}$ be an enumeration of a countable dense set in $E_n$. Define the ($\mathbb {N}\cup +\infty$)-valued random variable $J_n$ to be the first *i* such that $x_{n,i} \notin Z_n(\mathcal{U}'_{n,D_n})$, or $J_n:= + \infty$ if there is no such *i*. Since $E_n \setminus Z_n(\mathcal{U}'_{n,D_n})$ is open in $E_n$, if $E_n \setminus Z_n(\mathcal{U}'_{n,D_n}) \ne \emptyset$ then $J_n < \infty$. Therefore

$$\begin{aligned} \mathbb {P}[ E_n \subset Z_n(\mathcal{U}_{n,D_n}) |\{E_n \subset Z_n(\mathcal{U}'_{n,D_n}) \}^c] \le \mathbb {P}[x_{n,J_n} \in Z_n(\mathcal{U}''_{n,D_n})|J_n < \infty ] , \end{aligned}$$

which tends to zero by Eq. 31 and the independence of $\mathcal{U}'_{n,D_n}$ and $\mathcal{U}''_{n,D_n}$, and Eq. 30 follows. $\square$

The following terminology and notation will be used repeatedly in the sequel. Given $x \in \mathbb {R}^d$, we let $\pi _1(x),\ldots ,\pi _d(x)$ denote the co-ordinates of *x*, and refer to $\pi _d(x)$ as the *height* of *x*. Given $\textbf{x}_1 = (x_1,s_1), \ldots , \textbf{x}_d = (x_d,s_d) \in \mathbb {R}^d \times \mathbb {R}_+$, if $\cap _{i=1}^d \partial B(x_i,s_i)$ consists of exactly two points, we refer to these as $p(\textbf{x}_1,\ldots ,\textbf{x}_d)$ and $q(\textbf{x}_1,\ldots ,\textbf{x}_d)$ with $p(\textbf{x}_1,\ldots ,\textbf{x}_d)$ at a smaller height than $q(\textbf{x}_1,\ldots ,\textbf{x}_d)$ (or if they are at the same height, take $p(\textbf{x}_1,\ldots ,\textbf{x}_d) < q(\textbf{x}_1,\ldots ,\textbf{x}_d)$ in the lexicographic ordering). Define the indicator function

32 $$\begin{aligned} h(\textbf{x}_1,\ldots ,\textbf{x}_d) :=&\textbf{1} \{ \pi _d(x_1) \le \min (\pi _d(x_2), \ldots , \pi _d(x_d)) \} \nonumber \\&\times \textbf{1} \{ \#( \cap _{i=1}^d \partial B(x_i,r_i) ) = 2 \} \textbf{1} \{ \pi _d(x_1) < \pi _d(q(\textbf{x}_1,\ldots ,\textbf{x}_d))\} . \end{aligned}$$

Given also $n >0$ we define

33 $$\begin{aligned} h_n(\textbf{x}_1,\ldots ,\textbf{x}_d):=h((x_1,r_ns_1),\ldots ,(x_d,r_ns_d)); \end{aligned}$$

34 $$\begin{aligned} p_n(\textbf{x}_1,\ldots ,\textbf{x}_d):=p((x_1,r_ns_1),\ldots ,(x_d,r_ns_d)); \end{aligned}$$

35 $$\begin{aligned} q_n(\textbf{x}_1,\ldots ,\textbf{x}_d):=q((x_1,r_ns_1),\ldots ,(x_d,r_ns_d)). \end{aligned}$$

**Fig. 3:**
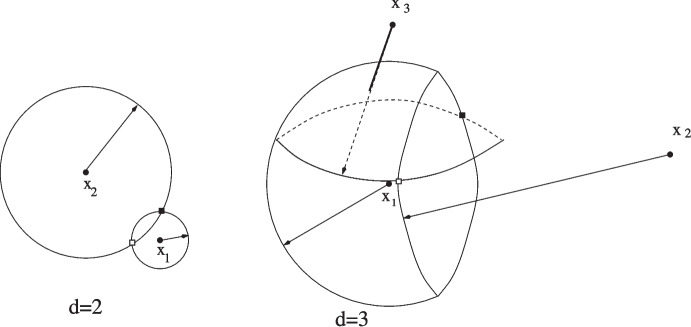
Examples in $d=2$ and $d=3$ showing a *d*-tuple $\{\textbf{x}_1,\ldots ,\textbf{x}_d\}$, satisfying $h_n(\textbf{x}_1,\ldots ,\textbf{x}_d)=1$ and showing $p_n(\{\textbf{x}_1,\ldots ,\textbf{x}_d\})$ (white square) and $q_n(\{\textbf{x}_1,\ldots ,\textbf{x}_d\})$ (black square), where, for $i= 1,\ldots ,d$, $\textbf{x}_i= (x_i,s_i)$ and the arrow from $x_i$ is of length $r_n s_i.$

These notations are illustrated in Fig. 3.

#### Lemma 4.3

(Integrability of *h*) There exists a constant *c* depending only on *d* such that for all $x_1 \in \mathbb {R}^d$ and all $s_1,\ldots ,s_d \in \mathbb {R}_+$,

36 $$\begin{aligned} \int _{\mathbb {R}^d} \cdots \int _{\mathbb {R}^d} h((x_1,s_1),\ldots ,(x_d,s_d)) dx_2 \cdots dx_d \le c\prod _{i=1}^d (1 \vee s_i^{d-1}). \end{aligned}$$

#### Proof

Denote the left hand side of Eq. 36 by $I(x_1,s_1,\ldots ,s_d)$. Divide $\mathbb {R}^d$ into rectilinear unit hypercubes $Q_z, z \in \mathbb {Z}^d$, with $Q_z$ centred at *z* for each *z*. Then

$$\begin{aligned} I(x_1,s_1,\ldots ,s_d)&\le \sum _{z \in \mathbb {Z}^d} \int _{\mathbb {R}^d} \cdots \int _{\mathbb {R}^d} \textbf{1}\{ \cap _{i=1}^d \partial B(x_i,s_i) \cap Q_z \ne \emptyset \} dx_2 \cdots dx_d \\&\le \sum _{z \in \mathbb {Z}^d} \int _{\mathbb {R}^d} \cdots \int _{\mathbb {R}^d} \prod _{i=1}^d \textbf{1}\{ \partial B(x_i,s_i) \cap Q_z \ne \emptyset \} dx_2 \cdots dx_d \\&= \sum _{z \in \mathbb {Z}^d} \textbf{1}\{ \partial B(x_1,s_1) \cap Q_z \ne \emptyset \} \times \prod _{i=2}^d \int _{\mathbb {R}^d} \textbf{1}\{ \partial B(x_i,s_i) \cap Q_z \ne \emptyset \} dx_i. \end{aligned}$$

Given $s \ge 0$, the value of the integral $\int _{\mathbb {R}^d} \textbf{1}\{ \partial B(x,s) \cap Q_z \ne \emptyset \} dx$ does not depend on *z*, and is bounded by $c' (1 \vee s^{d-1})$ for some constant $c'$, independent of *s*. Moreover the sum $\sum _{z \in \mathbb {Z}^d} \textbf{1}\{ \partial B(x,s) \cap Q_z \ne \emptyset \}$ is bounded by $c'' (1 \vee s^{d-1})$ for some further constant $c''$, independent of *s* and *x*. Applying these two observations gives us Eq. 36. $\square$

We are now ready to present the first main result of this section. In this result, $|\Omega |$ denotes the $(d-1)$-dimensional Lebesgue measure of $\Omega$. Recall the definition of $c_{d,1}$ at Eq. 15.

#### Lemma 4.4

(Coverage of a portion of the boundary of a half-space) Let $\Omega \subset \mathbb {R}^{d-1}$ be closed, bounded and Riemann measurable. Assume that Eq. 17 holds for some $\beta \in \mathbb {R}$ or $\beta = + \infty$, and also that $\limsup _{n \rightarrow \infty } ( n r_n^d/(\log n )) < \infty$. Then

37 $$\begin{aligned} \lim _{n \rightarrow \infty } (\mathbb {P}[ \Omega \times \{0\} \subset Z_n(\mathcal{U}_{n,\mathbb {H}})] ) = \exp \left( - c_{d,k} \Big ( \frac{(\mathbb {E}[Y^{d-1}])^{d-1}}{(\mathbb {E}[Y^{d}])^{d-2+ 1/d}} \Big ) |\Omega | e^{- \beta /2} \right) . \end{aligned}$$

#### Remark 4.5

When $\beta < \infty$, the extra condition $\limsup _{n \rightarrow \infty } ( n r_n^{d}/(\log n )) < \infty$ is automatic. When $\beta =\infty$, in Eq. 37 we use the convention $e^{-\infty }:= 0$.

#### Proof of Lemma 4.4

Assume for now that $\beta < \infty$. Considering the slices of balls induced by the points of $\mathcal{U}_{n,\mathbb {H}}$ that intersect the hyperplane $\mathbb {R}^{d-1} \times \{0\}$, we have a $(d-1)$-dimensional SPBM, where we claim the parameters (in the notation of Lemma 3.1) are

38 $$\begin{aligned} \delta = r_n, ~~~ \lambda = n r_n \mathbb {E}[Y], ~~~~ \alpha = \omega _{d} \mathbb {E}[Y^d]/(2 \mathbb {E}[Y]). \end{aligned}$$

We justify these claims as follows. The intensity of balls intersecting the hyperplane $\partial \mathbb {H}_d$ per unit ‘area’ (i.e. per unit $(d-1)$-dimensional Lebesgue measure), is equal to $n \int _0^\infty \mathbb {P}[r_n Y \ge t]dt = n r_n \mathbb {E}[Y]$. Also if *W* denotes the radius of a slice, divided by $r_n$, the distribution of *W* is that of $\tilde{Y}(1-U^2)^{1/2}$, where $\tilde{Y}$ has the size-biased distribution of *Y* (i.e., $\mathbb {P}[\tilde{Y} \in dt] = t \mathbb {P}[Y \in dt]/\mathbb {E}[Y]$), and *U* is uniformly distributed on [0, 1], independent of $\tilde{Y}$. Therefore

39 $$\begin{aligned} \mathbb {E}[W^{d-1}]&= \mathbb {E}[\tilde{Y}^{d-1}] \int _0^1 (1 -u^2)^{(d-1)/2} du \nonumber \\&= (\mathbb {E}[Y^d]/\mathbb {E}[Y]) \omega _d/(2\omega _{d-1}), \end{aligned}$$

and the asserted value of $\alpha$ follows.

By Eq. 38, followed by Eq. 17,

$$\begin{aligned} \alpha \delta ^{d-1} \lambda&= \Big ( \frac{ \omega _d \mathbb {E}[Y^d]}{2 \mathbb {E}[Y]} \Big ) r_n^{d-1} n r_n \mathbb {E}[Y] = (\omega _d/2) n r_n^d \mathbb {E}[Y^d] \\&= (1-1/d) \log n + (d+k-3 + 1/d) \log \log n + (\beta /2) + o(1). \end{aligned}$$

Also by Eqs. 38 and 24,

$$\begin{aligned} \log \lambda&= (1-1/d) \log n + \Big (\frac{1}{d}\Big ) \log \log n + \log \Big (\Big (\frac{2-2/d}{\omega _d \mathbb {E}[Y^d]}\Big )^{1/d} \mathbb {E}[Y] \Big ) + o(1). \end{aligned}$$

Also $$ \log \log \lambda = \log \log n + \log (1-1/d) + o(1). $$ Hence

$$\begin{aligned} \alpha \delta ^{d-1} \lambda - \log \lambda - (d+k-3) \log \log \lambda = (\beta /2) - \log (c''_{d,k,Y}) + o(1), \end{aligned}$$

where we take

$$\begin{aligned} c''_{d,k,Y}&: = \left( \frac{2-2/d}{\omega _d \mathbb {E}[Y^d] }\right) ^{1/d} \mathbb {E}[Y] (1-1/d)^{d+k-3} =(1-1/d)^{d+k-3+1/d} \left( \frac{2 (\mathbb {E}[Y])^d }{\omega _d \mathbb {E}[Y^d]} \right) ^{1/d}. \end{aligned}$$

By Eq. 39, and a similar calculation for $\mathbb {E}[W^{d-2}]$,

$$\begin{aligned} \frac{(\mathbb {E}[W^{d-2}])^{d-1}}{( \mathbb {E}[W^{d-1}])^{d-2}} = \left( \frac{\omega _{d-1} \mathbb {E}[Y^{d-1}]}{2 \omega _{d-2} \mathbb {E}[Y]} \right) ^{d-1} \left( \frac{\omega _d \mathbb {E}[Y^d]}{2 \omega _{d-1} \mathbb {E}[Y]} \right) ^{2-d} = \frac{ \omega _{d-1}^{2d-3} (\mathbb {E}[Y^{d-1}])^{d-1}}{2 \omega _{d-2}^{d-1} \omega _d^{d-2} (\mathbb {E}[Y^d])^{d-2} \mathbb {E}[Y]}. \end{aligned}$$

Thus by Lemma 3.1 we obtain that

$$\begin{aligned} \lim _{n \rightarrow \infty } (\mathbb {P}[ \Omega \times \{0\} \subset Z_n(\mathcal{U}_{n,\mathbb {H}})]) = \exp \Big ( - \Big (\frac{c_{d-1} \omega _{d-1}^{2d-3} (\mathbb {E}[Y^{d-1}])^{d-1} }{ (k-1)! 2 \omega _{d-2}^{d-1} \omega _d^{d-2} \mathbb {E}[Y] (\mathbb {E}[Y^d])^{d-2} }\Big ) \\ \times c''_{d,k,Y} |\Omega | e^{-\beta /2} \Big ), \end{aligned}$$

and hence Eq. 37.

Having now verified (37) in the case where $\beta < \infty$, we can then easily deduce (37) in the other case too. $\square$

Next we aim to show that for any bounded Borel set $\Omega$ in $\mathbb {R}^{d-1}$, the probability of there being any uncovered region in $\mathbb {H}$, lying close to the boundary region $\Omega \times \{0\}$ but not intersecting $\partial \mathbb {H}$ itself, is vanishingly small. We shall do this by using the fact that such a region must have an “exposed lower corner” in $\mathbb {H}$ near $\Omega \times \{0\}$, and estimating the number of such corners.

**Fig. 4:**
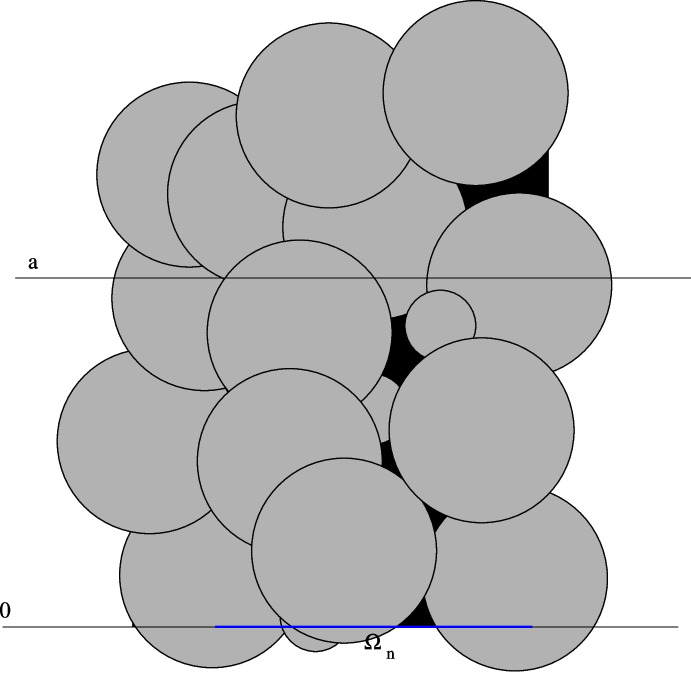
Example in $d=2$ with $k=1$. Here $M_n(\Omega ,a) = 11$ since, taking the uncovered regions below height *a* in descending order, we have a region with 5 corners that all contribute to $M_n(\Omega ,a)$, one with 4 corners three of which contribute, and one with 3 corners that all contribute, and a region with one interior corner that does not contribute. The thick blue line within the *x*-axis is the region $\Omega _n$

For this argument we need further notation. Given $\Omega \subset \mathbb {R}^{d-1}$, $a >0$, and given $(r_n)_{n >0}$, let $M_n(\Omega ,a)$ denote the number of *d*-tuples of marked points $\textbf{x}_1 = (x_1,s_1),\ldots , \textbf{x}_d = (x_d,s_d) \in \mathcal{U}_{n,\mathbb {H}}$, such that $h_n(\textbf{x}_1,\ldots ,\textbf{x}_d)=1$, and moreover $q_n(\textbf{x}_1,\ldots ,\textbf{x}_d) \in ( \Omega \times (0,a r_n]) \setminus Z_n(\mathcal{U}_{n,\mathbb {H}} \setminus \{\textbf{x}_1,\ldots ,\textbf{x}_d\})$. Thus if $k=1$ then $M_n(\Omega ,a)$ is the number of corners of an uncovered region that lie in $\Omega \times (0,a r_n]$ for which at least one of the balls having that corner on its boundary has its centre below the corner, as illustrated in Fig. 4.

#### Lemma 4.6

(Estimating the mean number of exposed lower corners near $\partial \mathbb {H}$) Let $\Omega$ be a bounded Borel set in $\mathbb {R}^{d-1}$, let $a \in [1,\infty )$ and let $(r_n)_{n >0}$ satisfy Eq. 17 for some $\beta \in \mathbb {R}$ or $\beta = + \infty$, and that $\limsup _{n \rightarrow \infty } ( n r_n^d/(\log n )) < \infty$. Let $a \in [1,\infty ).$ Then

40 $$\begin{aligned} \lim _{n \rightarrow \infty } (\mathbb {E}[M_n(\Omega ,a)]) = 0. \end{aligned}$$

#### Proof

Choose $\delta \in (0,1)$ such that $\mathbb {P}[ Y> \delta ] > \delta$. Then we can and do choose $c' >0$ so that

$$ \omega _{d-1} \delta ^d 2^{1-d} ((\delta /2) \wedge u) \ge c'u, ~~~ \forall u \in [0,a]. $$

To be definite, take $c':= a^{-1} \omega _{d-1} \delta ^{d} 2^{-d}$; then the displayed inequality holds for $u=a$, and hence also for smaller *u*.

**Fig. 5:**
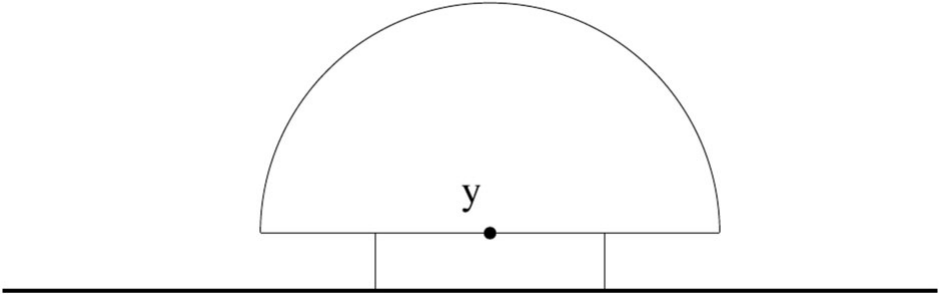
Illustration of the geometric estimate used to derive Eq. 41. The thick line at the bottom is $\partial \mathbb {H}$

For any $y \in \mathbb {H}$, let $N_n(y)$ denote the number of balls making up $Z_n(\mathcal{U}_{n,\mathbb {H}})$ which cover *y*, i.e. the number of $(x,s) \in \mathcal{U}_{n,\mathbb {H}}$ such that $\Vert x-y\Vert \le r_n s$. Also, let $\mathbb {H}_y:= \{z \in \mathbb {H}:\pi _d(z) > \pi _d(y) \}$. Then for all *n* and all $y \in \mathbb {H}$ with $0 \le \pi _d(y) \le ar_n$, for all $s >0$, the half-ball $B(y,r_ns) \cap \mathbb {H}_y$, and also the cylinder with radius $sr_n/2$, upper face centred on *y* and height $(r_ns/2) \wedge \pi _d(y)$, are disjoint and contained in $B(y,r_ns) \cap \mathbb {H}$, as illustrated in Fig. 5. Therefore

41 $$\begin{aligned} \mathbb {E}[N_n(y)]&= n \int _{\mathbb {R}_+} |B(y,r_ns) \cap \mathbb {H}| \mu _Y(ds) \nonumber \\&\ge n \int _{\mathbb {R}_+} (\omega _d r_n^d s^d/2)\mu _Y(ds) + n \int _{(\delta ,\infty )} \omega _{d-1} (r_ns/2)^{d-1} ((r_ns/2) \wedge \pi _d(y)) \mu _Y(ds) \nonumber \\&\ge (n \omega _d/2) r_n^d \mathbb {E}[Y^d] + n\delta \omega _{d-1} (\delta r_n/2)^{d-1} r_n ((\delta /2) \wedge (\pi _d(y)/r_n)) \nonumber \\&\ge (n \omega _d/2) r_n^d \mathbb {E}[Y^d] + c' n r_n^{d-1} \pi _d(y). \end{aligned}$$

By the Mecke formula (see e.g. Last and Penrose (2017)), taking $Y_1,\ldots ,Y_d$ to be independent random variables with the distribution of *Y*, there is a constant *c* such that

42 $$\begin{aligned} \mathbb {E}[M_n(\Omega ,a)] \le&c n^d \int _{\mathbb {H}} \cdots \int _{\mathbb {H}} \mathbb {E}[h_n((x_1,Y_1),\ldots ,(x_d,Y_d)) \nonumber \\&\times \textbf{1}\{ q_n((x_1,Y_1),\ldots ,(x_d,Y_d)) \in \Omega \times [0,ar_n] \} (k (n\omega _d r_n^d)^{k-1}) \nonumber \\&\times e^{- n (\omega _d/2) \mathbb {E}[Y^d] r_n^{d} - c' n r_n^{d-1} \pi _d( q_n((x_1,Y_1),\ldots ,(x_d,Y_d)) )}] dx_d \cdots dx_1 . ~~~ \end{aligned}$$

Now we change variables to $y_i= r_n^{-1}(x_i-x_1)$ for $2 \le i \le d$, noting that

$$\begin{aligned} \pi _{d}(q_n((x_1,Y_1),\ldots ,(x_d,Y_d))) = \pi _d(x_1) + \pi _d( q_n((o,Y_1),(x_2 -x_1,Y_2), \ldots , (x_d-x_1,Y_d)) ). \end{aligned}$$

With these changes of variable, we get a factor of $r_n^{d(d-1)}$ on changing from $(x_2,\ldots ,x_d)$ to $(y_2,\ldots ,y_d)$. Hence by Eq. 25, there is a constant $c''$ such that the expression on the right hand side of Eq. 42 is at most

43 $$\begin{aligned}&c'' n^{d+k-1} r_n^{d(d+k-2)} n^{- (1-1/d)} (\log n)^{-(d+k-3+1/d)} \int _0^{a r_n} e^{-c' n r_n^{d-1} u} du \nonumber \\&\times \int _{\mathbb {H}} \cdots \int _{\mathbb {H}} \mathbb {E}[h_n((o,Y_1),(r_ny_2,Y_2),\ldots ,(r_ny_d,Y_d)) \nonumber \\ &~~~~~~~~~~~~~~~~~ \times e^{ - c' n r_n^{d-1} \pi _d( q_n((o,Y_1),(r_ny_2,Y_2), \ldots ,(r_ny_d,Y_d))) } ] dy_d \ldots dy_2. \end{aligned}$$

In the last expression the first line is bounded by a constant times the expression

$$\begin{aligned} n^{d+k-3+1/d} (r_n^d)^{d+k-2 - (d-1)/d} (\log n )^{-(d+k-3 +1/d)} = (n r_n^{d}/\log n)^{d+k-3+1/d}, \end{aligned}$$

which is bounded because $n r_n^{d} = O(\log n)$ by Eq. 24. Using Fubini’s theorem and the definitions of $h_n(\cdot )$ and $q_n(\cdot )$ in Eqs. 33 and 35, the expression in the second and third lines of Eq. 43 can be rewritten as

$$\begin{aligned} \mathbb {E}\Big [ \int _{\mathbb {H}} \cdots \int _{\mathbb {H}} h((o,Y_1),(y_2,Y_2),\ldots ,(y_d,Y_d)) e^{ - c' n r_n^{d} \pi _d( q((o,Y_1),(y_2,Y_2), \ldots ,(y_d,Y_d))) } dy_d \ldots dy_2 \Big ] \\ = \int _{\mathbb {R}_+^d} \int _{\mathbb {H}^{d-1}} h((o,u_1),(y_2,u_2),\ldots ,(y_d,u_d)) e^{ - c' n r_n^{d} \pi _d( q((o,u_1),(y_2,u_2), \ldots ,(y_d,u_d)))} \\ d(y_2,\ldots ,y_d) \mu _Y^d(d(u_1,\ldots ,u_d)). \end{aligned}$$

This tends to zero by dominated convergence because $n r_n^{d} \rightarrow \infty$ by Eq. 17 and because the indicator function $(\textbf{u}, \textbf{y}) \mapsto h((o,u_1),(y_2,u_2),\ldots , (y_d,u_d))$ is integrable and is zero when $\pi _d(q((o,u_1),(y_2,u_2),\ldots ,(y_d,u_d))) \le 0$; the integrability follows from Lemma 4.3 and the fact that $\mathbb {E}[Y^{d-1}] < \infty$ since we assume $\mathbb {E}[Y^{\gamma }]<\infty$ for some $\gamma > d$.

Therefore the expression in Eq. 43 tends to zero as $n \rightarrow \infty$, and Eq. 40 follows. $\square$

We are now ready to state the second main result of this subsection.

#### Lemma 4.7

(Coverage of a region just inside the boundary of a half-space) For each $n >0$ let $\Omega _n \subset \mathbb {R}^{d-1}$ be Riemann measurable, with $\cup _{n>0} \Omega _n$ bounded. Assume that Eq. 17 holds for some $\beta \in \mathbb {R}$ or $\beta = + \infty$, and that $\limsup _{n \rightarrow \infty } ( n r_n^d/(\log n )) < \infty$. Also let $a_n \in (0,\infty )$ for each $n >0$, and assume for some $\varepsilon >0$ that $a_n = O(n^{(1/d) - \varepsilon })$ as $n \rightarrow \infty$. Then

44 $$\begin{aligned} \lim _{n \rightarrow \infty } (\mathbb {P}[\{(\Omega _n \times \{0\} ) \cup (&(\partial \Omega _n) \times [0,a_n r_n]) \subset Z^o_n(\mathcal{U}'_{n,\mathbb {H}}) \} \nonumber \\&\setminus \{ ( \Omega _n \times [0, a_n r_n] ) \subset Z_n( \mathcal{U}'_{n,\mathbb {H}}) \}] ) = 0. \end{aligned}$$

#### Proof

For the duration of this proof only, let $F_n$ be the event displayed in Eq. 44 but with $\mathcal{U}'_{n,\mathbb {H}}$ changed to $\mathcal{U}_{n,\mathbb {H}}$ both times, i.e.

$$\begin{aligned} F_n:= \{(\Omega _n \times \{0\} ) \cup ( (\partial \Omega _n) \times [0,a_n r_n]) \subset Z^o_n(\mathcal{U}_{n,\mathbb {H}}) \} \setminus \{ ( \Omega _n \times [0, a_n r_n] ) \subset Z_n( \mathcal{U}_{n,\mathbb {H}}) \}. \end{aligned}$$

By Lemma 4.2, to prove Eq. 44 it suffices to prove that $\mathbb {P}[F_n ] \rightarrow 0$ as $n \rightarrow \infty$.

Let $$E_n$$ be the (exceptional) event that there exist *d* distinct marked points $$(x_1,s_1),\ldots ,$$ $$ (x_d,s_d)$$ of $$\mathcal{U}_{n,\mathbb {H}}$$ such that $$ \cap _{i=1}^d \partial B(x_i, r_n s_i) $$ has non-empty intersection with the hyperplane $$\partial \mathbb {H}$$. Then $$\mathbb {P}[E_n]=0$$.

Suppose that $F_n \setminus E_n$ occurs. Let *w* be a location of minimal height (i.e., *d*-coordinate) in the closure of $( \Omega _n \times [0,a_n r_n]) \setminus Z_n(\mathcal{U}_{n,\mathbb {H}})$. Since we assume $(\partial \Omega _n) \times [0,a_n r_n] \subset Z^o_n( \mathcal{U}_{n,\mathbb {H}})$ occurs, *w* must lie in $\Omega _n^o \times (0,a_nr_n]$. Also we claim that *w* must be a ‘corner’ given by the meeting point of the boundaries of exactly *d* balls $B(x_1,r_ns_1), \ldots , B(x_d,r_ns_d)$ with $\{(x_i,s_i)\}_{i=1}^d \subset \mathcal{U}_{n,\mathbb {H}}$, and with $x_1$ the lowest of the *d* points $x_1,\ldots ,x_d$, and with $\#(\cap _{i=1}^d \partial B(x_i,r_ns_i) ) =2$, and $w \notin Z_n( \mathcal{U}_{n,\mathbb {H}} \setminus \{(x_1,s_1),\ldots ,(x_d,s_d)\})$.

Indeed, if *w* is not at the boundary of any such ball, then for some $\delta >0$ we have $B(w, \delta ) \cap Z_n(\mathcal{U}_{n,\mathbb {H}}) = \emptyset$, and then we could find a location in $(\Omega _n \times [0,ar_n]) \setminus Z_n(\mathcal{U}_{n,\mathbb {H}})$ lower than *w*, a contradiction. Next, suppose instead that *w* lies at the boundary of fewer than *d* such balls. Then denoting by *L* the intersection of the supporting hyperplanes at *w* of each of these balls, we have that *L* is an affine subspace of $\mathbb {R}^d$, of dimension at least 1. Take $\delta >0$ small enough so that $B(w,\delta )$ does not intersect any of the boundaries of balls centred at points of $\mathcal{U}_{n,\mathbb {H}}$ (viewed here as a marked Poisson point process in $\mathbb {R}^d$) and with radius given by their marks times $r_n$, other than those which meet at *w*. Taking $w' \in L \cap B(w, \delta ) \setminus \{w\}$ such that $w'$ is at least as low as *w*, we have that $w'$ lies in the interior of $Z_n(\mathcal{U}_{n,\mathbb {H}})^c$. Hence for some $\delta ' >0$, $B(w',\delta ') \cap Z_n(\mathcal{U}_{n,\mathbb {H}}) = \emptyset$ and we can find a location in $B(w',\delta ')$ that is lower than *w*, yielding a contradiction for this case too. Finally, with probability 1 there is no set of more than *d* points of $\mathcal{U}_{n,\mathbb {H}}$ such that the boundaries of the associated balls have non-empty intersection, so *w* is not at the boundary of more than *d* such balls. Thus we have justified the claim.

Moreover *w* must be the point $q_n(\textbf{x}_1,\ldots ,\textbf{x}_d)$ rather than $p_n(\textbf{x}_1,\ldots ,\textbf{x}_d)$, where for $1 \le i \le d$ we write $\textbf{x}_i$ for $(x_i,r_i)$, because otherwise by extending the line segment from $q_n(\textbf{x}_1,\ldots ,\textbf{x}_d)$ to $p_n(\textbf{x}_1,\ldots ,\textbf{x}_d)$ slightly beyond $p_n(\textbf{x}_1,\ldots ,\textbf{x}_d)$ we could find a point in $(\Omega _n \times [0,a r_n]) \setminus Z_n(\mathcal{U}_{n,\mathbb {H}})$ lower than *w*, contradicting the statement that *w* is a location of minimal height in the closure of $(\Omega _n \times [0,a r_n]) \setminus Z_n(\mathcal{U}_{n,\mathbb {H}})$. Moreover, *w* must be strictly higher than $x_1$, since if $\pi _d(w) \le \min (\pi _d(x_1), \ldots ,\pi _d(x_d))$, then locations just below *w* would lie in $(\Omega _n \times [0,a r_n]) \setminus Z_n(\mathcal{U}_{n,\mathbb {H}})$, contradicting the statement that *w* is a point of minimal height in the closure of $(\Omega _n \times [0,ar_n]) \setminus Z_n(\mathcal{U}_{n,\mathbb {H}})$. Hence, $h_n(\textbf{x}_1,\ldots ,\textbf{x}_d) = 1$, where $h_n(\cdot )$ was defined at Eq. 33.

Thus if $F_n \setminus E_n$ occurs, then $M_n(\Omega ,a_n) \ge 1$, where we take $\Omega := \cup _{n >1} \Omega _n$, and $M_n(\Omega ,a)$ was defined just before Lemma 4.6. Hence by Markov’s inequality, it suffices to show that $\mathbb {E}[M_n(\Omega ,a_n)] \rightarrow 0$ as $n\rightarrow \infty$.

Choose $a \in [1,\infty )$ such that

$$ \int _{(a,\infty )} x^d \mu _Y(dx) < \varepsilon \mathbb {E}[Y^d]/4. $$

Since $\mathbb {E}[M_n(\Omega ,a')]$ is monotone nondecreasing in $a'$, we may assume without loss of generality that $a_n > a$. For $y \in \mathbb {H}$ with $a r_n \le \pi _d(y) \le a_n r_n$, instead of Eq. 41 we use the estimate

$$\begin{aligned} \mathbb {E}[N_n(y)]&= n \int _{\mathbb {R}_+} |B(y,r_ns) \cap \mathbb {H}| \mu _Y(ds) \nonumber \\&\ge n \int _{(0,a)} (\omega _d r_n^d s^d)\mu _Y(ds) \nonumber \\&\ge n \omega _d r_n^d \mathbb {E}[Y^d] (1- \varepsilon /4 ). \nonumber \end{aligned}$$

By this, and the Mecke formula, there is a new constant *c* such that

45 $$\begin{aligned} \mathbb {E}[M_n(\Omega ,a_n) -M_n(\Omega ,a)] \le c n^d (nr_n^d)^{k-1} \int _{\mathbb {H}} \cdots \int _{\mathbb {H}} \mathbb {E}[h_n((x_1,Y_1),\ldots ,(x_d,Y_d)) \nonumber \\ \times \textbf{1}\{ q_n((x_1,Y_1),\ldots ,(x_d,Y_d)) \in \Omega \times [ar_n,a_nr_n] \} e^{- n \omega _d r_n^d \mathbb {E}[Y^d] (1- \varepsilon /4) }] dx_d \cdots dx_1 . ~~~ \end{aligned}$$

Changing variables as before to $y_i= r_n^{-1}(x_i-x_1)$ for $2 \le i \le d$, and using Eq. 25, we find there is a constant $c'''$ such that the expression in the right hand side of Eq. 45 is at most

$$\begin{aligned} c''' n^{d+k-1} r_n^{d(d+k-2)} n^{- (2-2/d) + \varepsilon /2} \mathbb {E}\Big [ \int _{\mathbb {H}} \cdots \int _{\mathbb {H}} h_n((o,Y_1),(r_ny_2,Y_2),\ldots ,(r_ny_d,Y_d)) \nonumber \\ \times \textbf{1}_{\Omega \times [ar_n,a_nr_n]} ( x_1 + q_n((o,Y_1),(r_ny_2,Y_2), \ldots ,(r_ny_d,Y_d))) dx_1 dy_d \ldots dy_2 \Big ] \nonumber \\ \le c''' (nr_n^d)^{d+k-2} n^{-1+(2/d)+ \varepsilon /2} |\Omega |(a_n-a) r_n \nonumber \\ \times \mathbb {E}\Big [ \int _{\mathbb {H}^{d-1}} h((o,Y_1),(y_2,Y_2), \ldots ,(y_d,Y_d)) d ((y_2,\ldots ,y_d)) \Big ]. \nonumber \end{aligned}$$

Since $\mathbb {E}[Y^{d-1}]<\infty$ the expectation in the last line is finite by Lemma 4.3, and since also $a_n =O(n^{(1/d)-\varepsilon })$ we obtain that

$$ \mathbb {E}[M_n(\Omega ,a_n) - M_n(\Omega ,a)] = O( (nr_n^d)^{d+k-2+ 1/d} n^{-1+(2/d) + (\varepsilon /2) -\varepsilon }), $$

which tends to zero. Combined with Eq. 40 this shows that $\mathbb {E}[M_n(\Omega ,a_n)] \rightarrow 0$, as required. $\square$

### Proof of Theorem 2.9

In this subsection, we set $d=2$ and take *A* to be polygonal. Denote the vertices of *A* by $q_1,\ldots ,q_\kappa$, and the angles subtended at these vertices by $\alpha _1,\ldots ,\alpha _\kappa$ respectively.

To prove Theorem 2.9, we shall split *A* into an interior region, a region near the edges (but not the corners) and a region near the corners of *A*. We shall use Lemma 3.1 to determine the limiting probability of covering the interior region, and the results from Subsection 4.2 to determine the limiting probability of covering the region near the edges. We shall provide a separate argument to show the probability that the region near the corners is covered tends to 1.

For the duration of this subsection (and the next) we fix $\beta \in \mathbb {R}$. Assume we are given real numbers $(r_n)_{n >0}$ satisfying the case $d=2$ of Eq. 17, i.e.

46 $$\begin{aligned} \lim _{n \rightarrow \infty } \left( \pi n r_n^2 \mathbb {E}[Y^2] - \log n - (2k-1) \log \log n \right) = \beta . \end{aligned}$$

Fix ${\zeta }>0$. For $n >0$, in this subsection we define the ‘corner regions’ $Q_n$ and $Q_n^-$ by

$$ Q_n:= \cup _{j=1}^\kappa B(q_j,n^{3 {\zeta }}r_n) \cap A; ~~~~~ Q^-_n:= \cup _{j=1}^\kappa B(q_j, n^{2 {\zeta }} r_n) \cap A. $$

Also we define $\mathcal{U}_{n,A}$, $Z_n(\mathcal{U}_{n,A})$ and $Z^o_n(\mathcal{U}_{n,A})$ by Eqs. 26, 27 and 28.

#### Lemma 4.8

(Coverage of regions near the corners of *A*) Provided ${\zeta }$ is taken to be small enough, $\mathbb {P}[Q_n \subset Z_n^o(\mathcal{U}_{n,A})] \rightarrow 1$ as $n \rightarrow \infty$.

#### Proof

Let $$\delta >0$$ with $$\mathbb {P}[Y> 3 \delta ] > \delta $$. Then the restricted SPBM $$Z^o_n(\mathcal{U}_{n,A})$$ stochastically dominates a Poisson Boolean model $$Z'_n$$ with closed balls of deterministic radius $$2 \delta r_n$$, centred on the points of a homogeneous Poisson point process in *A* of intensity $$\delta n$$. Let $$Z''_n $$ be a Poisson Boolean model with closed balls of deterministic radius $$\delta r_n$$, centred on the points of a homogeneous Poisson point process in *A* of intensity $$\delta n$$.

There is a constant *K*, independent of *n*, such that for all $n \ge 1$ there exist points $y_{n,1},\ldots ,y_{n,\lfloor K n^{6 {\zeta }}\rfloor } \in A$ such that $Q_n \subset \cup _{j=1}^{\lfloor Kn^{6 {\zeta }}\rfloor } B(y_{n,j},\delta r_n)$. Also there is a constant $a >0$ such that for all $n \ge 1$ and all $y \in A$ we have $|B(y,\delta r_n) \cap A | \ge a r_n^2$. Then

47 $$\begin{aligned} \mathbb {P}[ Q_n \setminus Z_n^o(\mathcal{U}_{n,A}) \ne \emptyset ]&\le \mathbb {P}[ Q_n \setminus Z'_n \ne \emptyset ] \nonumber \\&\le \mathbb {P}[ \cup _{j=1}^{\lfloor K n^{6 {\zeta }} \rfloor } \{y_{n,j} \notin Z''_n\}] \nonumber \\&\le K n^{6 {\zeta }} k(n\pi r_n^2)^{k-1} \exp (- a \delta n r_n^2), \end{aligned}$$

and since $(n r_n^2)/\log n \rightarrow 1/(\pi \mathbb {E}[Y^2])$ by Eq. 46, provided we take ${\zeta }< a \delta /(6 \pi \mathbb {E}[Y^2])$ the expression in Eq. 47 tends to zero, and the result follows. $\square$

Recall from Eq. 26 the definition of the point processes $\mathcal{U}'_{n,D}$ for $n > 0, D \subset \mathbb {R}^d$.

#### Lemma 4.9

(Coverage of regions near the edges of *A*) Assume ${\zeta }< 1/d$. Then

48 $$\begin{aligned} \lim _{n \rightarrow \infty } ( \mathbb {P}[(\partial A \setminus Q^-_n) \subset Z_n(\mathcal{U}'_{n,A}) ]) = \exp \Big (- \Big ( \frac{c_{2,k} \mathbb {E}[Y]}{( \mathbb {E}[Y^2])^{1/2}} \Big ) |\partial A| e^{-\beta /2} \Big ). \end{aligned}$$

Also,

49 $$\begin{aligned} \lim _{n \rightarrow \infty } ( \mathbb {P}[\{A^{(3 n^{\zeta }r_n)} \subset Z_n(\mathcal{U}'_{n,A})\} \cap \{ (\partial A \cup Q_n ) \subset Z^o_n(\mathcal{U}'_{n,A})\} \setminus \{A \subset Z_n(\mathcal{U}'_{n,A})\}] ) = 0. \end{aligned}$$

#### Proof

Denote the line segments making up $$\partial A$$ by $$I_1,\ldots ,I_{\kappa }$$, and for $$ n >0$$ and $$1 \le i \le \kappa $$ set $$I_{n,i} := I_i \setminus Q^-_n$$.

Let $i,j,k \in \{1,\ldots ,\kappa \}$ be such that $i\ne j$ and the edges $I_i$ and $I_j$ are both incident to $q_k$. Provided *n* is large enough, if $x \in I_{n,i}$ and $y \in I_{n,j}$, then $\Vert x-y \Vert \ge (n^{2\zeta } r_n) \sin \alpha _k \ge 3 n^\zeta r_n$. Hence for all large enough *n* the events $\{I_{n,1} \subset Z_n( \mathcal{U}'_{n,A})\}, \ldots ,\{I_{n,\kappa } \subset Z_n( \mathcal{U}'_{n,A}) \}$ are mutually independent. Therefore

$$\begin{aligned} \mathbb {P}[ (\partial A \setminus Q^-_n) \subset Z_n(\mathcal{U}'_{n,A}) ] = \prod _{i=1}^\kappa \mathbb {P}[ I_{n,i} \subset Z_n( \mathcal{U}'_{n,A}) ], \end{aligned}$$

and by Lemmas 4.4 and 4.2, this converges to the right hand side of Eq. 48.

Now we prove (49). For $n >0$, and $i \in \{1,2,\ldots , \kappa \}$, let $S_{n,i}$ denote the rectangular block of dimensions $|I_{n,i} | \times 3 n^{\zeta }r_n$, consisting of all points in *A* at perpendicular distance at most $3 n^{\zeta }r_n$ from $I_{n,i}$. Let $\partial _{\textrm{side}}S_{n,i}$ denote the union of the two ‘short’ edges of $S_{n,i}$, i.e. the two edges bounding $S_{n,i}$ which are perpendicular to $I_{n,i}$.

Then for *n* large, $A \setminus (A^{(3 n^{\zeta }r_n)} \cup Q_n) \subset \cup _{i=1}^\kappa S_{n,i}$, and also $\partial _\textrm{side} S_{n,i} \subset Q_n$ for $1 \le i \le \kappa$, so that

$$\begin{aligned} \{ (A^{(3 n^\zeta r_n)} \cup \partial A ) \subset Z_n(\mathcal{U}'_{n,A})\} \cap \{ Q_n \subset Z^o_n(\mathcal{U}'_{n,A})\} \setminus \{ A \subset Z_n(\mathcal{U}'_{n,A}) \} ~~~~~~~~~~~~~~~~~~~~~~~ \\ \subset \cup _{i=1}^\kappa [\{I_{n,i} \subset Z_n(\mathcal{U}'_{n,A} ) \} \cup \{ \partial _{\textrm{side}} S_{n,i} \subset Z_n^o(\mathcal{U}'_{n,A} ) \} \setminus \{S_{n,i} \subset Z_n(\mathcal{U}'_{n,A})\}]. \end{aligned}$$

For $i \in \{1,\ldots ,\kappa \}$, let $I'_{n,i}$ denote an interval of length $|I_{n,i}|$ in $\mathbb {R}$. Using the Mapping theorem (Last and Penrose, 2017, Theorem 5.1) and the translation and rotation invariance of Lebesgue measure one sees that

$$\begin{aligned} \mathbb {P}[\{I_{n,i} \subset Z_n( \mathcal{U}'_{n,A}) \} \cap \{\partial _{\textrm{side}} S_{n,i} \subset Z^o_n( \mathcal{U}'_{n,A}) \} \setminus \{S_{n,i} \subset Z_n( \mathcal{U}'_{n,A})\}] \nonumber \\ = \mathbb {P}[ \{(I'_{n,i} \times \{0\}) \subset Z_n(\mathcal{U}'_{n,\mathbb {H}}) \} \cap \{ (\partial I'_{n,i}) \times [0,3n^\zeta r_n] \subset Z_n^o(\mathcal{U}'_{n,\mathbb {H}}) \} \nonumber \\ \setminus \{(I'_{n,i} \times [0,3n^{\zeta }r_n]) \subset Z_n(\mathcal{U}'_{n,\mathbb {H}})\} ] . \end{aligned}$$

By Lemma 4.7, this probability tends to zero, and hence (49). $\square$

#### Proof of Theorem 2.9

Suppose $(r_n)_{n >0}$ satisfies (46). We assert that provided ${\zeta }< 1/2$,

50 $$\begin{aligned} \lim _{n \rightarrow \infty } \mathbb {P}[ A^{(3 n^{\zeta }r_n)} \subset Z_n(\mathcal{U}'_{n,A}) ] = \exp \Big ( - \textbf{1}_{\{k=1\}} \Big ( \frac{(\mathbb {E}[Y])^2}{\mathbb {E}[Y^2]} \Big )|A| e^{-\beta } \Big ). \end{aligned}$$

Indeed, setting $\lambda = n$ and $\delta (\lambda ) =r_n$, we have that if $k=1$ then Eq. 19 holds, while if $k \ge 2$ then the left hand side of Eq. 19 tends to $+\infty$. Also $\mathbb {P}[ A^{(3 n^{\zeta }r_n)} \subset Z_n(\mathcal{U}'_{n,A})]$ is bounded from below by $\mathbb {P}[ A \subset Z_n(\mathcal{U}'_{n,\mathbb {R}^2})]$, which converges to the right hand side of Eq. 50 by Lemmas 3.1 and 4.2. Also, if $k=1$, then given $\varepsilon >0$, for *n* large the event $\{ A^{(3 n^{\zeta }r_n)} \subset Z_n(\mathcal{U}'_{n,A}) \}$ is contained in $\{ A^{[\varepsilon ]} \subset Z_n(\mathcal{U}'_{n,A})\}$, so by Lemmas 3.1 and 4.2 again

$$\begin{aligned} \limsup _{n \rightarrow \infty } \mathbb {P}[ A^{(n^{\zeta }r_n)} \subset Z_n(\mathcal{U}'_{n,A})]&\le \limsup _{n \rightarrow \infty } \mathbb {P}[ A^{[\varepsilon ]} \subset Z_n(\mathcal{U}'_{n,A})] \\&= \exp \Big ( - \Big ( \frac{(\mathbb {E}[Y])^2}{\mathbb {E}[Y^2]}\Big ) |A^{[\varepsilon ]}| e^{-\beta }\Big ), \end{aligned}$$

and since $|A^{[\varepsilon ]} | \rightarrow |A|$ as $\varepsilon \downarrow 0$, this gives us the assertion (50).

Also, by Eq. 48 and Lemma 4.8,

51 $$\begin{aligned} \lim _{n \rightarrow \infty } ( \mathbb {P}[\partial A \subset Z_n(\mathcal{U}'_{n,A}) ]) = \exp \Big (- \Big ( \frac{ c_{2,k} \mathbb {E}[Y] }{\sqrt{ \mathbb {E}[Y^2]} } \Big ) |\partial A| e^{-\beta /2} \Big ). \end{aligned}$$

Note $\mathbb {P}[\{( Q_n \cup \partial A ) \subset Z_n(\mathcal{U}'_{n,A})\} \setminus \{( Q_n \cup \partial A ) \subset Z^o_n(\mathcal{U}'_{n,A})\}]=0$. Therefore using Eq. 49 followed by Lemma 4.8, and then Lemma 4.2, we obtain that

52 $$\begin{aligned} \lim _{n \rightarrow \infty } \mathbb {P}[ A \subset Z_n(\mathcal{U}'_{n,A}) ]&= \lim _{n \rightarrow \infty } \mathbb {P}[ (A^{(3 n^{\zeta }r_n)} \cup \partial A \cup Q_n ) \subset Z_n(\mathcal{U}'_{n,A}) ] \nonumber \\&= \lim _{n \rightarrow \infty } \mathbb {P}[ (A^{(3 n^{\zeta }r_n)} \cup \partial A) \subset Z_n(\mathcal{U}'_{n,A}) ], \end{aligned}$$

provided these limits exist.

The events $\{\partial A \subset Z_n(\mathcal{U}'_{n,A}) \}$ and $\{A^{(3 n^{\zeta }r_n)} \subset Z_n(\mathcal{U}'_{n,A})\}$ are independent since the first of these events is determined by the configuration of Poisson points with projection onto $\mathbb {R}^2$ distant at most $n^\zeta r_n$ from $\partial A$, while the second event is determined by the Poisson points with projection distant at least $2 n^\zeta r_n$ from $\partial A$. Therefore the limit in Eq. 52 does indeed exist, and is the product of the limits arising in Eqs. 50 and 51. By Lemma 4.2 we get the same limit for $\mathbb {P}[A \subset Z_n(\mathcal{U}_{n,A})]$ as for $\mathbb {P}[A \subset Z_n(\mathcal{U}'_{n,A})]$. This gives us Eq. 14. $\square$

### First steps toward proving Theorem 2.10

In this subsection we assume that $d \ge 2$ and $\partial A \in C^2$. Let $k \in \mathbb {N}, \beta \in \mathbb {R}$, and assume that $(r_n)_{n >0}$ satisfies Eq. 17 and hence also Eqs. 24 and 25.

We shall now carry out the polytopal approximation strategy that was outlined in Subsection 2.3. We shall approximate *A* by a polytopal set $\Gamma _n$ with faces of width $O(n^{9 \zeta } r_n)$, where $\zeta >0$ is a fixed small positive constant. Thus the faces of $\Gamma _n$ will be small on a macroscopic scale, but large compared to $r_n$. We shall describe how to reassemble our Poisson process in regions near the faces of $\Gamma _n$ to get a Poisson process in the half-space $\mathbb {H}$, so that we can apply the results from subsection 4.2.

Given any $n >0$, Borel $D \subset \mathbb {R}^d$, and any point process $\mathcal {X}$ in $\mathbb {R}^d \times \mathbb {R}_+$, define $F_{n}(D,\mathcal {X})$ to be the event that *D* is ‘fully *k*-covered’ by the SPBM based on $\mathcal {X}$ with radii scaled by $r_n$, that is,

53 $$\begin{aligned} F_{n}(D,\mathcal {X}) := \{D \subset Z_n(\mathcal {X}) \}, \end{aligned}$$

where $Z_n(\cdot )$ was defined at Eq. 27. Also, given $r>0$, define the ‘covering number’

54 $$\begin{aligned} \kappa (D,r): = \min \{m \in \mathbb {N}: \exists x_1,\ldots ,x_m \in D ~\textrm{with} ~ D \subset \cup _{i=1}^m B(x_i,r) \}. \end{aligned}$$

Given $x \in \partial A$ we can express $\partial A$ locally in a neighbourhood of *x*, after a rotation, as the graph of a $C^2$ function from $\mathbb {R}^{d-1}$ to $\mathbb {R}$ with all of its partial derivatives taking the value zero at *x*. We shall approximate to that function by the graph of a piecewise affine function (in $d=2$, a piecewise linear function).

For each $x \in \partial A$, we can find an open neighbourhood $\mathcal{N}_x$ of *x*, a number $r(x) >0$ such that $B(x, 3r(x)) \subset \mathcal{N}_x$ and a rotation $\theta _x$ about *x* such that $\theta _x(\partial A \cap \mathcal{N}_x)$ is the graph of a real-valued $C^2$ function *f* defined on an open ball $D \subset \mathbb {R}^{d-1}$, with $\langle f'(x),e\rangle =0$ and $\langle f'(u),e\rangle \le 1/9$ for all $u \in D$ and all unit vectors *e* in $\mathbb {R}^{d-1}$, where $\langle \cdot ,\cdot \rangle$ denotes the Euclidean inner product in $\mathbb {R}^{d-1}$ and $f'(u):= (\partial _1 f(u),\partial _2f(u), \ldots , \partial _{d-1} f(u) )$ is the derivative of *f* at *u*. Moreover, by taking a smaller neighbourhood if necessary, we can also assume that there exist $\varepsilon >0$ and $a \in \mathbb {R}$ such that $f(u) \in [a+ \varepsilon ,a+ 2 \varepsilon ]$ for all $u \in D$ and also $\theta _x(A) \cap (D \times [a,a + 3 \varepsilon ]) = \{(u,z): u \in D, a \le z \le f(u)\}$.

By a compactness argument, we can and do take a finite collection of points $x_1,\ldots , x_J \in \partial A$ such that

55 $$\begin{aligned} \partial A \subset \cup _{j=1}^J B(x_j,r(x_j)). \end{aligned}$$

Then there are constants $$\varepsilon _j >0$$, and rigid motions $$\theta _j, 1 \le j \le J$$, such that for each *j* the set $$\theta _j(\partial A \cap \mathcal{N}_{x_j}) $$ is the graph of a $$C^2 $$ function $$f_j$$ defined on a ball $$I_j$$ in $$\mathbb {R}^{d-1}$$, with $$\langle f'_j(u), e \rangle \le 1/9$$ for all $$u \in I_j$$ and all unit vectors $$e \in \mathbb {R}^{d-1}$$, and also with $$\varepsilon _j \le f_j(u) \le 2 \varepsilon _j $$ for all $$u \in I_j$$ and $$\theta _j(A) \cap (I_j \times [0,3\varepsilon _j]) = \{(u,z):u \in I_j, 0 \le z \le f(u)\}$$.

Later we shall refer to each tuple $(x_j,r(x_j),\theta _j, f_j)$, $1 \le j \le J$, as a *chart*. More loosely we shall also refer to each set $B(x_j, r(x_j))$ as a chart.

Let $\Gamma \subset \partial A$ be a closed set such that $\Gamma \subset B(x_j,r(x_j))$ for some $j \in \{1,\ldots ,J\}$, and such that $\kappa (\partial \Gamma ,r) = O(r^{2-d})$ as $r \downarrow 0$, where we set $\partial \Gamma : = \Gamma \cap \overline{\partial A \setminus \Gamma }$, the boundary of $\Gamma$ relative to $\partial A$. To simplify notation we shall assume that $\Gamma \subset B(x_1,r(x_1))$, and moreover that $\theta _1$ is the identity map. Then $\Gamma = \{(u,f_1(u)): u \in U\}$ for some bounded set $U \subset \mathbb {R}^{d-1}$. Also, writing $\phi (\cdot )$ for $f_1(\cdot )$ from now on, we assume

56 $$\begin{aligned} \phi (U) \subset [\varepsilon _1,2 \varepsilon _1] \end{aligned}$$

and

57 $$\begin{aligned} A \cap (U \times [0,3 \varepsilon _1]) = \{(u,z): u \in U, 0 \le z \le \phi (u) \}. \end{aligned}$$

Note that for any $u,v \in U$, by the Mean Value theorem we have for some $w \in [ u,v]$ that

58 $$\begin{aligned} |\phi (v) - \phi (u) | = | \langle v-u, \phi '(w)\rangle | \le (1/9) \Vert v-u\Vert . \end{aligned}$$

Choose (and keep fixed for the rest of this paper) a constant ${\zeta }$ with

59 $$\begin{aligned} 0< {\zeta }< 1/(198d(18+d)). \end{aligned}$$

Henceforth we shall use this choice of ${\zeta }$ in the definition of $\mathcal{U}'_{n,D}$ at Eq. 29. We shall use $n^{c{\zeta }} r_n$, for various choices of constant *c*, to provide various different length scales.

We shall approximate to $\Gamma$ by a polytopal surface $\Gamma _n$ with face diameters that are $\Theta (n^{9 {\zeta }} r_n)$, taking all the faces of $\Gamma _n$ to be $(d-1)$-dimensional simplices. Later we shall fit together a finite number of surfaces like $\Gamma$ to make up the whole of $\partial A$.

**Fig. 6:**
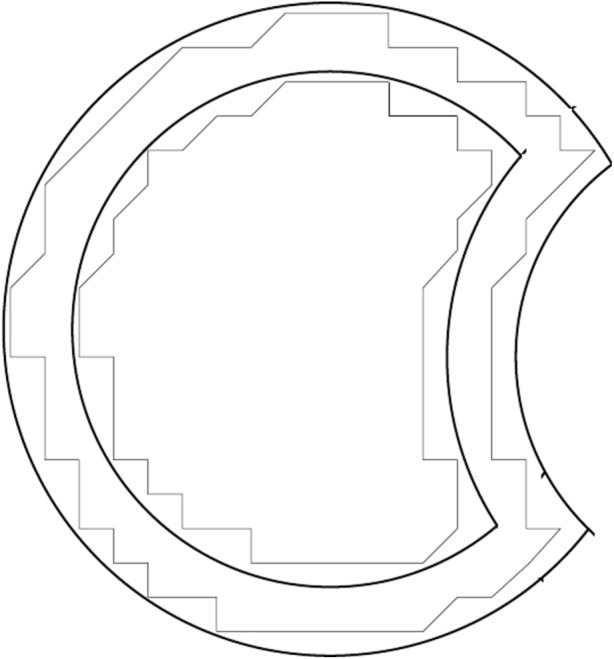
Example in $d=3$. The outer crescent-shaped region is *U*, while the inner crescent is $U^{(10 {\zeta }r_n)}$. The outer polygon is $U_n$, while the inner polygon is $U_n^-$

For the polytopal approximation, divide $\mathbb {R}^{d-1}$ into hypercubes of dimension $d-1$ and side $n^{9 {\zeta }} r_n$, and divide each of these hypercubes into $(d-1)!$ simplices (we take these simplices to be closed). Let $U_n$ be the union of all those simplices in the resulting tessellation of $\mathbb {R}^{d-1}$ into simplices, that are contained within *U*, and let $U_n^-$ be the union of those simplices in the tessellation which are contained within $U^{(n^{10 {\zeta }} r_n)}$, where for $r>0$ we set $U^{(r)}$ to be the set of $x \in U$ at a Euclidean distance more than *r* from $\mathbb {R}^{d-1} \setminus U$. If $d=2$, the simplices are just intervals. See Fig. 6.

Let $\sigma ^-(n)$ denote the number of simplices making up $U_n^-$. Choose $n_0 >0$ such that $\sigma ^-(n) >0$ for all $n \ge n_0$.

Let $\psi _n: U_n \rightarrow \mathbb {R}$ be the function that is affine on each of the simplices making up $U_n$, and agrees with the function $\phi$ on each of the vertices of these simplices.

**Fig. 7:**
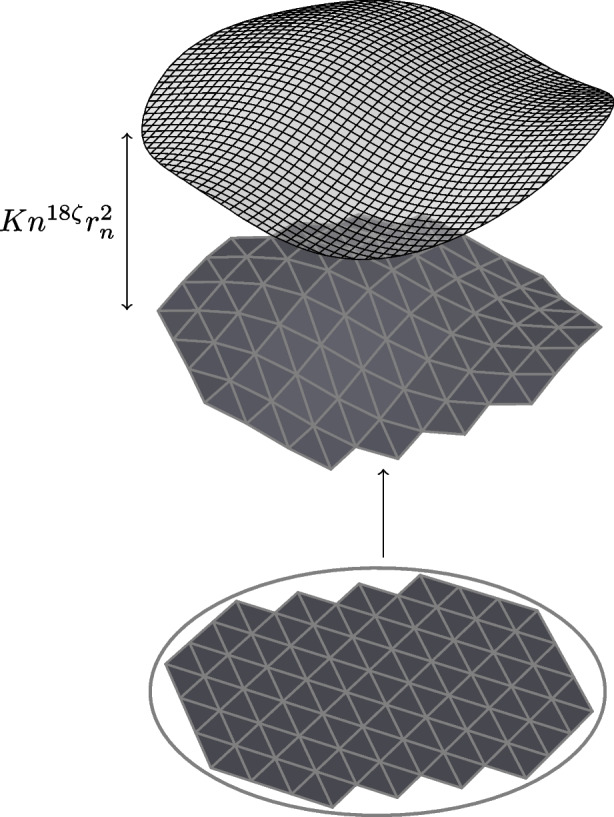
We show here the set $\Gamma _n$ below a portion $\Gamma$ of the boundary of *A*, when $d=3$. The bottom part of the diagram shows the simplices (triangles) making up $U_n^-$, which are used to construct the triangulated surface above it. The faces of the triangulated surface $\Gamma _n$ are the $H_{n,i}$

Such a function exists because, if one such simplex has vertices labelled $v_1,v_2,\ldots ,v_d$ say, then $v_2-v_1, \ldots , v_d- v_1$ are linearly independent in $\mathbb {R}^{d-1}$ (in general it would not exist if we used cubes instead of simplices because a cube would have too many vertices). Our approximating surface (or polygonal line if $d=2$) will be defined by $\Gamma _n:= \{(u, \psi _n(u)-K n^{18 {\zeta }}r_n^2): u \in U^-_n\}$, as depicted in Fig. 7 for the case $d=3$, with the constant *K* given by the following lemma. This lemma uses Taylor expansion to show that $\psi _n$ a good approximation to $\phi$.

#### Lemma 4.10

(Polytopal approximation) Set $K := \sup _{n \ge n_0, u \in U_n} ( (n^{9 {\zeta }} r_n)^{-2} |\phi (u) - \psi _n(u) |)$. Then $K < \infty$.

#### Proof

See Penrose (2023), Lemma 7.18. The notation *t* there is equivalent to *n* here. the length scale of $n^{9\zeta } r_n$ here plays the role of $t^{-\gamma }$ there. $\square$

We now subtract a constant from $\psi _n$ to obtain a piecewise affine function $\phi _n$ that approximates $\phi$ from below. For $n \ge n_0$ and $u \in U_n$, define $\phi _n(u) := \psi _n(u) - K n^{18 {\zeta }}r_n^2$, with *K* given by Lemma 4.10. Then for all $n \ge n_0, u \in U_n$ we have $|\psi _n(u)-\phi (u)|\le Kn^{18 {\zeta }} r_n^2$ so that

60 $$\begin{aligned} \phi _n(u) \le \phi (u) \le \phi _n(u) + 2K n^{18 {\zeta }} r_n^2. \end{aligned}$$

Define the set $\Gamma _n: = \{(u,\phi _n(u)): u \in U_n^-\}$. We refer to each $(d-1)$-dimensional face of $\Gamma _n$ (given by the graph of $\phi _n$ restricted to one of the simplices in our triangulation of $\mathbb {R}^{d-1}$) as simply a *face* of $\Gamma _n$. Denote these faces of $\Gamma _n$ by $H_{n,1}, \ldots , H_{n,\sigma ^-(n)}$. The number of faces, $\sigma ^-(n)$, is $\Theta ((n^{9 {\zeta }} r_n)^{1-d})$ as $n \rightarrow \infty$. The perimeter (i.e., the $(d-2)$-dimensional Hausdorff measure of the boundary) of each individual face is $\Theta ((n^{9 {\zeta }} r_n)^{d-2})$. For $1 \le i \le \sigma ^-(n)$, let $\partial _{d-2}H_{n,i}$ denote the boundary of $H_{n,i}$, which is the image under the mapping $u \mapsto (u,\phi _n(u))$, of the boundary of the simplex in $\mathbb {R}^{d-1}$ that is the pre-image of $H_{n,i}$ under that mapping.

**Fig. 8:**
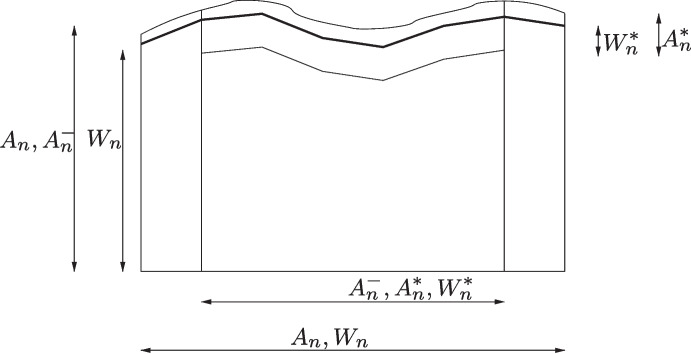
When $$d=2$$ the sets $$A_n, W_n, A_n^-, A_n^*, W_n^*$$ are approximately rectangular but with a curved upper boundary for $$A_n, A_n^-$$ and $$ A_n^*$$, a polygonal upper boundary for $$W_n^*$$ and $$W_n$$, and a polygonal lower boundary for $$A_n^*$$ and $$W_n^*$$. The ‘faces’ $$H_{n,i}$$ are just line segments since $$d=2$$, and are the segments of the bold polygonal line

For $n \ge n_0$, define subsets $A_n,A_n^-, W_n, A_n^{*}, W_n^*$ of $\mathbb {R}^d$ (illustrated in Fig. 8) by

61 $$\begin{aligned} A_n := \{(u,z): u \in U_n, 0 \le z \le \phi (u)\}, ~~~ W_n := \{(u,z): u \in U_n, 0 \le z \le \phi _n(u)\}, \nonumber \\ A_n^- := \{(u,z): u \in U_n^-, 0 \le z \le \phi (u)\}, ~~~~~ ~~~~~ ~~~~~ ~~~~~ ~~~~~ ~~~~~ ~~~~~ ~ \nonumber \\ A_n^* := \{(u,z): u \in U_n^-, \phi _n(u) - (3/2) n^{\zeta }r_n \le z \le \phi (u)\}, ~~~~~ ~~~~~ ~~~~~ ~~ ~~ \nonumber \\ W_n^* := \{(u,z): u \in U_n^-, \phi _n(u) - (3/2) n^{\zeta }r_n \le z \le \phi _n(u)\}. ~~~~~~~~ ~~~~~~~~ ~ \end{aligned}$$

Thus $A_n$ is a ‘thick slice’ of *A* near the boundary region $\Gamma$, $W_n$ is an approximating region having $\Gamma _n$ as its upper boundary, and $A_n^{*}$, $W_n^*$ are ‘thin slices’ of *A* also having $\Gamma$, respectively $\Gamma _n$, as upper boundary. By Eqs. 60, 56 and 57, $W_n^* \subset A_n^* \subset A_n^- \subset A_n \subset A$, and $W_n^* \subset W_n \subset A_n$.

The rest of this subsection, and the next subsection, are devoted to proving the following intermediate step towards a proof of Theorem 2.10. Recall the definition of $c_{d,k}$ at Eq. 15, and define

62 $$\begin{aligned} c_{d,k,Y} := \frac{c_{d,k} (\mathbb {E}[Y^{d-1}])^{d-1}}{(\mathbb {E}[Y^d])^{d-2+1/d}}. \end{aligned}$$

#### Proposition 4.11

(Limiting coverage probability for approximating polytopal shell) It is the case that $\lim _{n \rightarrow \infty } \mathbb {P}[F_{n}(W_n^*, \mathcal{U}'_{n,W_{n}}) ] = \exp (- c_{d,k,Y} |\Gamma | e^{- \beta /2} ),$ where $|\Gamma |$ denotes the $(d-1)$-dimensional Hausdorff measure of $\Gamma$.

The following corollary of Lemma 4.10 is a first step towards proving this.

#### Lemma 4.12

(a) It is the case that $|A_n \setminus W_n| = O(n^{18 {\zeta }}r_n^2)$ as $n \rightarrow \infty$.

(b) Let *K* be as given in Lemma 4.10, and fix $L>0$. Then for all $n \ge n_0$ and $x \in U_n^{(Lr_n)} \times \mathbb {R}$, $|B(x,L r_n) \cap A_n \setminus W_n| \le 2K \omega _{d-1} L^{d-1} n^{18 {\zeta }} r_n^{d+1}$.

#### Proof

Since $|A_n \setminus W_n | = \int _{U_n} (\phi (u) - \phi _n(u)) du$, where this is a $(d-1)$-dimensional Lebesgue integral, part (a) comes from Eq. 60.

For (b), let $x \in U_n^{(L r_n)} \times \mathbb {R}$, and let $u \in U_n^{(L r_n)}$ be the projection of *x* onto the first $d-1$ coordinates. Then if $y \in B(x,Lr_n) \cap A_n \setminus W_n$, we have $y = (v,s)$ with $\Vert v-u\Vert \le L r_n$ and $\phi _n(v) < s \le \phi (v)$. Therefore using Eq. 60 yields

$$ |B(x,Lr_n) \cap A_n \setminus W_n| \le \int _{B_{(d-1)}(u,Lr_n)} (\phi (v) - \phi _n(v)) dv \le 2K \omega _{d-1} n^{18 {\zeta }} r_n^2 L^{d-1} r_n^{d-1}, $$

where the integral is a $(d-1)$-dimensional Lebesgue integral. This gives part (b). $\square$

The next lemma says that small balls centred in $A_n^*$ have almost half of their volume in $W_n$.

#### Lemma 4.13

Let $\varepsilon \in (0,1)$. Then for all large enough *n*, all $x \in A_n^*$, and all $s \in [\varepsilon r_n,r_n/\varepsilon ]$, we have $|B(x,s) \cap W_n|> (1- \varepsilon ) (\omega _d/2) s^d$.

#### Proof

For all large enough *n*, all $x \in A_n^*$ and $s \in [\varepsilon r_n,r_n/\varepsilon ]$, we have $B(x,s )\cap A \subset A_n$, so $B(x,s) \cap A_n = B(x,s) \cap A$, and hence by Lemma 4.1 and Lemma 4.12(b),

$$\begin{aligned} |B(x,s) \cap W_n|= &  |B(x,s) \cap A_n | - |B(x,s ) \cap A_n \setminus W_n |\\ \nonumber\ge &  (1 - \varepsilon /2) (\omega _d /2) s^d - O( n^{18 {\zeta }} r_n^{d+1} ). \nonumber \end{aligned}$$

Since $n^{18 {\zeta }} r_n \rightarrow 0$ by Eq. 17 and Eq. 59, this gives us the result. $\square$

Recall that $\mathbb {H}$ and $\mathcal{U}'_{n,D}$ were defined in Section 4.1 and at Eq. 29, respectively. The next lemma provides a bound on the probability that a region of diameter $O(r_n)$ within *A* or $A_n^*$ is not fully covered. This will be used for dealing with ‘exceptional’ regions such as those near the boundaries of faces in the polytopal approximation.

#### Lemma 4.14

Let $\varepsilon \in (0,1)$, $K_1 >0$. Then as $n \rightarrow \infty$,

63 $$\begin{aligned} \sup _{z \in \mathbb {R}^d} \mathbb {P}[ F_{n}(B(z,K_1r_n) \cap A_n^*, \mathcal{U}'_{n,W_n} )^c ] = O(n^{\varepsilon - (d-1)/d}), \end{aligned}$$

64 $$\begin{aligned} \sup _{z \in \mathbb {R}^d} \mathbb {P}[ F_{n}(B(z,K_1r_n) \cap \mathbb {H},\mathcal{U}'_{n ,\mathbb {H}} )^c ] = O(n^{\varepsilon - (d-1)/d}), \end{aligned}$$

and

65 $$\begin{aligned} \sup _{z \in \mathbb {R}^d} \mathbb {P}[ F_{n}(B(z,K_1r_n) \cap A , \mathcal{U}'_{n,A} )^c ] = O(n^{\varepsilon - (d-1)/d}). \end{aligned}$$

#### Proof

Let $\delta \in (0,1/2)$ with $(1-\delta )^{d+1} \mathbb {E}[Y^d\textbf{1}_{\{\delta \le Y \le 1/\delta \}}] > (1-\varepsilon /2) \mathbb {E}[Y^d]$. For any point set $\mathcal {X}\subset \mathbb {R}^d \times [\delta ,\infty )$, define $\tilde{Z}_n(\mathcal {X})$ similarly to $Z_n(\mathcal {X})$ but with slightly smaller balls, namely

$$ \tilde{Z}_n(\mathcal {X}) : = \{y \in \mathbb {R}^d: \# (\{(x,s) \in \mathcal {X}: y \in B(x,(1-\delta )r_ns)\}) \ge k\}, $$

and note that for any $y \in \tilde{Z}_n(\mathcal {X})$ we have $B(y,\delta ^2 r_n) \subset Z_n(\mathcal {X})$.

There is a constant $\ell$ independent of *n* such that given $z \in \mathbb {R}^d$ and given *n*, we can (and do) choose $x_{n,1},\ldots ,x_{n,\ell } \in A_n^*$ with $B(z,K_1r_n) \cap A^*_n \subset \cup _{i=1}^\ell B(x_{n,i}, \delta ^2 r_n)$. Then for all *n* large enough, and for $1 \le i \le \ell$, using Lemma 4.13 in the third line below we have

$$\begin{aligned} 1- \mathbb {P}[&F_{n}(B(x_{n,i},\delta ^2 r_n) \cap A^*_n, \mathcal{U}'_{n,W_n})] \le \mathbb {P}[ x_{n,i} \notin \tilde{Z}_n (\mathcal{U}'_{n,W_n} \cap (W_n \times [\delta ,1/\delta ] )) ] \\&\le k (n \omega _d (r_n/\delta )^d )^{k-1} \exp \Big ( - n \int _{[\delta ,1/\delta ]} |W_n \cap B(x_i,(1-\delta )r_n t)|\mu _Y(dt) \Big ) \\&\le k (n \omega _d (r_n/\delta )^d )^{k-1} \exp \Big ( - n \int _{[\delta ,1/\delta ]} (1-\delta )^{d+1} (\omega _d/2) r_n^d t^d\mu _Y(dt) \Big ) \\&\le k (n \omega _d (r_n/\delta )^d )^{k-1} \exp \Big ( - ( \omega _d/2) n r_n^d (1- \varepsilon /2) \mathbb {E}[Y^d] \Big ). \end{aligned}$$

Therefore by Eq. 25, for *n* large

$$\begin{aligned} \mathbb {P}[\{F_{n}(B(x_{n,i},\delta ^2 r_n) \cap A_n^*,\mathcal{U}'_{n,W_n})\}^c] \le n^{\varepsilon - (d-1)/d}. \end{aligned}$$

Taking the union of the above events for $1 \le i \le \ell$, and applying the union bound, gives us Eq. 63.

The proofs of Eqs. 64 and 65 are similar. $\square$

Let $\partial _{d-2} \Gamma _n := \cup _{i=1}^{\sigma ^-(t)} \partial _{d-2} H_{n,i}$, the union of all $(d-2)$-dimensional faces in the boundaries of the faces making up $\Gamma _n$ (the $H_{n,i}$ were defined just after (60)). Recall from Eq. 59 that ${\zeta }\in (0,1/(198d(18+d)))$. Given $n >0$, define the set $Q^+_n \subset \mathbb {R}^d$ by

66 $$\begin{aligned} Q^+_n := (\partial _{d-2} \Gamma _n \oplus B(o,8dn^{4{\zeta }} r_n)) \cap A_n^{*}. \end{aligned}$$

Thus $Q_n^+$ is a region near the corners of our polygon approximating $\partial A$ (if $d=2$) or near the boundaries of the faces of our polytopal surface approximating $\partial A$ (if $d \ge 3$). In the next lemma we show that $Q_n^+$ is fully *k*-covered with high probability.

#### Lemma 4.15

It is the case that

$$\mathbb {P}[F_{n}(Q^+_n,\mathcal{U}'_{n,W_n})] \rightarrow 1$$ as $$n \rightarrow \infty $$.

#### Proof

Let $\varepsilon := {\zeta }/2$. For each face $H_{n,i}$ of $\Gamma _n$, $1 \le i \le \sigma ^-(n)$, we claim that we can take $x_{i,1},\ldots , x_{i,k_{n,i}} \in \mathbb {R}^d$ with $\max _{1 \le i \le \sigma ^-(n)} k_{n,i} = O(n^{9 d {\zeta }-10 {\zeta }} )$, such that

67 $$\begin{aligned} (\partial _{d-2} H_{n,i}) \oplus B(o, 8 dn^{4 {\zeta }}r_n) \subset \cup _{j=1}^{k_{n,i}} B(x_{i,j},r_n). \end{aligned}$$

Indeed, we can cover $\partial _{d-2} H_{n,i}$ by $O(n^{5 {\zeta }(d-2)})$ balls of radius $n^{4{\zeta }} r_n$, denoted $B_{i,\ell }^{(0)}$ say. Replace each ball $B_{i,\ell }^{(0)}$ with a ball $B'_{i,\ell }$ with the same centre as $B_{i,\ell }^{(0)}$ and with radius $9d n^{4 {\zeta }}r_n$. Then cover $B'_{i,\ell }$ by $O((n^{4 {\zeta }} )^{d})$ balls of radius $r_n$. Every point in the set on the left side of Eq. 67 lies in one of these balls of radius $r_n$, and the claim follows.

By the definitions of $Q_n^+$ and $\partial _{d-1}\Gamma _n$, and then Eq. 67, we have

$$ Q_n^+ \subset \cup _{i=1}^{\sigma ^-(n)}(( \partial _{d-2} H_{n,i}) \oplus B(o,8dn^{4{\zeta }}r_n)) \subset \cup _{i=1}^{\sigma ^-(n)} \cup _{j=1}^{k_{n,i}} B(x_{i,j}, r_n),$$

so by the union bound,

$$\begin{aligned} \mathbb {P}[ F_{n} ( Q^+_n ,\mathcal{U}'_{n,W_n}) ^c] \le \sum _{i=1}^{\sigma ^-(n)} \sum _{j=1}^{k_{n,i}} \mathbb {P}[ F_{n}( B(x_{i,j}, r_n) \cap A_n^*, \mathcal{U}'_{n,W_n} )^c]. \end{aligned}$$

Thus using Lemma 4.14 and the fact that $\sigma ^-(n) = O((n^{9 {\zeta }}r_n)^{1-d})$,

we obtain that

$$\begin{aligned} \mathbb {P}[ F_{n} ( Q^+_n ,\mathcal{U}'_{n,W_n}) ^c]&= O((n^{9 {\zeta }} r_n)^{1-d}n^{(9d-10){\zeta }} n^{\varepsilon -1 + 1/d} ) \\&=O(n^{ - {\zeta }} r_n^{1-d} n^{\varepsilon - 1 +1/d}) = O(n^{-{\zeta }/2}). \end{aligned}$$

Thus $\mathbb {P}[ F_{n} ( Q^+_n ,\mathcal{U}'_{n,W_n}) ^c]$ tends to zero. $\square$

### Induced coverage process and proof of Proposition 4.11

In this subsection we shall conclude the proof of Proposition 4.11, concerning the limiting probability of covering an approximating polytopal shell. We shall do so by means of a device we refer to as the *induced coverage process.* This is obtained by taking the parts of $W_n$ near the flat parts of $\Gamma _n$, along with any Poisson points therein, and rearranging them into a flat region of macroscopic size.

**Fig. 9:**
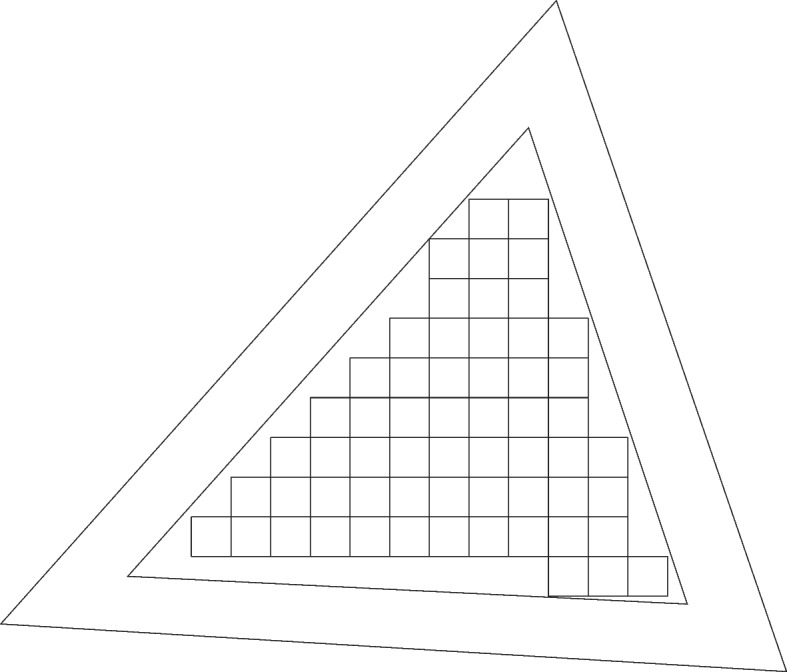
Part of the ‘plaid’ region $P_n$ when $d=3$. The outer triangle represents one face $H_{n,i}$, and the part of $P_n$ within $H_{n,i}$ is given by the union of the boundaries of the squares. The squares themselves are some of the $I_{n,i}^+$. The outer triangle has sides of length $\Theta (n^{9 {\zeta }} r_n)$, while the squares have sides of length $n^{3 {\zeta }} r_n$. The region between the two triangles has thickness $n^{4 {\zeta }} r_n$ and is contained in $Q_n^+$

Partition each face $H_{n,i}$, $1 \le i \le \sigma ^-(n)$ into a collection of $(d-1)$-dimensional hypercubes of side $n^{3{\zeta }}r_n$ contained in $H_{n,i}$ and distant more than $n^{4 {\zeta }}r_n$ from $\partial _{d-2}H_{n,i}$, together with a ‘border region’ contained within $\partial _{d-2} H_{n,i} \oplus B(o,2 n^{4 {\zeta }}r_n)$. Let $P_n$ be the union (over all faces) of the boundaries of the $(d-1)$-dimensional hypercubes in this partition (see Fig. 9: the *P* stands for ‘plaid’). Set

68 $$\begin{aligned} P^+_n := [ P_n \oplus B(o, 9 n^{ {\zeta }} r_n) ] \cap W_n^{*}. \end{aligned}$$

Enumerate the $(d-1)$-dimensional hypercubes in the above subdivision of the faces $H_{n,i}, 1 \le i \le \sigma ^-(n)$, as $I^+_{n,1},\ldots , I^+_{n,\lambda (n)}$. For $1 \le i \le \lambda (n)$ let $I_{n,i}:= I^+_{n,i} \setminus (P_n \oplus B(o, n^{\zeta }r_n))$, which is a $(d-1)$-dimensional hypercube of side length $(n^{3{\zeta }} - 2n^{\zeta }) r_n$ with the same centre and orientation as $I_{n,i}^+$. Writing $|\cdot |$ below for $(d-1)$-dimensional Hausdorff measure, we claim that the total $(d-1)$-dimensional Hausdorff measure of these $(d-1)$-dimensional hypercubes satisfies

69 $$\begin{aligned} \lim _{n \rightarrow \infty }( | \cup _{i=1}^{\lambda (n)} I_{n,i} | ) = |\Gamma |. \end{aligned}$$

Indeed, for $1 \le i \le \lambda (n)$ we have $|I_{n,i}|/|I_{n,i}^+| = ((n^{3 {\zeta }} - n^{\zeta }) /n^{3{\zeta }} )^{d-1}$, which tends to one, so the proportionate amount removed near the boundaries of the $(d-1)$-dimensional hypercubes $I^+_{n,i}$ to give $I_{n,i}$ vanishes. Also the ‘border region’ of a face $H_{n,i}$ that is not contained in any of the of the $I^+_{n,j}$s has $(d-1)$-dimensional Hausdorff measure that is $O((n^{9 {\zeta }} r_n)^{d-2} n^{4{\zeta }}r_n)$, so that the total $(d-1)$-dimensional measure of the removed regions near the boundaries of the faces is $O((n^{9 {\zeta }} r_n)^{1-d} \times (n^{9{\zeta }} r_n)^{d-2} n^{4{\zeta }}r_n) = O(n^{ -5 {\zeta }} )$, which tends to zero. Thus the claim (69) is justified.

**Fig. 10:**
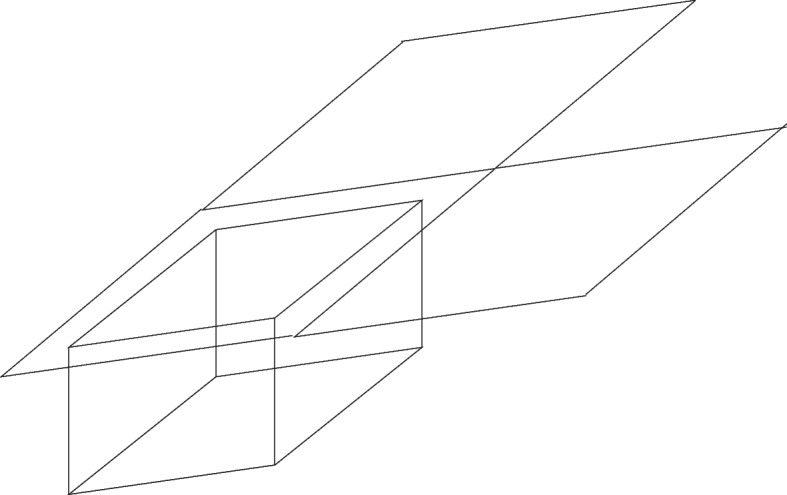
Diagram for $d=3$ showing three of the squares $I_{n,i}^+$ (as in Fig. 9 but now shown with 3-dimensional perspective), along with one of the squares $I_{n,i}$ (a slightly smaller square) and the corresponding cube $S_{n,i}$

For $1 \le i \le \lambda (n)$, let $S_{n,i}^+$, respectively $S_{n,i}$, be a *d*-dimensional cube of side $n^{3{\zeta }} r_n$, respectively of side $(n^{3{\zeta }} -2 n^{\zeta })r_n$, having $I^+_{n,i}$, respectively $I_{n,i}$, as its ‘upper face’, i.e. having $I_{n,i}^+$ (resp. $I_{n,i}$) as a face and lying within $W_n$. See Fig. 10. We shall verify in Lemma 4.17 that $S_{n,1}^+,\ldots , S_{n,\lambda (n)}^+$ are disjoint.

Define a region $D_n \subset \mathbb {R}^{d-1}$ that is approximately a rectilinear hypercube with lower left corner at the origin, and obtained as the union of $\lambda (n)$ disjoint $(d-1)$-dimensional hypercubes of side $n^{3 {\zeta }} r_n$. We can and do arrange that $D_n \subset [0,|\Gamma _n|^{1/(d-1)}+ n^{3{\zeta }}r_n]^{d-1}$ for each *n*, and $|D_n| \rightarrow |\Gamma |$ as $n \rightarrow \infty$. Define the flat slabs (or if $d=2$, flat strips)

70 $$\begin{aligned} {\textsf{S}}_n:= D_n \times [0, (n^{3{\zeta }} -2 n^{\zeta }) r_n]; ~~~~~~ {\textsf{S}}_n^+ := D_n \times [0, n^{3 {\zeta }} r_n], \end{aligned}$$

and denote the lower boundary of ${\textsf{S}}_n$ (that is, the set $D_n \times \{0\}$) by $L_n$.

Now choose rigid motions $\theta _{n,i}$ of $\mathbb {R}^d$, $1 \le i \le \lambda (n)$, such that under applications of these rigid motions the blocks $S^+_{n,i}$ are reassembled to form the slab ${\textsf{S}}_n^+$, with the square face $I^+_{n,i}$ of the *i*-th block transported to part of the lower boundary $L_n$ of ${\textsf{S}}_n$. In other words, choose the rigid motions so that the sets $\theta _{n,i}(S^+_{n,i})$, $1 \le i \le \lambda (n)$, have pairwise disjoint interiors and their union is ${\textsf{S}}_n^+$, and also $\theta _{n,i}(I^+_{n,i}) \subset L_n$ for $1 \le i \le \lambda (n)$.

Recall that $n_0$ was chosen shortly before Lemma 4.10. Given $n \ge n_0$ and $i \le \lambda (n)$, define $\tilde{\theta }_{n,i}: \mathbb {R}^{d+1} \rightarrow \mathbb {R}^{d+1}$ by $\tilde{\theta }_{n,i}(x,r) = (\theta _{n,i}(x),r)$ for all $x \in \mathbb {R}^d, r \in \mathbb {R}$. By the Restriction, Mapping and Superposition theorems (see e.g. Last and Penrose (2017)), the point process $\tilde{\mathcal{U}}_{n,{\textsf{S}}_n^+} := \cup _{i=1}^{\lambda (n)} \tilde{\theta }_{n,i} (\mathcal{U}_{n,W_n} \cap (S^+_{n,i} \times \mathbb {R}_+))$ is a Poisson process in ${\textsf{S}}_n^+ \times \mathbb {R}_+$ with intensity measure $n\textrm{Leb}_d \otimes \mu _Y$ (strictly speaking, with intensity measure $n\textrm{Leb}_d|_{{\textsf{S}}_n^+} \otimes \mu _Y$).

We extend $\tilde{\mathcal{U}}_{n,{\textsf{S}}_n^+}$ to a Poisson process $\tilde{\mathcal{U}}_{n,\mathbb {H}}$ in $\mathbb {H}\times \mathbb {R}_+$, where $\mathbb {H}:= \mathbb {R}^{d-1} \times [0,\infty )$, as follows. Let $\tilde{\mathcal{U}}_{n,\mathbb {H}\setminus {\textsf{S}}_n^+}$ be a Poisson process with intensity measure $n \textrm{Leb}_d \otimes \mu _Y$ in $(\mathbb {H}\setminus {\textsf{S}}_n^+) \times \mathbb {R}_+$, independent of $\mathcal{U}_{n,W_n}$, and set

71 $$\begin{aligned} \tilde{\mathcal{U}}_{n,\mathbb {H}}:= \tilde{\mathcal{U}}_{n,{\textsf{S}}_n^+} \cup \tilde{\mathcal{U}}_{n,\mathbb {H}\setminus {\textsf{S}}_n^+}; ~~~~~~~~~~~~~~~\tilde{\mathcal{U}}'_{n,\mathbb {H}}:= \tilde{\mathcal{U}}_{n,\mathbb {H}} \cap (\mathbb {R}^d \times [0,n^{\zeta }r_n]). \end{aligned}$$

By the Superposition theorem (see e.g. Last and Penrose (2017), Theorem 3.3), $\tilde{\mathcal{U}}_{n,\mathbb {H}}$ is a homogeneous Poisson process in $\mathbb {H}\times [0,\infty )$ with intensity measure $n\textrm{Leb}_d \times \mu _Y$. We call the collection of balls of radius $r_nt$ centred on *x*, where $\{(x,t)\}$ are the points of this point process, the *induced coverage process*.

The next lemma says that the region $P_n^+$ is covered with high probability. It is needed because locations in $P_n^+$ lie near the boundary of blocks $S_{n,i}$, so that coverage of these locations by $Z_n(\mathcal{U}_{n,A})$ does not necessarily correspond to coverage of their images in the induced coverage process.

#### Lemma 4.16

It is the case that $\lim _{n \rightarrow \infty } \mathbb {P}[ F_n(P^+_n, \mathcal{U}'_{n,W_n}) ] =1.$

#### Proof

We have $\lambda (n) = O((n^{3{\zeta }} r_n)^{1-d})$, and for $1 \le i \le \lambda (n)$, the number of balls of radius $n^{\zeta }r_n$ required to cover the boundary of the $(d-1)$-dimensional hypercube $I_{n,i}^+$ is $O((n^{3{\zeta }}/n^{\zeta })^{d-2})$. Thus we can take $x_{n,1}, \ldots , x_{n,k_n} \in \mathbb {R}^d$, with $k_n = O(r_n^{1-d}n^{-{\zeta }-d{\zeta }} )$, such that $P_n \subset \cup _{i=1}^{k_n} B(x_{n,i}, n^{\zeta }r_n)$.

Then $$P_n^+ \subset \cup _{i=1}^{k_n} B(x_{n,i},10 n^{\zeta }r_n) \cap A_n^*$$,

and $B(x_{n,i},10 n^{\zeta }r_n)$ can be covered by $O(n^{d{\zeta }})$ balls of radius $r_n$.

Hence by taking $\varepsilon = {\zeta }/2$ in Lemma 4.14, and using Eq. 17, we have

$$\begin{aligned} \mathbb {P}[ F_n(P^+_n, \mathcal{U}'_{n,W_n}) ^c]&\le \sum _{i=1}^{k_n} \mathbb {P}[F_n(B(x_{n,i},10 n^{\zeta }r_n) \cap A_n^*, \mathcal{U}'_{n,W_n} )^c] \\&= O(r_n^{1-d} n^{-{\zeta }-d{\zeta }} n^{d{\zeta }} n^{\varepsilon - (d-1)/d}) = O(n^{- {\zeta }/2 }), \end{aligned}$$

which tends to zero. $\square$

#### Lemma 4.17

Suppose $$n \ge n_0$$ and $$i < j \le \lambda (n)$$, $$i,j \in \mathbb {N}$$.

Then $$(S_{n,i}^+)^o \cap (S_{n,j}^+)^o = \emptyset $$.

#### Proof

Suppose $(S_{n,i}^+)^o \cap (S_{n,j}^+)^o \ne \emptyset$; we shall obtain a contradiction. Let $x \in (S_{n,i}^+)^o \cap (S_{n,j}^+)^o$. Let *y* be the closest point in $I^+_{n,i}$ to *x*, and $y'$ the closest point in $I^+_{n,j}$ to *x*. Choose $\ell ,m$ such that $I^+_{n,i} \subset H_{n,\ell }$ and $I^+_{n,j} \subset H_{n,m}$. Then $\ell \ne m$ since if $\ell = m$ we would clearly have $(S_{n,i}^+)^o \cap (S_{n,j}^+)^o = \emptyset$.

Let $J_{n,\ell } \subset \mathbb {R}^{d-1}$ be the image of $H_{n,\ell }$ under projection onto the first $d-1$ coordinates, and write $y= (u,\phi _n(u))$ with $u \in J_{n,\ell }$. Let $v \in \partial J_{n,\ell }$, so that $(v,\phi _n(v)) \in \partial _{d-2}H_{n,\ell }$, By Eqs. 58 and 60,

$$\begin{aligned} |\phi _n(v)- \phi _n(u) | \le |\phi (v) -\phi (u)| + 4 K n^{18 {\zeta }} r_n^2 \le (1/9)\Vert v-u\Vert + 4 K n^{18 {\zeta }} r_n^2. \end{aligned}$$

Since $y \in I^+_{n,i}$ we have $\Vert y - (v,\phi _n(v))\Vert \ge \,\textrm{dist}(y,\partial H_{n,\ell }) \ge n^{4 {\zeta }} r_n$, so that

$$\begin{aligned} n^{4 {\zeta }} r_n \le \Vert u-v\Vert + |\phi _n(u) - \phi _n(v)| \le (10/9)\Vert u-v\Vert + 4Kn^{18 {\zeta }} r_n^2, \end{aligned}$$

and hence provided *n* is large enough, $\Vert u-v\Vert \ge n^{4 {\zeta }} r_n/2$, so that writing $y':= (u',\phi _n(u'))$ we have

$$ \Vert y- y' \Vert \ge \Vert u- u'\Vert \ge \,\textrm{dist}(u,\partial J_{n,\ell }) \ge n^{4{\zeta }} r_n/2. $$

But also $\Vert y-y'\Vert \le \Vert y-x\Vert + \Vert y'-x\Vert \le 2 n^{3 {\zeta }} r_n$, and we have our contradiction. $\square$

Denote the union of the boundaries (relative to $\mathbb {R}^{d-1} \times \{0\}$) of the lower faces of the blocks making up the strip/slab ${\textsf{S}}_n$, by $C_n^0$, and the $(9 n^{\zeta }r_n)$-neighbourhood in $\mathbb {H}$ of this region by $C_n$ (the *C* can be viewed as standing for ‘corner region’), i.e.

72 $$\begin{aligned} C_n^0 := \cup _{i=1}^{\lambda (n)} \theta _{n,i} (\partial I^+_{n,i}), ~~~~~~~ C_n := ( C_n^0 \oplus B(o,9 n^{\zeta }r_n) ) \cap \mathbb {H}. \end{aligned}$$

Here $\partial I^+_{n,i}$ denotes the relative boundary of $I^+_{n,i}$ (relative to the face $H_{n,j}$ containing $I^+_{n,i}$).

The next lemma says that the corner region $C_n$ is covered with high probability. It is needed because locations in $C_n$ lie near the boundaries of the blocks assembled to make the induced coverage process, so that coverage of these locations in the induced coverage process does not necessarily correspond to coverage of their pre-images in the original coverage process.

#### Lemma 4.18

It is the case that $\lim _{n \rightarrow \infty } \mathbb {P}[ F_n(C_n ,\mathcal{U}'_{n,\mathbb {H}}) ] =1$.

#### Proof

For each of the $(d-1)$-dimensional hypercubes $\theta _{n,i}(I_{n,i}^+), 1 \le i \le \lambda (n)$, the number of balls of radius $n^{\zeta }r_n$ required to cover the boundary is $O((n^{3{\zeta }}/n^{\zeta })^{d-2})$. Also $\lambda (n) = O((n^{3{\zeta }}r_n)^{1-d})$, so we can take points $x_{n,1}, \ldots , x_{n, m_n} \in L_n$, with $m_n = O(n^{-{\zeta }-d {\zeta }} r_n^{1-d})$, such that $C_n^0 \subset \cup _{i=1}^{m_n} B(x_{n,i}, n^{\zeta }r_n)$. Then $C_n \subset \cup _{i=1}^{m_n} B(x_{n,i},10 n^{\zeta }r_n)$, and $B(x_i,10 n^{\zeta }r_n)$ can be covered by $O(n^{d {\zeta }})$ balls of radius $r_n$. Hence by Eq. 64 from Lemma 4.14, taking $\varepsilon = {\zeta }/2$, we obtain the estimate

$$ \mathbb {P}[ F_n(C_n , \mathcal{U}'_{n,\mathbb {H}}) ^c] = O(n^{d{\zeta }} n^{-{\zeta }-d {\zeta }} r_n^{1-d} n^{\varepsilon - (d-1)/d}) = O(n^{- {\zeta }/2}), $$

which tends to zero. $\square$

#### Lemma 4.19

(Limiting coverage probabilities for the induced coverage process) With $c_{d,k,Y}$ given at Eq. 62,

73 $$\begin{aligned} \lim _{n \rightarrow \infty } \mathbb {P}[F_n({\textsf{S}}_n, \tilde{\mathcal{U}}'_{n,\mathbb {H}})] = \lim _{n \rightarrow \infty } \mathbb {P}[F_n(L_n, \tilde{\mathcal{U}}'_{n,\mathbb {H}})] = \exp (- c_{d,k,Y} |\Gamma | e^{- \beta /2} ) . \end{aligned}$$

#### Proof

The second equality of Eq. 73 is easily obtained using Eq. 37 from Lemma 4.4, together with Lemma 4.2.

Recall that $L_n = D_n \times \{0\}$. Also $\partial L_n \subset C_n^0$, so that $(\partial D_n \oplus B_{(d-1)}(o,n^{\zeta }r_n)) \times [0,2 n^{\zeta }r_n] \subset C_n$, and therefore by Eq. 44 from Lemma 4.4,

$$\begin{aligned} \mathbb {P}[( F_n(L_n, \tilde{\mathcal{U}}'_{n,\mathbb {H}}) \setminus F_n({\textsf{S}}_n, \tilde{\mathcal{U}}'_{n,\mathbb {H}}) ) \cap F_n(C_n, \tilde{\mathcal{U}}'_{n,\mathbb {H}}) ] \rightarrow 0. \end{aligned}$$

Therefore using also Lemma 4.18 shows that $\mathbb {P}[ F_n(L_n, \tilde{\mathcal{U}}'_{n,\mathbb {H}}) \setminus F_n({\textsf{S}}_n, \tilde{\mathcal{U}}'_{n,\mathbb {H}}) ] \rightarrow 0$, and this gives us the rest of Eq. 73. $\square$

#### Proof of Proposition 4.11

We shall approximate the event $F_n (W^{*}_n, \mathcal{U}'_{n,W_n})$ by events $F_n(L_n, \tilde{\mathcal{U}}'_{n,\mathbb {H}})$ and $F_n({\textsf{S}}_n,\tilde{\mathcal{U}}'_{n,\mathbb {H}})$, and apply Lemma 4.19. The sets $Q_n^+$, $P_n^+$ and ${\textsf{S}}_n$ and were defined at Eqs. 66, 68 and 70 respectively.

Suppose $F_n(Q^+_n \cup P_n^+, \mathcal{U}'_{n,W_n}) \setminus F_n(W_n^*, \mathcal{U}'_{n,W_n})$ occurs, and choose $x \in W_n^{*} \setminus (Q_n^+ \cup P_n^+) \setminus Z_n(\mathcal{U}'_{n,W_n})$. Let $y \in \Gamma _n$ with $\Vert y-x\Vert = \,\textrm{dist}(x,\Gamma _n)$. Then $\Vert y-x\Vert \le 2 n^{\zeta }r_n$, and since $x \notin Q_n^+$, we have $\,\textrm{dist}(x, \partial _{d-2} \Gamma _n) \ge 8d n^{4{\zeta }} r_n$, and hence $\,\textrm{dist}(y,\partial _{d-2}\Gamma _n) \ge 8dn^{4{\zeta }}r_n - 2n^{\zeta }r_n \ge 3 n^{4{\zeta }}r_n$, provided *n* is large enough. Therefore *y* lies in the interior of the face $H_{n,i}$ for some *i* and $x-y$ is perpendicular to $H_{n,i}$ (if $y \ne x$). Also, since $x \notin P_n^+$, $\,\textrm{dist}(x,P_n) \ge 9 n^{{\zeta }} r_n$, so $\,\textrm{dist}(y,P_n) \ge 7 n^{{\zeta }} r_n$. Therefore $y \in I_{n,j}$ for some *j*, and *x* lies in the block $S_{n,j}$. Hence $B(\theta _{n,j}(x),n^{\zeta }r_n) \cap \mathbb {H}\subset \theta _{n,j}(S^+_{n,j})$, and hence by Eq. 71,

$$ \#(\{ (z,s) \in \tilde{\mathcal{U}}'_{n,\mathbb {H}}: \theta _{n,j}(x) \in B(z,r_ns)\}) = \#(\{ (w,t) \in \mathcal{U}'_{n,W_n}: x \in B(w,r_nt)\}) < k, $$

so the event $F_n({\textsf{S}}_n, \tilde{\mathcal{U}}'_{n,\mathbb {H}})$ does not occur. Hence

$$ F_n({\textsf{S}}_n, \tilde{\mathcal{U}}'_{n,\mathbb {H}}) \setminus F_n(W_n^*, \mathcal{U}'_{n,W_n}) \subset F_n(Q^+_n \cup P^+_n, \mathcal{U}'_{n,W_n})^c, $$

so by Lemmas 4.15 and 4.16, $\mathbb {P}[ F_n({\textsf{S}}_n,\tilde{\mathcal{U}}'_{n,\mathbb {H}}) \setminus F_n (W^{*}_n , \mathcal{U}'_{n,W_n}) ] \rightarrow 0$, and hence using Eq. 73 we have

74 $$\begin{aligned} \liminf _{n \rightarrow \infty } \mathbb {P}[F_n(W_n^*,\mathcal{U}'_{n,W_n})] \ge \exp (-c_{d,k,Y} |\Gamma |e^{-\beta /2}). \end{aligned}$$

Suppose $$F_n (W^{*}_n , \mathcal{U}'_{n,W_n}) \setminus F_n(L_n,\tilde{\mathcal{U}}'_{n,\mathbb {H}})$$ occurs, and choose $$ y \in L_n \setminus Z_n(\tilde{\mathcal{U}}'_{n,\mathbb {H}})$$. Take $$i \in \{1,\ldots ,\lambda (n)\}$$ such that $$y \in \theta _{n,i}(I_{n,i}^+)$$. Then $$ \,\textrm{dist}( y, \theta _{n,i} (\partial I_{n,i})) \le n^{\zeta }r_n $$, since otherwise $$\theta _{n,i}^{-1}(y)$$ would be a location in $$A^{**}_n \setminus Z_n( \mathcal{U}'_{n,W_n})$$. Thus $$y \in C_n$$ by Eq. 72, and therefore using Lemma 4.18 yields that

$$ \mathbb {P}[ F_n(W^{*}_n, \mathcal{U}'_{n,W_n}) \setminus F_n(L_n,\tilde{\mathcal{U}}'_{n,\mathbb {H}}) ] \le \mathbb {P}[ F_n(C_n ,\mathcal{U}'_{n,W_n})^c ] \rightarrow 0 . $$

Combining this with Eq. 73 and Eq. 74 completes the proof. $\square$

### Proof of Theorem 2.10

Having obtained the limiting probability of covering a polytopal region approximating a part of *A* near the boundary and contained in a single chart, we shall now complete the proof of Theorem 2.10 by the following steps. First we shall show that error from the polytopal approximation vanishes, and then we put together finitely many regions of *A*, each of which is contained in a single chart, to get the limiting probability of covering the whole of *A*.

Proposition 4.11 gives the limiting probability of coverage of a polytopal approximation to a region near part of $\partial A$. The next two lemmas show that $\mathbb {P}[F_n(W^{*}_n, \mathcal{U}'_{n,W_n})]$ approximates $\mathbb {P}[F_n(A^*_n,\mathcal{U}'_{n,A_n})]$ (recall the definitions at Eq. 61). From this we can deduce that we get the same limiting probability even after dispensing with the polytopal approximation.

#### Lemma 4.20

Let $E^{(1)}_n:= F_n( W_n^* , \mathcal{U}'_{n,W_n}) \setminus F_n(A^*_n,\mathcal{U}'_{n,A_n})$. Then $\mathbb {P}[E^{(1)}_n] \rightarrow 0$ as $n \rightarrow \infty$.

#### Proof

Let $\varepsilon \in (0, (1/(2d)) - 9 {\zeta })$. Suppose $E^{(1)}_n \cap F_n(Q_n^+, \mathcal{U}'_{n,A_n})$ occurs. Then since $\mathcal{U}'_{n,W_n} \subset \mathcal{U}'_{n,A_n}$, $\mathbb {R}^d \setminus Z_n(\mathcal{U}'_{n,A_n})$ intersects with $A^*_n \setminus W^{*}_n$, and therefore by Eq. 60, includes locations distant at most $2K n^{18 {\zeta }}r_n^2$ from $\Gamma _n$. Also $\Gamma _n \subset Z_n (\mathcal{U}'_{n,A_n})$, since $\Gamma _n \subset W^{*}_n$.

Pick a location $x \in \overline{A_n^* \setminus Z_n(\mathcal{U}'_{n,A_n}) }$ of minimal distance from $\Gamma _n$. Then $x \notin Q_n^+$, so the nearest point in $\Gamma _n$ to *x* lies in the interior of $H_{n,i}$ for some *i*. We claim that *x* lies at the intersection of the boundaries of *d* of the balls making up $Z_n(\mathcal{U}'_{n,A_n})$; this is proved similarly to the similar claim concerning *w* in the proof of Lemma 4.4. Moreover, *x* lies in at most $k-1$ of the other balls making up $Z_n(\mathcal{U}'_{n,A_n})$. Also *x* does not lie in the interior of $W_n^*$.

Thus if $$E^{(1)}_n \cap F_n(Q_n^+, \mathcal{U}'_{n,A_n})$$ occurs, there must exist *d* points $$(x_1,s_1),(x_2,s_2),\ldots ,$$ $$ (x_d,s_d)$$ of $$\mathcal{U}_{n,A_n} $$ such that $$\cap _{i=1}^d \partial B(x_i,r_ns_i)$$ includes a point in $$A_n^*$$ but outside the interior of $$W_n^*$$, within distance $$2Kn^{18 {\zeta }} r_n^2$$ of $$\Gamma _n$$ and in $$B(x,r_n s)$$ for at most $$k-1$$ of the other points (*x*, *s*) of $$\mathcal{U}_{n,A_n}$$. Hence by Markov’s inequality and the Mecke formula, we obtain that

75 $$\begin{aligned} \mathbb {P}[E^{(1)}_n \cap F_n(Q_n^+ , \mathcal{U}'_{n,A_n})] \le I_{n,1} + I_{n,2} \end{aligned}$$

where, taking $Y_1,\ldots , Y_d$ to be independent random variables with the distribution of *Y*, and writing $f_{n,A}(x)$ for $\mathbb {P}[x \in Z_n(\mathcal{U}'_{n,A})]$ for all $x \in \mathbb {R}^d$, and recalling the definition of $q_n(\cdot )$ at Eq. 35, we set

$$\begin{aligned} I_{n,1} : =&n^d \int _{\mathbb {R}^d} \cdots \int _{\mathbb {R}^d} \mathbb {E}[h_n((x_1,Y_1),(x_1+y_2,Y_2),\ldots ,(x_1+y_d,Y_d))\\&\times \textbf{1} \{q_n((x_1,Y_1), (x_1+y_2,Y_2), \ldots ,(x_1+y_d,Y_d)) \in A_n^* \cap (\Gamma _n \oplus B(o,2Kn^{18{\zeta }} r_n^2) ) \}\\&\times (1-f_{n,A}(q_n( (x_1,Y_1), (x_1+y_2,Y_2),\ldots ,(x_1+y_d,Y_d)) ))] dy_d \cdots dy_2 dx_1, \end{aligned}$$

and $I_{n,2}$ is defined similarly with $p_n(\cdot )$ replacing $q_n(\cdot )$. Then changing variables $y_i \mapsto r_n^{-1} y_i$ we have

$$\begin{aligned} I_{n,1} =&n^d r_n^{d(d-1)} \mathbb {E}\Big [ \int _{\mathbb {R}^d} \cdots \int _{\mathbb {R}^d} h_n((o,Y_1),(r_n y_2,Y_2),\ldots ,(r_n y_d,Y_d))\\&\times \textbf{1} \{x_1 + q_n((o,Y_1), (r_n y_2,Y_2), \ldots ,(r_ny_d Y_d)) \in A_n^* \cap (\Gamma _n \oplus B(o,2Kn^{18{\zeta }} r_n^2)) \}\\&\times (1-f_{n,A}(x_1 + q_n( (o,Y_1), (r_ny_2,Y_2),\ldots , (r_ny_d,Y_d)) ))dy_d \cdots dy_2 dx_1 \Big ]. \end{aligned}$$

By Lemma 4.14, the last factor of $1-f_{n,A}(\cdot )$ is $O(n^{\varepsilon -1+1/d})$ whenever the indicator function in the previous factor is 1. Also using Fubini’s theorem we can take the integral over $x_1$ inside all the other integrals and integrate it out, getting a factor of $O(n^{18{\zeta }} r_n^2)$. Thus we obtain for a suitable constant *c* that

$$\begin{aligned} I_{n,1} \le c n^d r_n^{d(d-1)} n^{18 {\zeta }+ \varepsilon -1+ 1/d} r_n^2 \mathbb {E}\Big [ \int _{\mathbb {R}^d} \cdots \int _{\mathbb {R}^d} h((o,r_nY_1),(r_ny_2,r_nY_2),\ldots ,(r_ny_d,r_nY_d)) \\ dy_d \cdots dy_2 \Big ] \\ = c (nr_n^d)^{d-1 + 2/d} n^{18{\zeta }+\varepsilon -1/d} \mathbb {E}\Big [ \int _{\mathbb {R}^d} \cdots \int _{\mathbb {R}^d} h((o,Y_1),(y_2,Y_2),\ldots ,(y_d,Y_d)) dy_d \cdots dy_2 \Big ]. \end{aligned}$$

In the last line the expectation is finite by Lemma 4.3 and our moment condition on *Y*. Also $(nr_n^d)^d = O(n^\varepsilon )$ by Eq. 17. Thus $I_{n,1}= O(n^{2 \varepsilon + 18 {\zeta }- 1/d})$ so $I_{n,1} \rightarrow 0$ as $n\rightarrow \infty$, and by an identical argument the same holds for $I_{n,2}$. Also $\mathbb {P}[ F_n(Q_n^+, \mathcal{U}'_{n,A_n})] \rightarrow 1$ by Lemma 4.15, so using Eq. 75 we obtain that $\mathbb {P}[E^{(1)}_n ] \rightarrow 0$, as required. $\square$

#### Lemma 4.21

Let $$E^{(2)}_n:= F_n(A_n^*, \mathcal{U}'_{n,A_n}) \setminus F_n(W_n^*, \mathcal{U}'_{n,W_n})$$. Then $$\lim _{n \rightarrow \infty } \mathbb {P}[ E^{(2)}_n]=0$$.

#### Proof

If the event $E^{(2)}_n$ occurs, then since $W^{*}_n \subset A_n^*$, the set $W^{*}_n \cap Z_n(\mathcal{U}'_{n,A_n}) \setminus Z_n(\mathcal{U}'_{n,W_n})$ is nonempty. Hence there exists $(x,s) \in \mathcal{U}'_{n,A_n} \setminus \mathcal{U}'_{n,W_n}$ with $B(x,r_n s) \cap W^{*}_n \setminus Z_n(\mathcal{U}'_{n,W_n}) \ne \emptyset$. Therefore

76 $$\begin{aligned} E^{(2)}_n \subset F_n( Z_n(\mathcal{U}'_{n,A_n} \setminus \mathcal{U}'_{n,W_n}) \cap W_n^* , \mathcal{U}'_{n,W_n})^c . \end{aligned}$$

Let $\varepsilon \in (0,(1/(2d))- (9 +d/2){\zeta })$. Let $\mathcal{Q}_n: = \mathcal{U}'_{n,A_n} \setminus \mathcal{U}'_{n,W_n}$. Then $\mathcal{U}'_{n,W_n}$ and $\mathcal{Q}_n$ are independent Poisson processes with intensity measures $n \textrm{Leb}_d \otimes \mu _Y$ in $W_n \times [0,n^{\zeta }]$, $(A_n \setminus W_n) \times [0,n^{\zeta }]$ respectively. By Lemma 4.14 and the union bound, there is a constant *c* such that for any $m \in \mathbb {N}$ and any set of *m* points $(x_1,t_1),\ldots ,(x_m,t_m)$ in $\mathbb {R}^d \times [0,n^{\zeta }]$, we have

$$\begin{aligned} \mathbb {P}\left[ F_n( \cup _{i=1}^m B(x_i,r_n t_i) \cap W_n^* ,\mathcal{U}'_{n,W_n} )^c \right] \le c m n^{d{\zeta }} n^{\varepsilon - (d-1)/d} . \end{aligned}$$

Let $N_n:= \mathcal{Q}_n(\mathbb {R}^d \times [0,n^{\zeta }])$. By Lemma 4.12(a), $\mathbb {E}[ N_n] = O(n^{1+ 18 {\zeta }} r_n^2 )$, so that by conditioning on $\mathcal{Q}_n$ we have

$$\begin{aligned} \mathbb {P}[ F_n( \cup _{(x,t) \in \mathcal{Q}_n } B(x,r_nt) \cap W_n^* , \mathcal{U}'_{n,W_n} )^c]&\le c n^{\varepsilon + d {\zeta }- 1 +1/d} \mathbb {E}[N_n] \\&= O( n^{ (18 +d) {\zeta }+ 2 \varepsilon - 1/d }), \end{aligned}$$

which tends to zero by the choice of $\varepsilon$. Hence by Eq. 76, $\mathbb {P}[E^{(2)}_n ] \rightarrow 0$. $\square$

To complete the proof of Theorem 2.3, we shall break $\partial A$ into finitely many pieces, with each piece contained in a single chart. We would like to write the probability that all of $\partial A$ is covered as the product of probabilities for each piece, but to achieve the independence needed for this, we need to remove a region near the boundary of each piece. By separate estimates we can show the removed regions are covered with high probability, and this is the content of the next lemma.

With $\Gamma$ and $\partial \Gamma$ as in Section 4.4, define the sets ${\Delta }_n := \partial \Gamma \oplus B(o, n^{29{\zeta }}r_n)$ and ${\Delta }_n^{+} := \partial \Gamma \oplus B(o, n^{49 {\zeta }}r_n).$

#### Lemma 4.22

It is the case that $\lim _{n \rightarrow \infty } F_n( {\Delta }_n^{+} \cap A, \mathcal{U}'_ {n,A}) =1$.

#### Proof

Let $\varepsilon \in (0,(1/d) - 98{\zeta })$. Since we assume $\kappa (\partial \Gamma ,r)= O(r^{2-d})$ as $r \downarrow 0$, for each *n* we can take $x_{n,1},\ldots ,x_{n,k(n)} \in \mathbb {R}^d$ with $\partial \Gamma \subset \cup _{i=1}^{k(n)} B(x_{n,i},n^{49{\zeta }}r_n)$, and with $k(n) = O((n^{49 {\zeta }} r_n)^{2-d})$. Then ${\Delta }_n^{+} \subset \cup _{i=1}^{k(n)} B(x_{n,i},2n^{49{\zeta }}r_n)$. For each $i \in \{1,\ldots , k(n)\}$, we can cover the ball $B(x_{n,i},2 n^{49 {\zeta }}r_n)$ with $O(n^{49d{\zeta }})$ smaller balls of radius $r_n$. Then we end up with balls of radius $r_n$, denoted $B_{n,1},\ldots ,B_{n,m(n)}$ say, such that ${\Delta }_n^{+} \subset \cup _{i=1}^{m(n)} B_{n,i}$ and $m(n) = O(r_n^{2-d} n^{49{\zeta }(3-d)}) = O(r_n^{2-d} n^{98{\zeta }})$. By Eq. 65 from Lemma 4.14, and the union bound,

$$\begin{aligned} \mathbb {P}[ \cup _{i=1}^{m(n)} ( F_{n}(B_{n,i} \cap A, \mathcal{U}'_{n,A})^c)] = O( r_n^{2-d} n^{98 {\zeta }+ \varepsilon - 1 + 1/d}) = O( n^{ 98 {\zeta }+ \varepsilon - 1/d} ), \end{aligned}$$

which tends to zero. $\square$

Given $n >0$, define the sets $\Gamma ^{(n^{29 {\zeta }}r_n)}:= \Gamma \setminus {\Delta }_n$ and

$$ \Gamma ^{(n^{29 {\zeta }}r_n)}_{n^{\zeta }r_n} := (\Gamma ^{(n^{29 {\zeta }}r_n)} \oplus B(o, n^{\zeta }r_n)) \cap A; ~~~~~ \Gamma _{n^{\zeta }r_n} := (\Gamma \oplus B(o,n^{\zeta }r_n)) \cap A, $$

and define the event $F_n^\Gamma := F_n(\Gamma ^{(n^{29 {\zeta }}r_n)}_{n^{\zeta }r_n} , \mathcal{U}'_{n,A} )$.

Note that the definition of $F_n^\Gamma$ does not depend on the choice of chart. This fact will be needed for the last stage of the proof of Theorem 2.3. Lemma 4.25 below shows that $\mathbb {P}[F_n^\Gamma ]$ is well approximated by $\mathbb {P}[F_n(A_n^*, \mathcal{U}'_{n,A_n})]$ and we have already determined the limiting behaviour of the latter. We prepare for the proof of Lemma 4.25 with two geometrical lemmas.

#### Lemma 4.23

For all large enough *n*, it is the case that $\Gamma _{n^{\zeta }r_n}^{(n^{29 {\zeta }}r_n)} \subset A_n^*$.

#### Proof

Let $x \in \Gamma ^{(n^{29{\zeta }}r_n)}_{n^{\zeta }r_n}$, and take $y \in \Gamma ^{(n^{29{\zeta }}r_n)}$ with $\Vert x-y\Vert \le n^{\zeta }r_n$. Writing $y = (u,\phi (u))$ with $u \in U$, we claim that $\,\textrm{dist}(u, \partial U) \ge (1/2)n^{29 {\zeta }}r_n$. Indeed, if we had $\,\textrm{dist}(u, \partial U) < (1/2)n^{29 {\zeta }}r_n$, then we could take $w \in \partial U$ with $\Vert u -w \Vert < (1/2)n^{29 {\zeta }}r_n$. Then $(w,\phi (w)) \in \partial \Gamma$ and by Eq. 58, $|\phi (w) - \phi (u)| \le (1/4) n^{29 {\zeta }}r_n$, so

$$ \Vert (u,\phi (u)) - (w,\phi (w)) \Vert \le \Vert u-w\Vert + |\phi (u)-\phi (w)| \le (3/4)n^{29 {\zeta }}r_n, $$

contradicting the assumption that $y \in \Gamma ^{(n^{29 {\zeta }}r_n)}$, so the claim is justified.

Writing $x = (v,s)$ with $v \in \mathbb {R}^{d-1}$, and $s \in \mathbb {R}$, we have $\Vert v-u\Vert \le \Vert x-y\Vert \le n^{\zeta }r_n$, so $\,\textrm{dist}(v, \partial U) \ge (1/2)n^{29{\zeta }}r_n - n^{\zeta }r_n$, and hence $v \in U_n^-$, provided *n* is big enough ($U_n^-$ was defined shortly after Eq. 59.) Also $|\phi (v) - \phi (u)| \le n^{\zeta }r_n/4$ by Eq. 58, so $|\phi _n(v) - \phi (u)| \le n^{\zeta }r_n/2$, provided *n* is big enough, by Eq. 60. Also $|s - \phi (u)| \le \Vert x-y\Vert \le n^{\zeta }r_n$, so $|s - \phi _n(v)| \le (3/2) n^{\zeta }r_n.$ Therefore $x \in A^*_n$ by Eq. 61. $\square$

#### Lemma 4.24

For all large enough *n*, we have (a) $[A_n^* \oplus B(o,4 n^{\zeta }r_n)] \cap A \subset A_n$, and (b) $[ A_n^* \oplus B(o,4 n^{\zeta }r_n)] \cap \partial A \subset \Gamma$, and (c) $[ \Gamma _{n^{\zeta }r_n}^{(n^{29{\zeta }}r_n)} \oplus B(o,4 n^{\zeta }r_n)] \cap \partial A \subset \Gamma$.

#### Proof

Let $x \in A_n^*$. Write $x = (u,z)$ with $u \in U_n^-$ and $\phi _n(u) - 3 n^{\zeta }r_n/2 \le z \le \phi (u)$.

Let $y \in B(x,4 n^{\zeta }r_n) \cap A$, and write $y = (v,s)$ with $v \in \mathbb {R}^{d-1}$ and $s \in \mathbb {R}$. Then $\Vert v-u\Vert \le 4 n^{\zeta }r_n$ so provided *n* is big enough, $v \in U_n$. Also $|s-z| \le 4 n^{\zeta }r_n$, and $|\phi (v) - \phi (u)| \le n^{\zeta }r_n$ by Eq. 58, so

$$ |s - \phi (v)| \le |s - z| + |z - \phi (u)| + |\phi (u)- \phi (v)| \le 4 n^{\zeta }r_n + 2 n^{\zeta }r_n + n^{\zeta }r_n, $$

and since $y \in A$, by Eqs. 56 and 57 we must have $0 \le s \le \phi (v)$, provided *n* is big enough. Therefore $y = (v,s) \in A_n$, which gives us (a).

If also $y \in \partial A$, then $\phi (v)=s$, so $y \in \Gamma$. Hence we have part (b). Then by Lemma 4.23 we also have part (c). $\square$

#### Lemma 4.25

It is the case that $\mathbb {P}[F_n^\Gamma \triangle F_n(A_n^*, \mathcal{U}'_{n,A_n})] \rightarrow 0$ as $n \rightarrow \infty$.

#### Proof

Since $\Gamma _{n^{\zeta }r_n}^{(n^{29{\zeta }}r_n)} \subset A_n^*$ by Lemma 4.23, and moreover $\mathcal{U}'_{n,A_n} \subset \mathcal{U}'_{n,A}$, it follows that $F_n(A_n^*,\mathcal{U}'_{n,A_n}) \subset F_n(\Gamma ^{(n^{29{\zeta }}r_n)}_{n^{\zeta }r_n} , \mathcal{U}'_{n,A}) = F_n^\Gamma$. Therefore it suffices to prove that

77 $$\begin{aligned} \mathbb {P}[F_n(\Gamma ^{(n^{29\xi }r_n)}_{n^{\zeta }r_n} , \mathcal{U}'_{n,A}) \setminus F_n(A_n^*,\mathcal{U}'_{n,A_n}) ] \rightarrow 0. \end{aligned}$$

Let $\varepsilon >0$. Suppose event $F_n(\Gamma ^{(n^{29{\zeta }}r_n)}_{n^{\zeta }r_n} , \mathcal{U}'_{n,A}) \cap F_n( {\Delta }_n^{+} \cap A, \mathcal{U}'_{n,A}) \setminus F_n(A_n^*,\mathcal{U}'_{n,A_n})$ occurs. Choose $x \in A_n^* \setminus Z_n(\mathcal{U}'_{n,A_n})$. Then by Lemma 4.24(a), $B(x,n^{\zeta }r_n) \cap A \subset A_n$. Hence $\mathcal{U}'_{n,A} \cap ( B(x, n^{\zeta }r_n) \times \mathbb {R}_+) \subset \mathcal{U}'_{n,A_n}$, and therefore $x \notin Z_n(\mathcal{U}'_{n,A})$.

Since we are assuming $$ F_n(\Gamma ^{(n^{29{\zeta }}r_n)}_{n^{\zeta }r_n} , \mathcal{U}'_{n,A}) $$ occurs, we therefore have $$\,\textrm{dist}(x, \Gamma ^{(n^{29 {\zeta }}r_n)} )$$$$> n^{\zeta }r_n $$. Since we also assume $$F_n({\Delta }_n^{+} \cap A,\mathcal{U}'_{n,A})$$, we also have $$\,\textrm{dist}(x, \partial \Gamma ) \ge n^{49 {\zeta }}r_n$$ and therefore $$\,\textrm{dist}(x,{\Delta }_n) = \,\textrm{dist}(x, \partial \Gamma \oplus B(o,n^{29{\zeta }}r_n)) \ge n^{29{\zeta }} r_n$$. Hence

$$ \,\textrm{dist}(x,\Gamma ) \ge \min (\,\textrm{dist}(x, \Gamma ^{(n^{29{\zeta }}r_n)}), \,\textrm{dist}(x, \partial \Gamma \oplus B(o,n^{29 {\zeta }}r_n))) > n^{\zeta }r_n. $$

Moreover, by Lemma 4.24(b), $\,\textrm{dist}(x, (\partial A) \setminus \Gamma ) > n^{\zeta }r_n$. Thus $\,\textrm{dist}(x, \partial A) > n^{\zeta }r_n$. Moreover, $\,\textrm{dist}(x,\partial A) \le \,\textrm{dist}(x,\Gamma ) \le 2 n^{\zeta }r_n$ because $x \in A_n^*$, and therefore $x \notin A^{[\varepsilon ]}$ (provided *n* is large enough) since $\overline{A^{[\varepsilon ]}}$ is compact and contained in $A^o$ (the set $A^{[\varepsilon ]}$ was defined in Section 2.) Therefore the event $F_n(A^{( n^{\zeta }r_n)} \setminus A^{[\varepsilon ]},\mathcal{U}'_{n,A})^c$ occurs. Thus, for large enough *n* we have the event inclusion

78 $$\begin{aligned} F_n(\Gamma ^{(n^{29{\zeta }}r_n)}_{r_n} , \mathcal{U}'_{n,A}) \cap F_n( {\Delta }_n^{+} \cap A, \mathcal{U}'_{n,A}) \setminus F_n(A_n^*,\mathcal{U}'_{n,A_n}) \subset F_n( A^{(n^{\zeta }r_n)} \setminus A^{[\varepsilon ]} ,\mathcal{U}'_{n,A})^c . \end{aligned}$$

By Eq. 17,

79 $$\begin{aligned} \lim _{n \rightarrow \infty } ( \omega _d \mathbb {E}[Y^d] n r_n^{d} - \log n - (d+k-2) \log \log n ) = {\left\{ \begin{array}{ll} \beta & \textrm{if} ~ d=2 ,k=1\\ +\infty & \mathrm{otherwise.} \end{array}\right. } \end{aligned}$$

Hence by Lemma 3.1 applied to the set $A \setminus A^{[\varepsilon ]}$, and Lemma 4.2 we have that

80 $$\begin{aligned} \liminf _{n \rightarrow \infty } \mathbb {P}[ F_n( A^{(n^{\zeta }r_n)} \setminus A^{[\varepsilon ]} ,\mathcal{U}'_{n,A}) ] = \liminf _{n \rightarrow \infty } \mathbb {P}[ F_n( A^{(n^{\zeta }r_n)} \setminus A^{[\varepsilon ]} ,\mathcal{U}'_{n,\mathbb {R}^d}) ] \nonumber \\ \ge \liminf _{n \rightarrow \infty } \mathbb {P}[ F_n( A \setminus A^{[\varepsilon ]} ,\mathcal{U}_{n,\mathbb {R}^d}) ] \nonumber \\ = {\left\{ \begin{array}{ll} \exp \Big (- c_d \Big ( \frac{(\mathbb {E}[Y^{d-1}])^d}{ (\mathbb {E}[Y^d])^{d-1}} \Big ) |A \setminus A^{[\varepsilon ]} |e^{- \beta } \Big ) & \mathrm{if~} d=2 , k=1 \\ 1 & \mathrm{otherwise.} \end{array}\right. } \end{aligned}$$

Therefore since $\varepsilon$ can be arbitrarily small and $|A \setminus A^{[\varepsilon ]} | \rightarrow 0$ as $\varepsilon \downarrow 0$, the event displayed on the left hand side of Eq. 78 has probability tending to zero. Then using Lemma 4.22, we have Eq. 77, which completes the proof. $\square$

#### Corollary 4.26

It is the case that $\lim _{n \rightarrow \infty } \mathbb {P}[F_n^\Gamma ] = \exp (- c_{d,k,Y} |\Gamma | e^{- \beta /2} ).$

#### Proof

By Lemmas 4.20 and 4.21, $\mathbb {P}[ F_n(W^{*}_n, \mathcal{U}'_{n,W_n}) \triangle F_n(A_n^*, \mathcal{U}'_{n,A_n}) ] \rightarrow 0$. Then by Lemma 4.25, $\mathbb {P}[ F_n^\Gamma \triangle F_n(W^{*}_n, \mathcal{U}'_{n,W_n}) ] \rightarrow 0$, and now the result follows by Proposition 4.11. $\square$

#### Proof of Theorem 2.10

Let $x_1,\ldots ,x_J$ and $r(x_1),\ldots ,r(x_J)$ be as described at Eq. 55. Set $\Gamma _1:= B(x_1,r(x_1) ) \cap \partial A$, and for $j =2,\ldots , J$, let

$$ \Gamma _j := \overline{ B(x_j,r(x_j) ) \cap \partial A \setminus \cup _{i=1}^{j-1} B(x_i,r(x_i))}, $$

and $\partial \Gamma _i:= \Gamma _i \cap \overline{\partial A \setminus \Gamma _i}$. Then $\Gamma _1,\ldots ,\Gamma _J$ comprise a finite collection of closed sets in $\partial A$ with disjoint interiors, each of which satisfies $\kappa (\partial \Gamma _i,r) = O(r^{2-d})$ as $r \downarrow 0$, and is contained in a single chart $B(x_j,r(x_j))$, and with union $\partial A$. For $1 \le i \le J$, define $F_n^{\Gamma _i}$ analogously to $F_n^\Gamma$, that is, $F_n^{\Gamma _i} := F_n(\Gamma _{i,n^{{\zeta }} r_n}^{(n^{29{\zeta }}r_n)} , \mathcal{U}'_{n,A})$ with

$$ \Gamma _{i,n^{\zeta }r_n}^{(n^{29 {\zeta }}r_n)} := \left( \left[ \Gamma _i \setminus ((\partial \Gamma _i) \oplus B(o,n^{29 {\zeta }}r_n )) \right] \oplus B(o, n^{\zeta }r_n) \right) \cap A. $$

First we claim that the following event inclusion holds:

$$\begin{aligned} \cap _{i=1}^J F_n^{\Gamma _i} \cap F_n(A^{(n^{\zeta }r_n)}, \mathcal{U}'_{n,A}) \setminus F_n(A,\mathcal{U}'_{n,A}) \subset \left( \cap _{i=1}^J F_n([(\partial \Gamma _i) \oplus B(o,n^{49{\zeta }}r_n)] \cap A, \mathcal{U}'_{n,A}) \right) ^c. \end{aligned}$$

Indeed, suppose $\cap _{i=1}^J F_n^{\Gamma _i} \cap F_n(A^{(n^{\zeta }r_n)}, \mathcal{U}'_{n,A}) \setminus F_n(A,\mathcal{U}'_{n,A})$ occurs, and choose $x \in A \setminus Z_n(\mathcal{U}'_{n,A})$. Then $\,\textrm{dist}(x,\partial A) \le n^{\zeta }r_n$ since we assume $F_n(A^{(n^{\zeta }r_n)},\mathcal{U}'_{n,A})$ occurs. Then for some $i \in \{1,\ldots ,J\}$ and some $y \in \Gamma _i$ we have $\Vert x-y \Vert \le n^{\zeta }r_n$. Since we assume $F_n^{\Gamma _i}$ occurs, we have $x \notin \Gamma _{i,n^{\zeta }r_n}^{(n^{29{\zeta }}r_n)}$, and hence $\,\textrm{dist}(y, \partial \Gamma _i) \le n^{29{\zeta }}r_n$, so $\,\textrm{dist}(x, \partial \Gamma _i) < n^{49 {\zeta }}r_n$. Therefore $F_n([(\partial \Gamma _i) \oplus B(o, n^{49{\zeta }}r_n )] \cap A, \mathcal{U}'_{n,A})$ fails to occur, justifying the claim.

By the preceding claim and and the union bound,

$$\begin{aligned} \mathbb {P}[F_n(A,\mathcal{U}'_{n,A}) ]&\le \mathbb {P}[ \cap _{i=1}^J F_n^{\Gamma _i} \cap F_n(A^{(n^{\zeta }r_n)},\mathcal{U}'_{n,A}) ]\\&\le \mathbb {P}[F_n(A,\mathcal{U}'_{n,A}) ] + \sum _{i=1}^J \mathbb {P}[ F_n([(\partial \Gamma _i) \oplus B(o,n^{49{\zeta }}r_n)] \cap A, \mathcal{U}'_{n,A})^c]. \end{aligned}$$

By Lemma 4.22, $\mathbb {P}[ F_n([(\partial \Gamma _i) \oplus B(o,n^{49{\zeta }}r_n)] \cap A, \mathcal{U}'_{n,A})] \rightarrow 1$ for each *i*. Therefore

81 $$\begin{aligned} \lim _{n \rightarrow \infty } \mathbb {P}[F_{n} (A,\mathcal{U}'_{n,A}) ] = \lim _{n \rightarrow \infty } \mathbb {P}[ \cap _{i=1}^J F_{n}^{\Gamma _i} \cap F_n(A^{(n^{\zeta }r_n)}, \mathcal{U}'_{n,A}) ], \end{aligned}$$

provided the last limit exists. By Corollary 4.26, we have for each *i* that

82 $$\begin{aligned} \lim _{t \rightarrow \infty } (\mathbb {P}[ F^{\Gamma _i}_n]) = \exp (- c_{d,k,Y}|\Gamma _i| e^{-\beta /2} ). \end{aligned}$$

Also, we claim that for large enough *n* the events $F_n^{\Gamma _1}$, ..., $F_n^{\Gamma _J}$ are mutually independent. Indeed, given distinct $i,j \in \{1,\ldots ,J\}$, if $x \in \Gamma _{i,n^{\zeta }r_n}^{(n^{29{\zeta }}r_n)}$ and $y \in \Gamma _{j,n^{\zeta }r_n}^{(n^{29{\zeta }}r_n)}$, then we can take $y' \in \Gamma _j \setminus ( \partial \Gamma _j \oplus B(o,n^{29{\zeta }}r_n))$ with $\Vert y'-y \Vert \le n^{\zeta }r_n$. If $\Vert x -y \Vert \le 3 n^{\zeta }r_n$ then by the triangle inequality $\Vert x - y'\Vert \le 4 n^{\zeta }r_n$, but since $y' \notin \Gamma _i$, this would contradict Lemma 4.24(c). Therefore $\Vert x - y\Vert > 3 n^{\zeta }r_n$, and hence the $n^{\zeta }r_n$-neighbourhoods of $\Gamma _{i,n^{\zeta }r_n}^{(n^{29{\zeta }}r_n)}$ and of $\Gamma _{j,n^{\zeta }r_n}^{(n^{29{\zeta }}r_n)}$ are disjoint. This gives us the independence claimed.

Now observe that $F_n(A^{(n^{\zeta }r_n)},\mathcal{U}'_{n,A}) \subset F_n(A^{(4n^{\zeta }r_n)},\mathcal{U}'_{n,A})$. We claim that

83 $$\begin{aligned} \mathbb {P}[ F_n(A^{(4n^{\zeta }r_n)},\mathcal{U}'_{n,A}) \setminus F_n(A^{(n^{\zeta }r_n)}, \mathcal{U}'_{n,A})] \rightarrow 0 ~~~ \textrm{as} ~ n \rightarrow \infty . \end{aligned}$$

Indeed, given $\varepsilon >0$, for large *n* the probability on the left side of Eq. 83 is bounded by $\mathbb {P}[ F_n(A^{(n^{\zeta }r_n)}\setminus A^{[\varepsilon ]},\mathcal{U}'_{n,A})^c]$, and by Eq. 80 the limsup of the latter probability can be made arbitrarily small by the choice of $\varepsilon$. Hence by Lemma 3.1 and Eq. 79, Lemma 4.2 and the fact that $c_2=1$,

84 $$\begin{aligned} \lim _{n \rightarrow \infty } \mathbb {P}[ F_n(A^{(4n^{\zeta }r_n)},\mathcal{U}'_{n,A}) ]&=\lim _{n \rightarrow \infty } \mathbb {P}[ F_n(A^{(n^{\zeta }r_n)},\mathcal{U}'_{n,A})] \nonumber \\&= \lim _{n \rightarrow \infty } \mathbb {P}[ F_n(A^{(n^{\zeta }r_n)},\mathcal{U}'_{n,\mathbb {R}^d})] \nonumber \\&= {\left\{ \begin{array}{ll} \exp \Big ( - \Big ( \frac{(\mathbb {E}[Y])^2}{ \mathbb {E}[Y^2]} \Big ) |A| e^{- \beta } \Big ) & \textrm{if} ~ d=2, k=1 \\ 1 & \textrm{otherwise} . \end{array}\right. } \end{aligned}$$

Moreover, by Eqs. 81 and 83,

85 $$\begin{aligned} \lim _{n \rightarrow \infty } \mathbb {P}[ F_n( A , \mathcal{U}'_{n,A}) ] = \lim _{n \rightarrow \infty } \mathbb {P}[ \cap _{i=1}^J F_n^{\Gamma _i} \cap F_n(A^{(4n^{\zeta }r_n)}, \mathcal{U}'_{n,A}) ], \end{aligned}$$

provided the last limit exists. However, the events in the right hand side of Eq. 85 are mutually independent, so using Eqs. 82, 62 and 84, we obtain that

$$\begin{aligned} \lim _{n \rightarrow \infty } \mathbb {P}[ F_{n}(A,\mathcal{U}'_{n,A})] = \exp \Big ( - c_{d,k} \Big (\frac{(\mathbb {E}[Y^{d-1}])^{d-1}}{(\mathbb {E}[Y^{d}])^{d-2+1/d}} \Big ) |\partial A| e^{-\beta /2} \\ - \Big ( \frac{\mathbb {E}[Y]^2}{\mathbb {E}[Y^2]} \Big ) |A| e^{-\beta } \textbf{1}_{\{(d,k)=(2,1)\}}\Big ). \end{aligned}$$

By Lemma 4.2, if we replace $\mathcal{U}'_{n,A}$ with $\mathcal{U}_{n,A}$ on the left, we get the same limit, i.e. Eq. 18 if $d \ge 3$ and Eq. 14 if $d=2$. $\square$

## Data Availability

The code and data used to generate Figs. 1 and 7 and the videos discussed in Section 2, as well as all the code and data used to create Fig. 2, are available on Github at the following https://github.com/frankiehiggs/johnson-mehl

## Appendix A: Confirming consistency with Chiu’s result

In this appendix we verify that our Proposition 2.1 is consistent with Chiu (1995), Theorem 4. The latter result is concerned with a quantity denoted $T_L$ in Chiu (1995) which is the same as our $\tilde{\tau }_L$, assuming we take $v= \gamma =1$ in Chiu (1995). Chiu takes $A = [0,1]^d$ and defines $c = c(L) = d(d+1) \log L - \log \omega _d$. Then in our notation, the result in Chiu (1995) says that for any $u \in \mathbb {R}$, as $L \rightarrow \infty$ we have

A1 $$\begin{aligned} \mathbb {P}\left[ c^{d/(d+1)} \omega _d^{1/(d+1)} \tilde{\tau }_L - c - \log \left( c^{1/(d+1)}\left( \frac{c+ \log c}{d+1} \right) ^{d-1} \right) \le u \right] \rightarrow F(u), \end{aligned}$$

with $F(u) = \exp (-c_d (d+1)^{d-1}d^{-d} e^{-u})$ (recall that our $c_d$ is the same as Chiu’s $\psi _d$ so *c* and $c_d$ are two different things here).

To verify that we can recover Eq. A1 from Proposition 2.1, note first that $c \rightarrow \infty$ as $L \rightarrow \infty$ and $\log c = \log \log L + \log (d(d+1)) + o(1)$. Also

$$\begin{aligned} \log \left( c^{1/(d+1)}\left( \frac{c+ \log c}{d+1} \right) ^{d-1} \right) = \log \left( c^{d^2/(d+1)} (d+1)^{1-d} \right) + o(1). \end{aligned}$$

Hence for some $z_L$ which tends to zero as $L \rightarrow \infty$, the left hand side of Eq. A1 equals

$$\begin{aligned} \mathbb {P}\left[ c^d \omega _d \tilde{\tau }_L^{d+1} \le \left( c+ \log (c^{d^2/(d+1)} (d+1)^{1-d} ) + u + z_L \right) ^{d+1} \right] . \end{aligned}$$

By binomial expansion, for some $y_L$ which tends to zero as $L \rightarrow \infty$, this probability equals

$$\begin{aligned}&\mathbb {P}\left[ c^d \omega _d \tilde{\tau }_L^{d+1} \le c^{d+1}+ (d+1) c^d \left( \log (c^{d^2/(d+1)} (d+1)^{1-d} ) + u + z_L \right) + y_L c^d \right] \\ =&\mathbb {P}\left[ \omega _d \tilde{\tau }_L^{d+1} \le c+ (d+1) \left( \log (c^{d^2/(d+1)} (d+1)^{1-d}) + u + z_L \right) + y_L \right] \\ =&\mathbb {P}\left[ \omega _d \tilde{\tau }_L^{d+1} \le d(d+1) \log L - \log \omega _d + d^2 \log c + \log ((d+1)^{1-d^2} ) + (d+1)u + z'_L \right] , \end{aligned}$$

where we set $z'_L:= (d+1)z_L + y_L = o(1)$ as $L \rightarrow \infty$. For some $z''_L$ tending to zero, this probability equals

$$\begin{aligned} \mathbb {P}\left[ \omega _d \tilde{\tau }_L^{d+1} \le d(d+1) \log L + d^2 \log \log L + \log \left( \frac{d^{d^2}(d+1)^{d^2}}{ (d+1)^{d^2-1} \omega _d} \right) + (d+1) u + z''_L \right] , \\ = \mathbb {P}\left[ \omega _d \tilde{\tau }_L^{d+1} - d(d+1) \log L - d^2 \log \log L \le \log \left( d^{d^2}(d+1)/ \omega _d \right) + (d+1) u + z''_L \right] . \end{aligned}$$

By Eq. 7 from Proposition 2.1, and the continuity of the limiting cumulative distribution function in that result, this probability tends to

$$\begin{aligned}&\exp \left( - c_d \left( \frac{(d+1)^{d^2}}{d^d \omega _d} \right) ^{1/(d+1)} e^{-u} (d^{d^2}(d+1))^{-1/(d+1)} \omega _d^{1/(d+1)} \right) \\&= \exp \left( - c_d d^{-d} (d+1)^{d-1} e^{-u} \right) = F(u). \end{aligned}$$

In other words, we have derived Eq. A1 from Proposition 2.1.

## Footnotes

1 https://www.youtube.com/playlist?list=PLiaV5rk6Gk7rh6nvAts1w4WZrHE0TKeW2

2 https://www.youtube.com/playlist?list=PLiaV5rk6Gk7qEXpLOU7FSvN4b8dy1_GJn
